# Revision of the cultural chronology of precolonial Puerto Rico: A Bayesian approach

**DOI:** 10.1371/journal.pone.0282052

**Published:** 2023-02-22

**Authors:** Reniel Rodríguez Ramos, Miguel Rodríguez López, William J. Pestle

**Affiliations:** 1 Programa de Ciencias Sociales, Universidad de Puerto Rico, Recinto de Utuado, Utuado, Puerto Rico; 2 División de Artes Liberales, Universidad Ana G. Méndez, Recinto de Gurabo, Puerto Rico; 3 Department of Anthropology, University of Miami, Coral Gables, FL, United States of America; German Archaeological Institute: Deutsches Archaologisches Institut, GERMANY

## Abstract

Puerto Rico has played a pivotal role in the building of cultural chronology for the insular Caribbean, and yet little systematic work has been conducted in recent decades to assess the validity of the system(s) produced. To resolve this issue, we assembled a radiocarbon inventory comprised of more than a thousand assays, drawn from both published sources and grey literature, which was used to assess and revise (as necessary) the received cultural chronology of Puerto Rico. The application of chronological hygiene protocols and Bayesian modeling of the dates yields an initial arrival of humans to the island more than a millennium earlier than previously established, making Puerto Rico the earliest inhabited island of the Antilles, following Trinidad. The chronology of the different cultural manifestations that have been identified for the island, as grouped by Rousean styles, also is updated, and in some cases heavily modified, as a result of this process. While admittedly limited by several mitigating factors, the image that emerges from this chronological revision suggests a much more complex, dynamic, and plural cultural scenario than has been traditionally assumed, as a result of the myriad of interactions that took place between the different peoples that coexisted in the island through time.

## Introduction

The archaeology of the Caribbean has undergone profound changes over the past two decades. With the application of new analytical techniques, the scope and resolution of available data has been greatly improved, fomenting new ideas regarding phenomena that include: the introduction of agriculture and pottery production into the islands, the degree of inter-island mobility of the archipelago’s Indigenous inhabitants, and the dynamics of their interactions between islands as well as with people of the surrounding continents. Although great strides have been made in the study of different aspects of these Indigenous societies, there are fundamental matters that are yet to be addressed adequately. Among these, one of the most salient is the absolute chronology of the vast array of cultural manifestations registered during the eight millennia of human occupation of the insular Caribbean.

**Fig 1:**
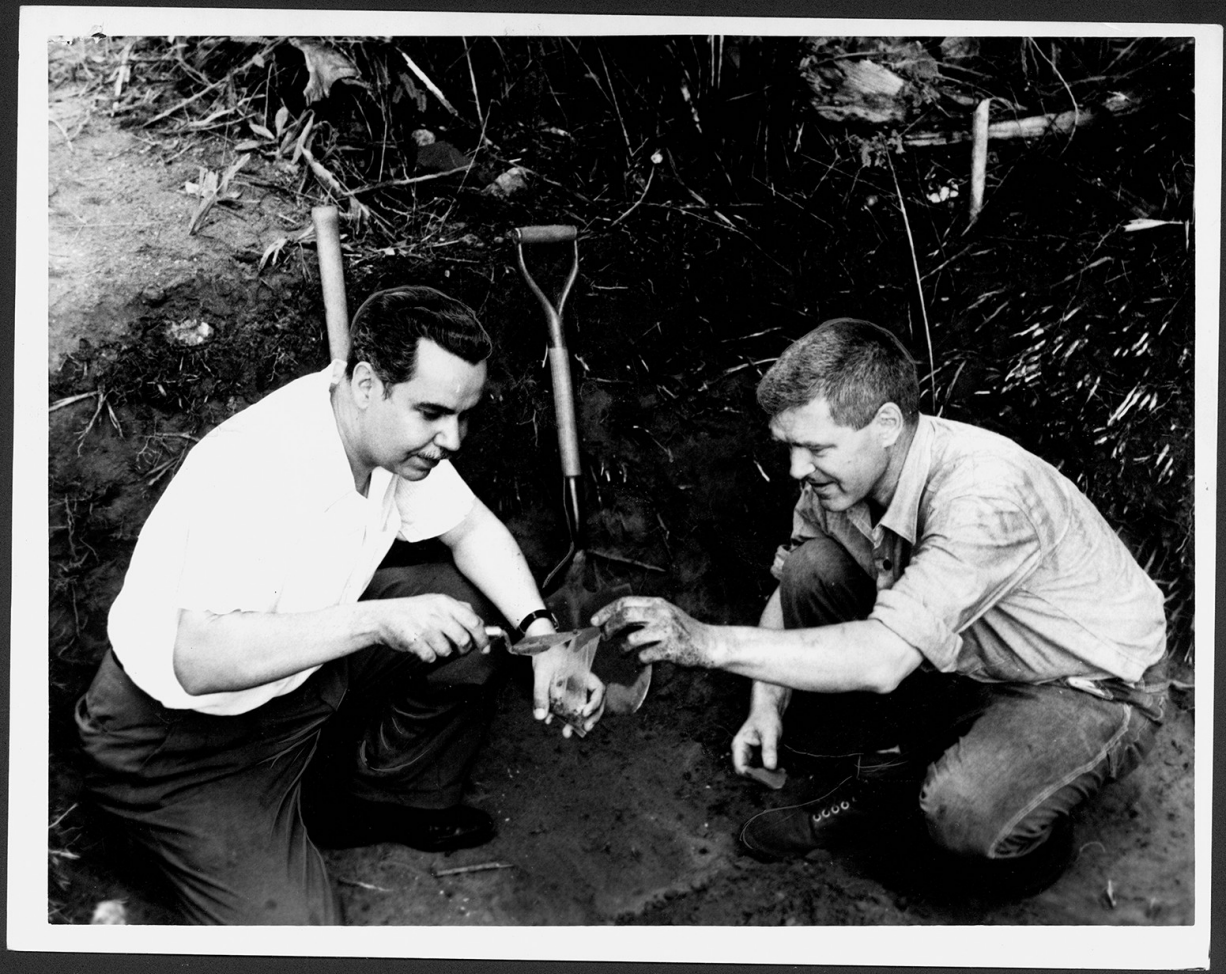
Ricardo Alegría (left) and Irving Rouse (right) obtaining radiocarbon dates from María de la Cruz Cave, Loíza, July 13, 1962. Note bulk sample size and methods of sample collection, both of which would affect the dates obtained. Image available at *Biblioteca Digital Puertorriqueña*, https://upr.contentdm.oclc.org/digital/collection/ELM4068/id/408/rec/16).

**Fig 2:**
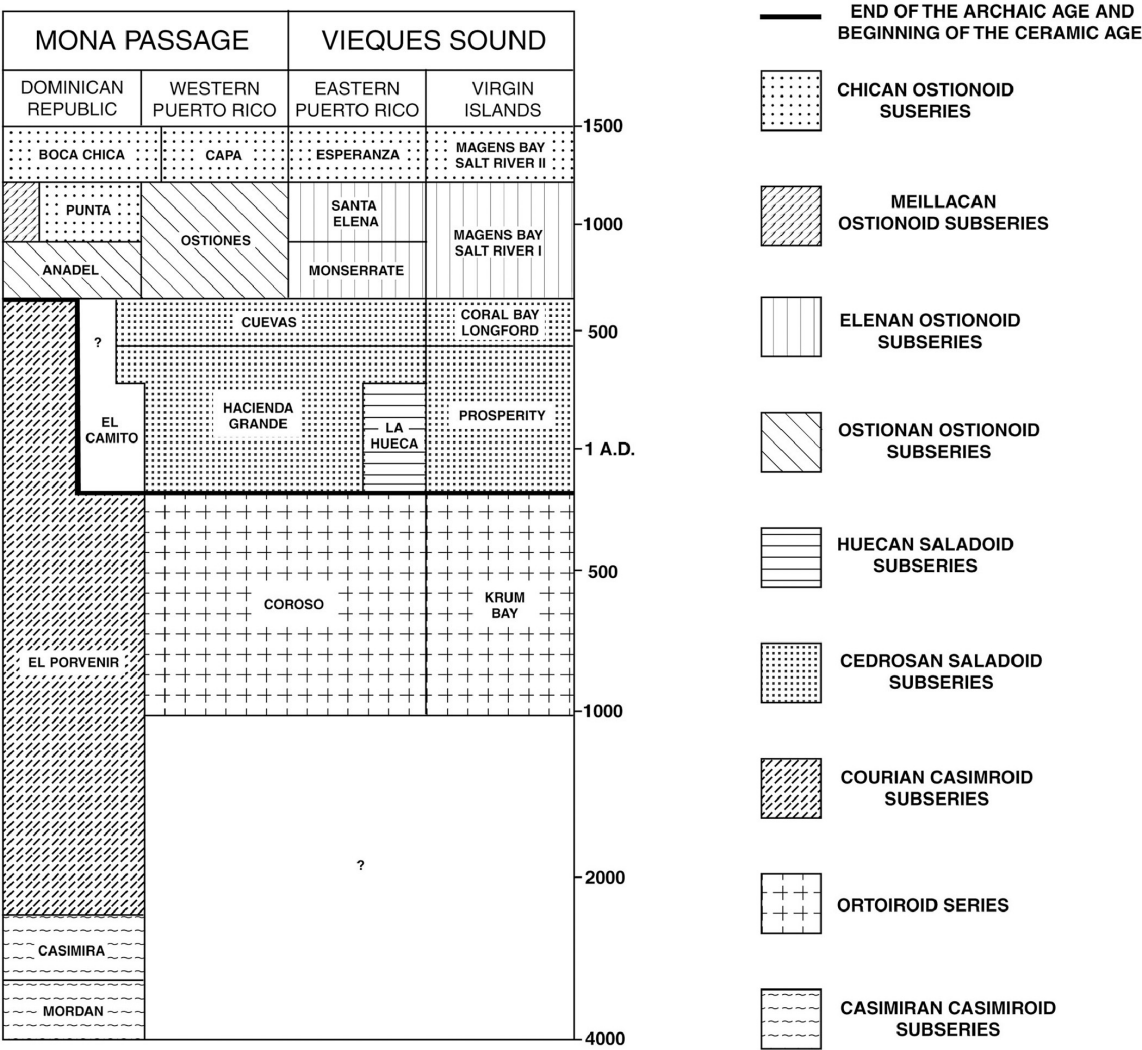
Rouse’s culture-chronology scheme for Puerto Rico. For explanation of categories/terms, see section below entitled, “A brief history of time in Puerto Rican archaeology”. Drawn by Jill Seagard, after Rouse [4].

This dearth of attention paid to the temporal dimension of the diverse cultural traditions documented in the Antilles is particularly notable in Puerto Rico, an island that played a key role in the articulation of the main culture-historical framework used across the insular Caribbean, that was developed by the late Irving Rouse. The chrono-cultural sequence of this model was initially based on fieldwork he conducted between 1936 and 1938 for the Scientific Survey of Puerto Rico and the Virgin Islands [1], under the auspices of the New York Academy of Sciences. The relative cultural sequence postulated in this first version of Rouse’s model was later provided an absolute temporality based on the results of one of the first radiometric studies performed in the Antilles [2], which he conducted together with Ricardo Alegría, founder of the *Instituto de Cultura Puertorriqueña* (Fig 1). This pioneering work consisted of 13 radiocarbon dates obtained from sites associated with the different cultural manifestations that Rouse had previously identified on the island [3]. Later iterations of this framework included a wider selection of dates generated by other researchers, which eventually led to the production of the version of scheme [4] that is still the most used culture-historical framework in Puerto Rico and the rest of the insular Caribbean today (Fig 2).

Although Rouse’s model has provided the main culture-historical platform upon which most archaeological research on the island and in the region has been framed, the absolute chronology it defines for different cultural manifestations in Puerto Rico has not undergone revision, despite the vast amount of research that has been conducted in the island since its final postulation in 1992. With the objective of presenting a revised cultural chronology for Puerto Rico, we developed an inventory of radiocarbon dates containing more than one thousand entries, which is, by far, the largest number of assays available for any island in the Caribbean archipelago. Bayesian modeling of the available radiocarbon dates was conducted to provide a first approximation of an updated temporality for the different cultural traditions that have been identified in the island and to critically evaluate some of the culture-historical assumptions about the precolonial history of Puerto Rico that previously have been proposed.

### A brief history of time in Puerto Rican archaeology

The geographic position of Puerto Rico, located at the center of the Antillean arc, and its role as a cultural connector between the Greater and the Lesser Antilles, has made it a key context for the articulation of some of the main ideas about the origins and sequence of occupations of the peoples that migrated to, and developed in, the insular Caribbean. During the initial period of archaeological work in the island in the late 19^th^ century, it was invariably assumed that its Indigenous presence was limited to the first societies of the Americas that resisted Spanish infringement, commonly known as the Taíno. The initial temporal approximations for these Indigenous groups assumed a rather late incursion into the island, while also inserting these societies into a typological time scale based on the purported stage of their development, as established in the schemes of social and cultural evolution in existence at the time. For example, Coll y Toste [5:^34^–^35^], making use of the Thomsen’s Three Age System, indicated that the Taíno belonged to the Stone Age, particularly to the period of “polished stone” [see also 6: for similar arguments]. This perception of the short-lived timeframe for the Indigenous occupations of the island remained unaltered in the first years after the invasion of Puerto Rico by the United States in 1898, which saw the arrival of other researchers with agendas focused on generating archaeological collections for their homeland institutions and evaluating the existing information about the island’s ancient inhabitants. This was the case, for example, of Jesse W. Fewkes [7] who, despite making some important contributions in the understanding of the Indigenous societies of the island, subscribed to previous notions of the lack of substantial time depth of its occupation.

The researchers that first began to suggest a deeper temporality for the Indigenous occupation of Puerto Rico were Spinden [8] [see also 9] and de Hostos [10]. Spinden conducted the first stratigraphic excavations in the island, at the site of Punta Ostiones, where he showed how the site’s different mounds had developed and discussed changes in pottery production in the different layers. Based mainly on his excavations at this same site, de Hostos [10:377] proposed a cultural sequence which he described as a “continuous, uninterrupted aboriginal habitation of these sites during generations”, based on the “relation between depth and the development of industrial arts, as shown in the quality of the relics found. The deeper the layer, the lower the quality.”

This idea that ceramic technology had gradually developed over time from coarser to finer forms in Puerto Rico was turned upside down with the work of Rainey [11], who argued for the presence of two distinct cultural manifestations, an initial one characterized by an emphasis on the consumption of crabs and the production of painted pottery, and a later one that favored mollusks and other marine resources together with the manufacture of coarser pots. For him, these two manifestations represented sequential migrations into the island from South America. This has been called the “Crab/Shell dichotomy”. As he noted:

A study of the collections has resulted in the conclusion that two prehistoric cultures are represented. Furthermore, it is probable that some time elapsed between the two periods of occupation since traits characterizing the early culture were not carried over or combined with traits characterizing the late culture [11:189].

In 1936, Irving Rouse arrived in Puerto Rico and quickly became the leading actor in the creation of what would become the dominant culture-historical sequence in the island and the rest of the Antilles. Based on his research on a wide range of archaeological sites from different parts of the island, he argued against Rainey’s ideas of two distinct migrations of ceramic-bearing groups into Puerto Rico [12]. Rather, he proposed that a single population movement of agro-ceramist groups had occurred, which, after going through a transitional period, eventually gave rise to what Rainey termed the Shell culture.

In later publications, Rouse [1, 13] desisted in using the concepts of Crab and Shell cultures proposed by Rainey, and rather began naming cultures based on the type sites where he had documented distinct pottery styles. These styles, each of which was indicative to him of a different culture, were grouped into a relative time framework that consisted of four periods, some of which had an early and a late component. This framework began with what was, at the time, tentatively considered the initial occupation of the island by the Coroso culture in Period 1, the Cuevas in Period 2, Ostiones and Santa Elena in Period 3, and Capá and Esperanza in Period 4 [1, 14]. Given that no techniques for absolute dating yet were available, Rouse tried to provide calendric age ranges for the different cultural manifestations based on the depths of the deposits where the different styles were found, and an assumed accumulation rate for each of them. On this basis, Rouse [1:564–566] suggested that Period 1 lasted from AD 849–929, Period 2 from AD 929–1193, Period 3 from AD 1193–1437, and Period 4 from AD 1437–1584, the latter date corresponding with the Indigenous abandonment of Mona Island, which he considered to be the last place of occupation of Taino societies in the Puerto Rican archipelago.

In 1962, with the advent of radiocarbon dating, in collaboration with Ricardo Alegría, and under the auspices of the National Science Foundation, Rouse collected the first 12 charcoal samples obtained from the island for the purposes of absolute dating (Table 1). Together with an additional sample from a wooden post from Caguana previously excavated by Alegría, these 13 assays were analyzed by Stuiver [15] at the Geochronometric Laboratory of Yale University. Dates were obtained from eight sites in association with the different cultural manifestations that had been defined at that time (Fig 3 from [2]). During this initial part of this chronometric research, Rouse and Alegría paid particular attention to Puerto Rico because, “it is strategically located in the center of the West Indies, providing a base from which the chronology could subsequently be extended both east and west,” and, “it has the best worked out relative chronology, yet no absolute dates had previously been obtained.” [2:2, see also 3]. This chronology, which was comprised of neatly ordered and non-overlapping cultural manifestations, served as a temporal basis in the official narratives of Puerto Rican Indigenous history through the work of Alegría as head of the Institute of Puerto Rican Culture. With the incorporation of nine additional dates obtained from three sites [16] and then others from seven additional sites [17], including a thermoluminescence date from the Canas site obtained by J. José Ortiz Aguilú, Rouse and Alegría [18:78] articulated a general chrono-cultural model for Puerto Rico and the Lesser Antilles, which served as a prelude to the final version of the framework that also included the rest of the Greater Antilles and the Bahamas that was published a couple of years later [4:^52^–^53^].

**Fig 3:**
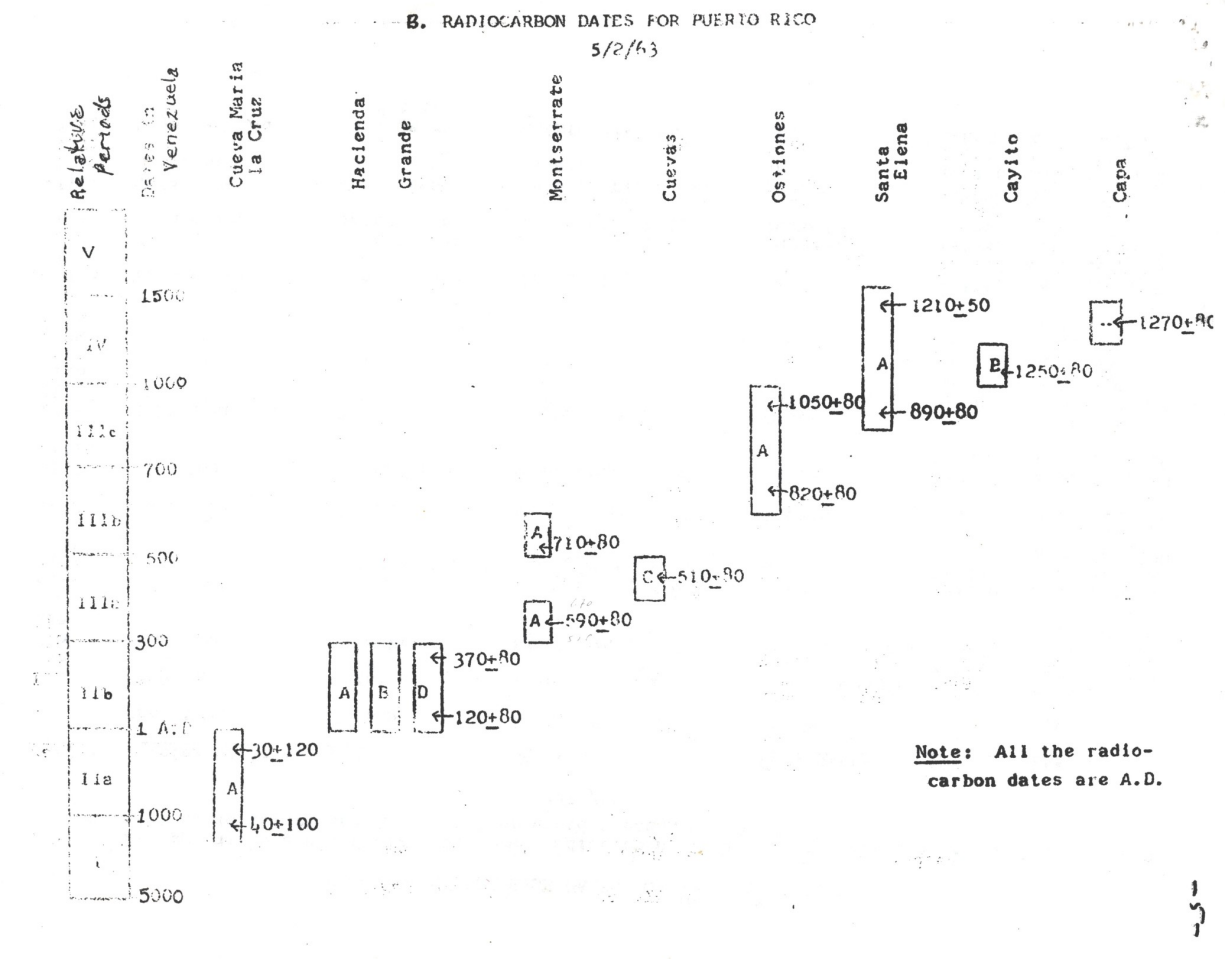
Plot of thirteen original radiocarbon dates obtained from Puerto Rico as presented by Rouse and Alegría in 1963 [2].

**Table 1:** 

Site	Style	Radiocarbon Date (BP)	Sample number	Calibrated Date (2-sigma)
Cueva Maria de la Cruz	Coroso	1910±100	Y-1235	150 BC–AD 360
Cueva María de la Cruz	Coroso	1920±120	Y-1234	200 BC–AD 400
Hacienda Grande	Hacienda Grande	1830±80	Y-1233	AD 30–400
Hacienda Grande	Hacienda Grande	1580±80	Y-1232	AD 260–640
Monserrate	Cuevas	1360±80	Y-1237	AD 550–880
Monserrate	Ostiones	1240±80	Y-1236	AD 660–980
Cuevas	Cuevas	1440±80	Y-1240	AD 430–770
Punta Ostiones	Ostiones	1130±80	Y-1242	AD 680–1040
Punta Ostiones	Ostiones	900±80	Y-1241	AD 1000–1270
Santa Elena	Santa Elena	1060±80	Y-1239	AD 770–1160
Santa Elena	Capá	740±80	Y-1238	AD 1050–1400
Cayito	Boca Chica	700±80	Y-1243	AD 1180–1410
Capá (i.e., Caguana)	Capá	680±80	Y-1244	AD 1220–1420

Radiocarbon dates obtained in 1962 by Rouse and Alegría [2]. The stylistic attributions are based on Stuiver [15: citing Rouse] and Rouse and Alegría [3].

### The received Rousean culture-chronology

Rouse’s approach to building a cultural chronology for Puerto Rico and the broader Caribbean proceeded as follows:

I form styles or complexes of pottery and other artifacts, each of which is indicative of a single people and culture, and plot their distribution on chronological charts in order to determine their units in time as well as in space. Then I group the styles into series and, following the lead of the late Gary Vescelius, have begun to divide the latter into subseries. I plot the series and subseries on my charts in an effort to distinguish the successive waves of peoples that have engulfed the Antilles during the Lithic, Archaic, and Ceramic Ages [4:59].

This ultimate iteration of Rouse’s model for Puerto Rico established that the island was first occupied by people of the Coroso culture (Ortoiroid series) at around 1000 BC. This cultural manifestation belonged to what Rouse termed the Archaic Age, a concept derived from Willey and Phillip’s [19] developmental scheme for American archaeology (Fig 2). At 200 BC, Rouse postulated that there was another migration of peoples belonging to the Saladoid series, the first series of the Ceramic Age, which he divided into two subseries, Cedrosan and Huecan, the latter of which was restricted to the eastern part of the island and Vieques (Rouse named cultural units “above” that of style using the name of type sites appended with the suffix “-an” for subseries and “-oid” for series). The La Hueca style of the Huecan Saladoid disappeared by AD 200, while the Hacienda Grande style of the Cedrosan Saladoid evolved into the Cuevas style by AD 400. This Cuevas style transformed, by AD 600, into the Monserrate style in the eastern part of the island, while in the west it changed into the Ostiones style. These two styles are the first manifestations of new subseries, Ostionan in the west and Elenan in the east, of the Ostionoid series. At AD 900, in the eastern part of the island the Monserrate style gives way to the Santa Elena style, which by AD 1200 transforms into the Esperanza style of the Chican Ostionoid. In the western part of the island, the Ostiones style develops into the Capá style (also a manifestation of the Chican Ostionoid), while there is also a slight projection of the Boca Chica style from the Dominican Republic in this part of Puerto Rico during this time.

Although the development of this model constituted a foundational achievement in the archaeology of Puerto Rico, it suffered from fundamental weaknesses that still, to some degree, plague absolute dating of archaeological sites on the island. First, the available evidence shows that the protocols used in obtaining the initial samples on which the cultural chronology was built were inadequate, as different charcoal fragments were mixed in the same bags, while also having been collected in ways that might have led to their contamination (see Fig 1). As noted by Rouse and Alegría [18:^56^] “we obtained these samples by picking scattered bits of charcoal out of entire excavation units.” These units were excavated in 25 cm arbitrary levels, which obviously limits the stratigraphic resolution of the collected samples. In addition, none of the dates used in the model were calibrated, having instead been derived by subtraction of their radiocarbon ages from AD 1950, the stipulated “present” in years “before present”, a practice that persists in the work of some archaeologists on the island today. Furthermore, the durations of different manifestations were obtained by adding/subtracting the measurement error of the dates from their mean value, a practice that manifestly misunderstands the meaning of the radiocarbon results obtained. As expected, when calibrating the dates originally obtained by Rouse, it is immediately evident that, in most cases, the resulting ranges do not fit with the temporalities presented in his chrono-cultural framework. For instance, Rouse’s calibrated dates for Coroso extend at least 400 years later than he assumed while those for the Cuevas are at least 300 years later than expected. Finally, the number of dates used in building his model was far too limited, raising issues of the representativeness of the chronologies expressed for each of the styles, given the large number and significant diversity of sites present on the island.

It should be noted that, in addition to the chronological revision that the model requires, there are many conceptual aspects of it and its construction that have been subjected to scrutiny in the past several decades [e.g. 20–27]. However, despite the issues that have been raised regarding the premises of this model and the definition of the different styles, the cultural categories that were devised by Rouse continue to be used as a *lingua franca* in Puerto Rican and Caribbean archaeology, and their assumed temporalities still too often serve as the foundation upon which archaeological interpretations are made. Thus, the radiocarbon database that has been developed in this study, and the modeling of the dates compiled, has the aim of testing and revising the chronometric dimensions of the different manifestations that were defined by Rouse using a suite of analytical tools that were unavailable to him when he constructed his model decades ago.

## Materials and methods

### The database

The dates that make up this inventory are the result of a vast amount of work conducted by local and foreign researchers in the island over the past half century. Most of the dates were obtained from published works, although a significant portion were also drawn from so-called “grey literature,” mainly cultural resource management (CRM) reports available at the *Consejo de Arqueología Terrestre de Puerto Rico* and the *Oficina Estatal de Conservación Histórica de Puerto Rico*. In addition, archaeologists that have worked, or are conducting, research in the island were contacted to determine if they had any additional dates not listed in the inventory, and to corroborate if the information that was provided with their published dates was correct. No permits were required for the described study, which complied with all relevant regulations.

Ultimately, this research and surveying resulted in the identification of a total of 1008 radiocarbon dates (S1 Table). For each of the dates, we recorded contextual information (site, municipality, excavation unit, stratum, level, feature), cultural affiliation (series, subseries, style), lab number, material dated, taxon (when available), dating method (AMS, conventional, extended counting), comments, and bibliographic information, if available.

**Fig 4:**
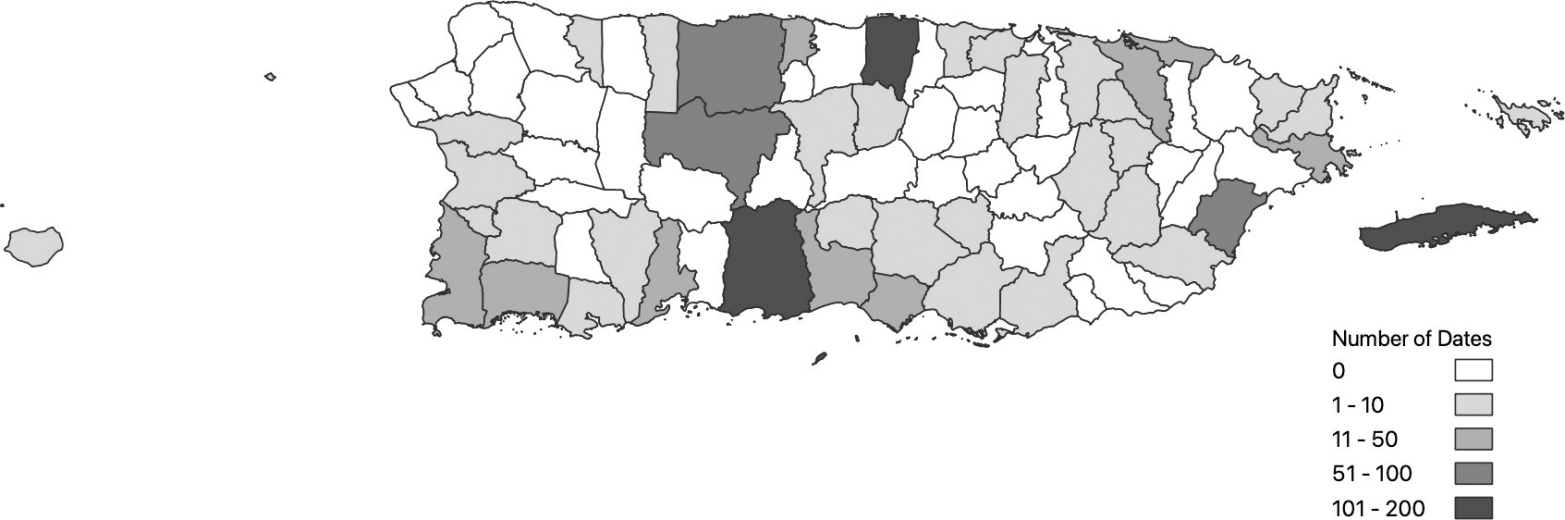
Number of radiocarbon dates in the database (S1 Table) by municipality.

In the development and analysis of the database, several issues became readily apparent. Some of these issues are mitigated (to such a degree as possible) by the methods detailed below, but their existence must be acknowledged. First, there are marked disparities in the methods and levels of analysis used for the attribution of dates to particular cultural manifestations (series, subseries, styles) by different researchers, a lack of standardization that is, ultimately, irresolvable. Indeed, some researchers used cultural categories that were different from those in Rouse’s framework, which limited greatly their utility to the present work. Second, there are notable differences among projects and investigators in the contextual resolution (provenance) of the deposits from which dated materials are derived. Another issue is the asymmetric spatial distribution of dates across the island and among different sites. For instance, while for municipalities of the north-central part of the island, such as Vega Baja, Utuado, and Arecibo, there is an ample set of dates, in municipalities of the northwest, including Rincón, Aguada, Aguadilla, and Isabela, which are rich with sites, there is a complete dearth of dated contexts (Fig 4). In fact, out of the 78 municipalities of Puerto Rico, 33 are completely devoid of radiocarbon dates. On the same token, while there are sites such as Maisabel, Paso del Indio, Tibes, Punta Candelero, and La Hueca-Sorcé that have many dozens of dates, there are numerous other sites from which only one date has been obtained. Indeed, this is the case of Caguana, one of the most complex sites in the island and the rest of the insular Caribbean, for which a single radiocarbon assay has been produced. This phenomenon is not unique to Puerto Rico; in fact, there is a pan-Antillean pattern in which many sites, or even entire islands, have few or no radiocarbon dates [28, 29]. Finally, there are dates that are known to be missing from this database, despite having been obtained more than a decade ago, because they remain unpublished or because the reports in which they were presented are unavailable. Combined, these factors make clear that the findings of the present study should be regarded as an initial approximation at a revised chronology for the island, one which should be further refined as new dates become available.

In order to enhance the utility of the analyzed dates, prior to modeling, a chronometric hygiene approach was employed, using as a basis the protocols established for the Caribbean record set forth by Fitzpatrick [28] and Napolitano et al. [29], a practice that has become a standard in archaeological chronometry in many parts of the world. In this approach, each date was assigned a score of 1 to 4 based on their relative chronological reliability. Category 1 was used for dates from terrestrial plant and animal species identified to taxon and dated with AMS, with all required provenience information provided; Category 2 corresponded to charcoal dates without taxonomic identification, dated shell identified to taxon, and shell artifacts, all of which have provenience information and laboratory processing numbers; Category 3 included shell dates not identified to taxon, those with incomplete contextual information, and samples with uncalibrated ages of 300 bp or younger, and Category 4 contained for those assays that lacked contextual information, laboratory processing number, were deemed problematic by the authors, or were labeled as modern by the laboratories. All chronometric hygiene assessments are noted in S1 Table. In total, 17 of the 1008 compiled dates (1.7%) were assigned to Category 1, while 806 (80.0%) were included in Category 2. While this number appears low, it should be noted that in Napolitano et al. [29] only ten of 2400+ dates (less than 0.5%) from throughout the Caribbean belonged to Category 1, making the Puerto Rican date assemblage appear robust by comparison. Only dates from Categories 1 and 2 were considered for further analysis. It should be noted that in the construction of our chronology we did not include dates derived from rock art; these are discussed in detail elsewhere [30–32].

After the assessment of chronological hygiene, we applied an additional filtering process, which we termed “chronological fitness”, to the remaining 823 Category 1 and 2 dates. This second phase of categorization was deemed necessary given the aforementioned disparities in the precision of the information available for some of the dates falling in hygiene Categories 1 and 2. This step required the analysis of the contextual information and the analytical accuracy of the pottery assemblages that was reported for all dated contexts, and thus served as the basis for us to determine the suitability of each assay for dating the styles as determined by the individual excavators. A two-tiered categorization was devised, with Fitness Class 1 reserved for dates that possessed a clear stratigraphic association with contexts presenting diagnostic artifact assemblages and Class 2 being for those that lacked such an association. Of the 823 dates that fell into hygiene Categories 1 and 2, a total of 614 dates (74.6%) were placed in Fitness Class 1 and were selected for subsequent Bayesian analysis. As with chronometric hygiene, Fitness Class designations for each date are provided in S1 Table.

### Modeling methods

Calibration and chronological modeling were performed using OxCal v4.4 [33]. While all individual dates were calibrated (without any model structure), only those dates in Fitness Class 1 were forwarded for modeling by Rousean style. Furthermore, we only attempted modeling of styles with at least twelve dates from at least two different sites. All told, ten individual style models, comprising 459 dates, were attempted (not including “mixed” styles or the “All Ostiones” style model, see discussion below).

All terrestrial samples (excluding human bone samples) were calibrated using the Intcal20 curve [34]. Marine samples were calibrated using the Marine20 curve of Heaton and colleagues [35], with regionally appropriate ΔR values applied [following 36]. For samples originating from the northern half of Puerto Rico, we employed a ΔR of -241±81, whereas the ΔR value used for samples from southern Puerto Rico was -138±23. ΔR values were adjusted for the Marine20 curve. All human bone samples (and AA-72879, a canine bone sample from Punta Candelero) were calibrated using a mixed terrestrial/marine curve with an a priori stipulation of 30±15% marine carbon. As with the marine samples, regionally appropriate ΔR values were employed for these bone samples (see S2 File for a discussion on the calculation of the ΔR values and the calibration of bone samples).

At the initiation of each style model, all fitness level “1” dates associated with a given style were grouped (using the differentiated calibration parameters stipulated above) into a named style by means of OxCal’s “overlapping phase” option. No outlier treatment was applied prior to the initial model iteration. After completion of the first instantiation of each style’s Markov Chain Monte Carlo model, any individual date with a calibrated range extending beyond the range of the calibration curve (i.e., very recent dates) were removed. In total, nine such dates (2.0%) were removed from the initial sample of 459 Fitness Class 1 dates.

Subsequent to a second model instantiation, the integrity of each resulting phase model was assessed by reference to its agreement indices (Amodel and Aoverall). If either value fell below the recommended threshold of 60.0 [33, 37], individual dates with agreement indices of less than 60.0 were removed and the style model was re-run. This process was repeated iteratively until both Amodel and Aoverall values for each style exceeded 60.0. This iterative process removed a further fourteen dates (3.1%) from the remaining sample of 450.

In total, 95.0% of the initial 459 Fitness Class 1 dates were included in the final ten style models. The attributes of each style model are presented below in Table 2. Individual style models included between 10–100 dates (average 44), representing from 73.3–100.0% of the initial Fitness Class 1 dates for each style. The ten style models included between 4–24 sites each, with an average of nine sites per style. All these parameters and model indices are reported on a style-by-style basis below. In our discussion, a distinction is made between two sigma calibrated (calBC/calAD) results and the modeled/calibrated ranges (mcalBC/mcalAD) for both individual samples and phase boundaries in all resulting tables and figures. This is an important distinction, as the modeled dates only have inherent value when considered within the models that were run for each of the styles. Put differently, the modeling process imposes structure (priors) on the dates that is only appropriate when the dates are considered in aggregate. Finally, all calibrated and modeled/calibrated dates were rounded to the nearest decade following Millard [38].

**Table 2:** 

Style	Fitness 1 dates	Modern removed	Poor agreement index removed	Final number of dates	Amodel	Aoverall	No. of sites	Modeled boundary start date (mcal)	Modeled boundary end date (mcal)
Coroso	86	0	0	86	144.0	115.3	24	4300–4050 BC	AD 130–430
Hacienda Grande	80	0	9	71	84.2	68.3	7	AD 50–200	AD 580–680
La Hueca	56	1	0	55	100.2	97.2	4	120 BC–AD 110	AD 1350–1540
Cuevas	104	1	3	100	189.2	151.2	14	AD 520–590	AD 1040–1100
Ostiones	58	1	0	57	103.9	104.5	14	AD 440–610	AD 1420–1510
-Pure Ost.	42	1	0	41	108.9	109.8	10	AD 400–590	AD 1420–1530
-Mod. Ost.	16	0	0	16	101.9	102.4	4	AD 730–960	AD 1280–1460
Monserrate	14	0	0	14	108.2	100.2	7	AD 590–860	AD 1020–1240
Santa Elena	29	2	2	25	115.2	108.3	10	AD 910–1030	AD 1280–1390
Capá	17	0	0	17	103.2	103.5	6	AD 1200–1280	AD 1410–1500
Esperanza	15	4	0	11	126.7	123.6	7	AD 880–1190	AD 1430–1640

Modeled dates for styles and their relative agreement indexes.

## Results

The following discussion will focus on the results of the modeling of the aggregated dates for each of the styles. We note explicitly that what we propose here is not a new chronometric dogma for the island, but rather a critical test and re-assessment of the received cultural chronology based on the existing data, which should be further revised as new information becomes available. For a summary of the modeled boundaries for each of the styles, as well as the number of dated sites and assays used for modeling each of them, refer to Table 2. Individual tables for each style are provided in S2–S12 Tables. Images of pottery representing each of the styles defined by Rouse are included in S1–S18 Figs.

### Coroso style

In the final conceptualization of Rouse’s model, the earliest inhabitants of Puerto Rico were termed the Coroso culture of the Corosan Ortoiroid subseries. Although a Casimiroid presence has been suggested for western Puerto Rico because of the documentation of a blade technology in some archaeological sites, thus far there have been no clear definition of distinct cultural manifestations during this early period of the island. Coroso, together with the Krum Bay style documented in the Virgin Islands, form the Corosan Ortoiroid subseries of the Ortoiroid series. In difference to the Krum Bay style, the Coroso lacks the production of celts, while being defined by the production of expedient flakes made of chert and metavolcanic materials, use-modified artifacts of which the most diagnostic is the edge-ground cobble (i.e., edge-grinders), extended burials, use of ochre, lack of pottery, and the production of beads and pendants of stone, bone, or shell. Sites of the Coroso culture were supposedly on or near the shores, as well as in caves. Originally, the peoples classified in this category were considered to be in an “Archaic” developmental stage, having also been referred to as preceramic or preagricultural. However, recent evidence for the production of pottery and the presence of cultivars in early contexts of the island have demanded a reassessment of many of the conceptions that had been developed about these early societies in Puerto Rico [24, 39–43].

**Fig 5:**
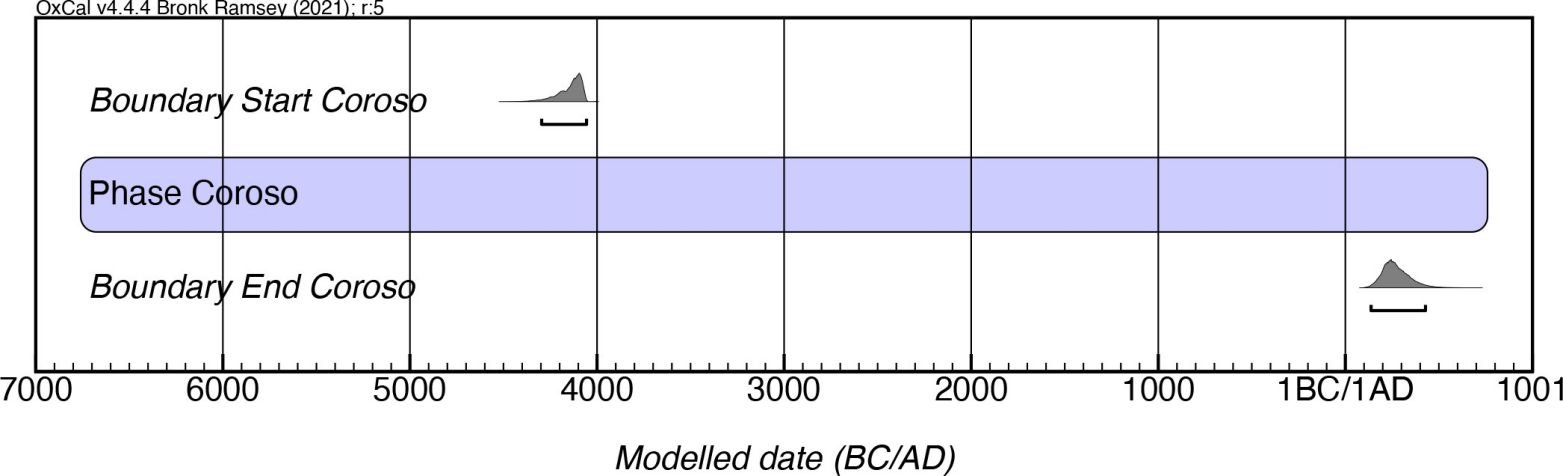
Modeled date ranges for the Coroso style in Puerto Rico. Probability distributions indicate modeled 2σ (95.4%) confidence intervals for beginning and end of phase.

Given the lack of distinct cultural manifestations in the island during this initial period of occupation, all of the sites that have been generically identified as “Archaic” are here considered as belonging to the Coroso style, with the expectation that in future studies the definition of the diverse cultural groups that existed in the island will be established. Rouse’s ultimate conceptualization of the Coroso for Puerto Rico [4] established a 600-year duration for this style, extending from 1000–400 BC. He based this chronology on seven dates obtained from three sites, namely Cueva María de la Cruz, Cayo Cofresí, and Caño Hondo. However, the analysis of the 86 dates from 24 sites that was hereby conducted indicates instead that the earliest cultural manifestation on the island began in the centuries preceding 4000 mcalBC (4300–4050 mcalBC) and persisted until the 5^th^ century mcalAD (Fig 5; S2 Table). While it is true that only two dates (UGAMS-52607 and UGM-30015) have individual calibrated ranges that predate 3000 calBC, both coming from the Cueva del Abono site in Utuado, individual agreement values for those two dates are nonetheless well above (73.0 and 104.2, respectively) the generally accepted 60.0 threshold value in our Bayesian analysis. As a result, these dates extend the initial occupation of the island far earlier than recently proposed by Napolitano and colleagues [29], who fixed the inhabitation of Puerto Rico to 4655–4305 mcalBP. The onset dates for Coroso might be pushed back even further if early dates, such as that of 5960±250 BP (Beta-29778, charcoal) from Angostura [44], can be corroborated and if anthropogenic activities such as those that documented in Laguna Tortuguero in northern Puerto Rico can be further verified [45].

It is important to note that, in contrast to the idea that these groups limited their occupation to the coastal areas, there is growing evidence for their incursion into the mountainous interior of Puerto Rico since the earliest phases of the occupation of the island in sites such as Cueva Ventana, Cueva del Abono, and Cueva Tembladera, among others. The dates for these manifestations also extend almost a millennia later than considered in Rouse’s model. This suggests a longer period of coexistence of these groups and the later arrivals, and refutes notions of their quick extinction, displacement, and/or assimilation as assumed in Rouse’s model.

### Hacienda Grande style

The Hacienda Grande style is the earliest manifestation of the Saladoid series in Puerto Rico, having purportedly been the result of a northward population movement from the mouth of the Orinoco River in Venezuela, via the Lesser Antilles. Pottery of this style is well-fired and characterized by the primary use of white-on-red painted anthropomorphic and curvilinear designs, with a secondary presence of polychromed and zoned-incised crosshatched wares. The most diagnostic vessels have an inverted bell shape, while also having ovoid jars with flat bases and open hemispherical bowls with annular bases made with a cream-colored paste, plain or flanged rims, and D-shaped strap handles. The matrix of the vessels is fine and hard, generally devoid of aplastic inclusions, with an average thickness of around 5-7mm. In some cases, modeled motifs of animal and human heads are noted in flanges, rims, or lugs. Griddles are a common occurrence. The plano-convex adze is the most diagnostic lithic artifact, while small three-pointers (triangular ideotechnic artifacts of stone, coral, shell or pottery) are also present, as is the production of personal adornments made of shell and different types of semi-precious stones. Sites associated to this style tend to be located near the coast, and often possess a horseshoe configuration.

**Fig 6:**
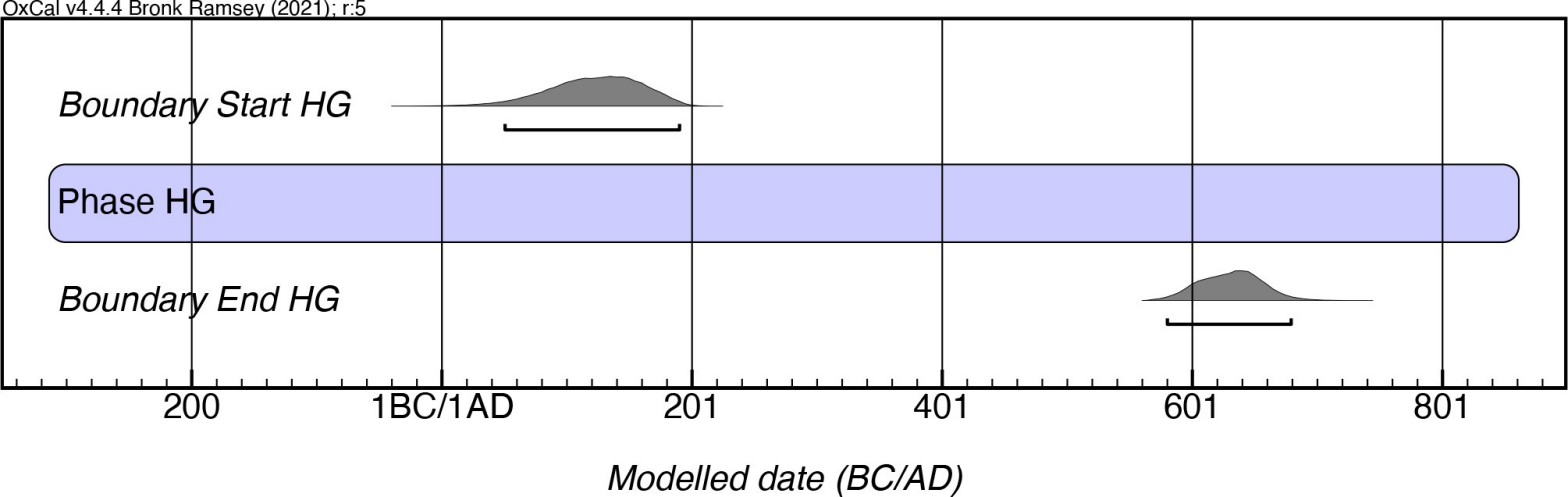
Modeled date ranges for the Hacienda Grande style. Probability distributions indicate modeled 2σ (95.4%) confidence intervals for beginning and end of phase.

Based on 26 dates obtained from seven sites, Rouse’s [4, 18] final chronological definition for Hacienda Grande proposed a range of 200 BC–AD 400. However, our modeling, which included 71 dates from seven sites, indicates instead a later onset (between mcalAD 50–200) and later persistence as well, until between mcalAD 580–680 (Fig 6; S3 Table). We would note, however, that two earlier iterations of the model, which yielded poor agreement indices (<60.0), did produce earlier modeled boundary start dates (300–170 mcalBC and 30 mcalBC–mcalAD 120, respectively). The sequential removal of individual dates with poor agreement indices disproportionally affected the boundary start date of Hacienda Grande, shifting it some three centuries later. We believe that the conservative estimate of a boundary start date arrived at in the final iteration of the model likely underestimates the first appearance of this style on the island of Puerto Rico, as noted in sites such as La Hueca-Sorcé, Tecla 1, and Maisabel, which have dates as early as 910–420 calBC (I-16153, charcoal), 770–230 calBC (I-13856, charcoal), and 750–120 calBC (Beta-14997, charcoal) respectively.

The earliest modeled dates for this style are recorded in La Hueca-Sorcé in Vieques, and in contemporaneous sites in north and south Puerto Rico such as Maisabel and Tecla 1. The temporal proximity of the modeled dates makes it difficult to ascertain which area of the island was settled first by the Saladoid migrants, but it seems that they quickly occupied widely separated coastal regions upon their arrival. The end dates for this style extend several centuries later after previously proposed, and Hacienda Grande ceramics are often found mixed with Cuevas style pottery until at least AD 800 in northern Puerto Rico and Vieques.

### La Hueca style

One of the most controversial cultural manifestations in Puerto Rico has been what Rouse termed the La Hueca style, which its discoverers, Luis Chanlatte Baik and Yvonne Narganes Storde, instead named the Huecoid (elevating it to the level of the Rousean series). The debate has centered on whether this manifestation comprises a distinct migration from South America or if it was derived from the Saladoid, from which it diverged in the northern Lesser Antilles. Irrespective of the particular position assumed about this matter, there is agreement in terms of the elements that characterize this cultural manifestation. Regarding its pottery, it is mostly decorated with zoned incised crosshatched designs, in some cases filled with white or pink pigments, as well as zoned punctuation. Major forms include open and inturned bowls, some containing modeled head lugs. Incense burners are also present as well as cassava griddles. The most salient feature of this manifestation is the noted emphasis in lapidary and microlapidary work using semiprecious stones, the production of adornments made of mother of pearl and *mullu* (the *Spondylus* shell), and the representation of a suite of animalistic forms in the personal ornaments the most distinctive being raptorial birds, batrachians (frogs), and curly-tailed animals, among others. La Hueca sites are also found on or near the coast, having both horseshoe and lineal configurations.

**Fig 7:**
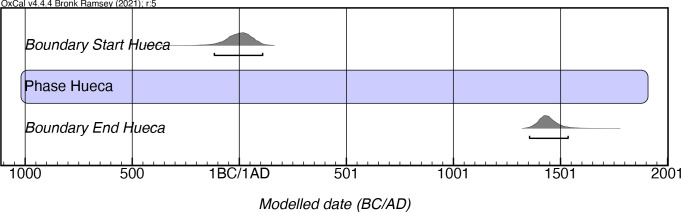
Modeled date ranges for the La Hueca style in Puerto Rico. Probability distributions indicate modeled 2σ (95.4%) confidence intervals for beginning and end of phase.

Rouse’s chronology placed manifestations of La Hueca between 200 BC and AD 200. Our model (which has its own caveats, see below) radically alters this proposed chronology, with La Hueca now extending from as early as the 2nd century mcalBC until the 16th century mcalAD (Fig 7; S4 Table). While these results are statistically robust and would appear to indicate a particularly long duration for material manifestations of this style, it also renders the stylistic attribution of La Hueca style of little use for most archaeological applications. The reasons for this chronological persistence of this style are not well understood and are one of the factors that prompted Oliver [46] to label this the “La Hueca Problem”. We would submit that the generation of further dates for this style, which could help determine whether this manifestation is, in fact, particularly long-lived, would be of great utility.

**Fig 8:**
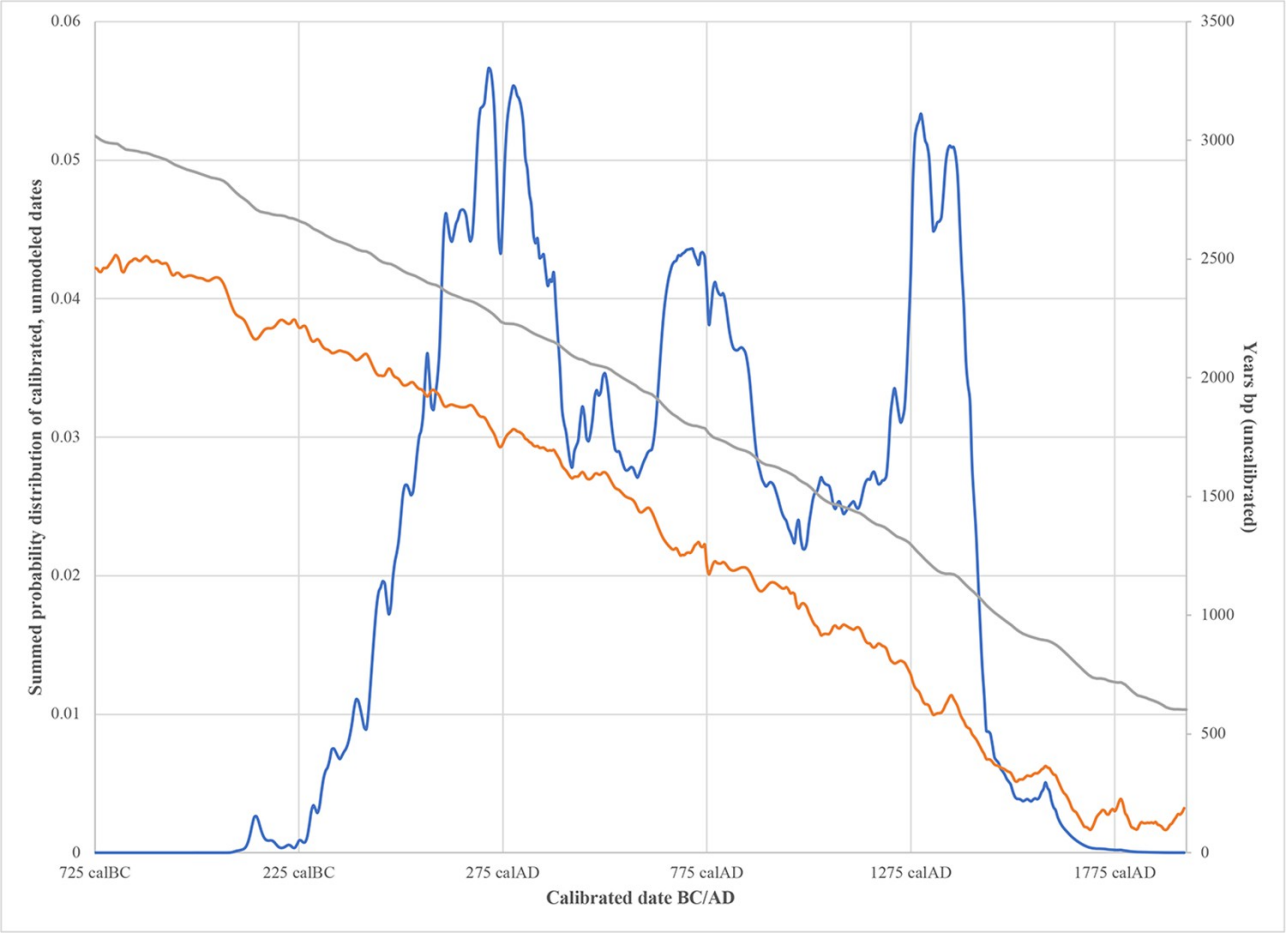
Trimodal distribution of La Hueca radiocarbon dates. Blue line represents summed probability distribution of all calibrated (but unmodeled) La Hueca radiocarbon dates. Orange and grey lines are Intcal20 and Marine20 calibration curves, respectively, presented to make clear that three peaks of combined La Hueca dates are consequence of actual distribution of dates, not an artifact of calibration.

It should be noted, however, that when looking at the distribution of the calibrated radiocarbon dates for this style, there is a multimodal distribution with three major peaks that may denote some sort of phasing to the style (Fig 8). Interestingly, this multimodal trend is noted in both main sites where this style has been documented, Punta Candelero and La Hueca-Sorcé. While the smaller peaks and valleys of the summed probability distribution can be explained by fluctuations in curve, the three larger peaks (ca. 120–400 mcalAD, 620–820 mcalAD, and 1270–1400 mcalAD) certainly cannot, being a consequence of the distribution of the dates, not the curve. Whether this distribution is a result of a sampling bias, an undocumented process of sample contamination, or the existence of an acceptable group of early dates followed by a problematic group of late dates, as has previously been suggested [e.g., 46], is still a pending matter to be resolved regarding this cultural manifestation.

Based on our modeling, it seems that the La Hueca style predates the Hacienda Grande by at least a century. This fits with the evidence obtained from the only contexts where these two manifestations have been found stratigraphically superimposed, at the site of Maisabel, where the La Hueca style lies under a layer of sterile sand below a Hacienda Grande deposit [see 47]. However, given the chronological issues noted with the La Hueca style and the early dates from the Hacienda Grande style that were excluded from our modeling, the temporal ordering of these two styles remains open to question.

### Cuevas style

The final stylistic definition of the Cuevas style included the production of unrestricted bowls with an infrequent use of polychrome painting, modeling, and incisions, with white-on-red painting being retained but in smaller proportions and representing mostly geometric designs, and a gradual disappearance of the use of paints (including white-on-red decoration), leaving, by the end, only monochrome painting and simple modeled face lugs [18, 48]. Sites associated with Cuevas pottery, which tend to retain a horseshoe configuration, begin to be located in the island interior and increase in number when compared to those possessing Hacienda Grande style ceramics.

**Fig 9:**
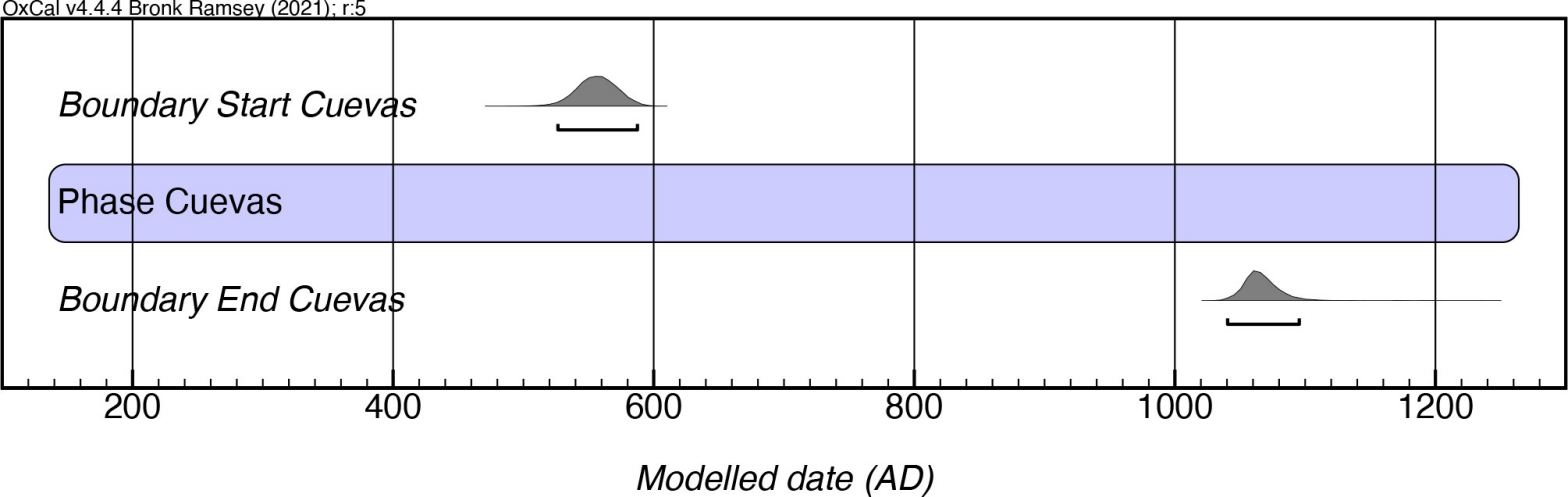
Modeled date ranges for the Cuevas style in Puerto Rico. Probability distributions indicate modeled 2σ (95.4%) confidence intervals for beginning and end of phase.

By 1992, the Rousean chronology of Cuevas had a range of AD 400–600 (uncalibrated), making it the style with the highest temporal resolution based on the results Rouse obtained of four dates from three archaeological sites. However, it should be noted that the earliest date for this style that was used by Rouse was actually obtained by Luis Chanlatte Baik from what he identified as an *Ostiones* deposit of the Punta Ostiones site (Chanlatte Baik 2014, personal communication). The results of our modeling of 100 dates, the most for any style in the present study, indicate a much longer-lasting existence of the Cuevas manifestation, beginning as early as mcalAD 520, and lasting until as late as mcalAD 1100 (Fig 9; S5 Table). While one date (Beta-283564) from Playa Blanca 6 has a low individual agreement value, the overall agreement of the model is exceptional, which underlines an apparent long chronology for this style. This suggests that the style emerged at least a century later than expected and remained in existence for half a millennium later that what was indicated in Rouse’s model. The late persistence of this style now explains the common occurrence of Cuevas pottery with styles which were deemed to be later, such as Ostiones, Monserrate, and Santa Elena. Rather than being “contaminated” contexts, as commonly assumed, this indicates that some of these styles were being produced concomitantly in the island. Findings such as this call into question the utility of these stylistic manifestations as temporal markers (while not invalidating their utility as different forms of ceramic production).

### Ostiones style

The Ostiones style has presented the most diverse set of categorizations in the archaeological literature of the island. While some researchers divide this category into a Pure or Early Ostiones for the earlier part, and a Modified or Late Ostiones for the later part, Rouse [4] never formally made that distinction in his chronological framework. He, however, did notice changes in this style through time, with an early version where red paint and slip is combined with orange and pink hues. Open bowls with straight sides are common with loop handles raised above the rim. The pottery continues to be thin, although coarser when contrasted to the Cuevas style. The decoration tends to be restricted to smudging or painting in confined areas or bands, with “simply modeled lugs and geometric figures modeled on vessel walls” [4:96]. The later version of this style emphasized the use of plastic decoration, particularly simple incised designs, and appliqué lugs on the rims of open bowls, on which red paint continued to be used.

**Fig 10:**
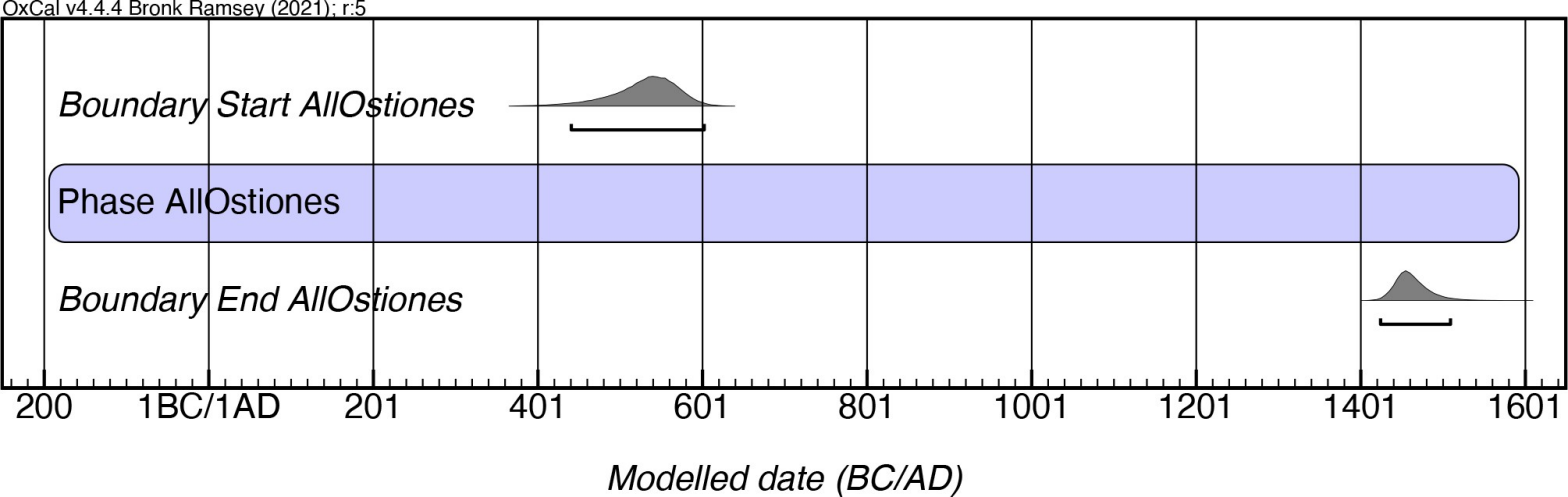
Modeled date ranges for the Ostiones style in Puerto Rico. Probability distributions indicate modeled 2σ (95.4%) confidence intervals for beginning and end of phase.

While Rouse postulated an overall duration of AD 600–1200 for the Ostiones style, our modeling of 57 dates from 14 sites indicates a longer duration, beginning as early as mcalAD440 and lasting more than a millennium until as late as mcalAD 1510 (Fig 10; S6 Table). The previously mentioned early Ostiones date from Punta Ostiones, together with dates from Hacienda La Victoria (PO-23) in Ponce, are the earliest assays for this style, dating more than a century earlier than the style’s prior proposed start date. This has important implications given that the initial dates for this style actually predate those of the style (Cuevas) from which, according to Rouse, it supposedly emerged. Whether this represents a sample bias or the fact that the origins of this pottery are different from its purported derivation from the Cuevas style, as had previously been suggested [e.g. 11, 20, 26], is a matter that deserves further evaluation.

**Fig 11:**
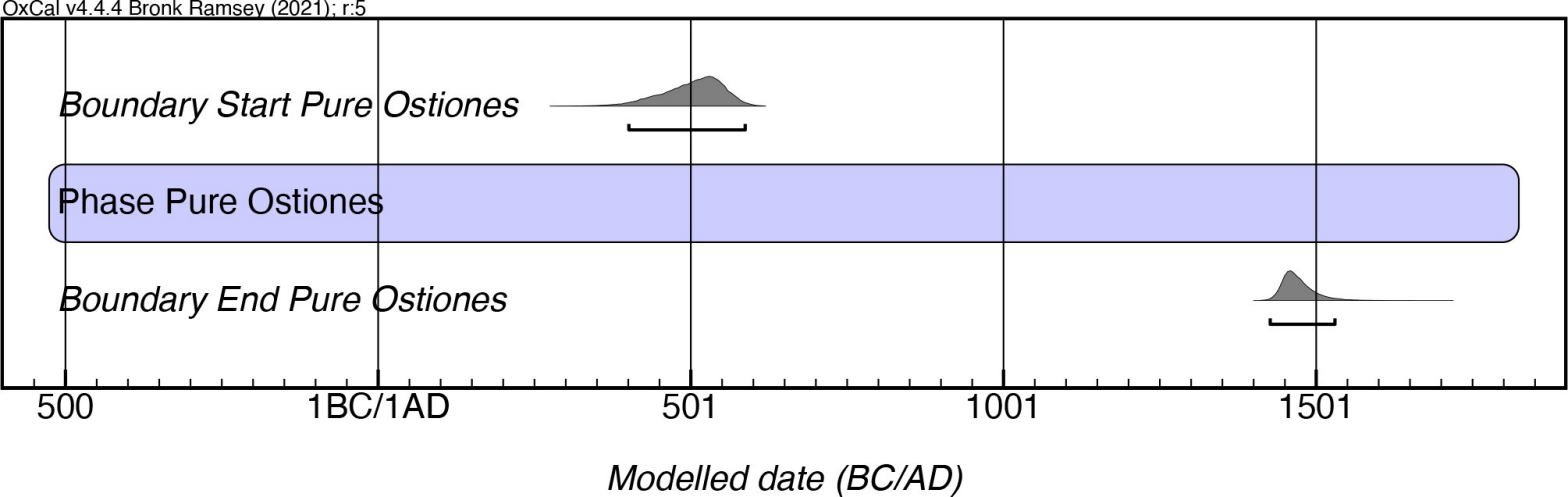
Modeled date ranges for the Pure Ostiones manifestation in Puerto Rico. Probability distributions indicate modeled 2σ (95.4%) confidence intervals for beginning and end of phase.

If one divides this style into an early (Pure) and a late (Modified) phase, the results are as follows. While Rouse stipulated the temporal extent of Pure Ostiones as lasting from 600–900 AD, the results of our modeling of 41 Pure Ostiones dates indicates a far longer period of existence, from as early as mcalAD 400 until mcalAD 1530, a period of over a millennium (Fig 11; S7 Table). The latest dates for this style come from El Parking site in southern Puerto Rico and Cueva Juan Miguel in the northern mountainous region of the island. Whether the continuous presence of this style reflects the persistence of a “limited selection of from the full range of forms” of the Ostiones style in later contexts as Oliver and Narganes [49:232] have noted, or if it is because of its continued production as a distinct style by its cultural bearers is another issue that is raised by these results.

**Fig 12:**
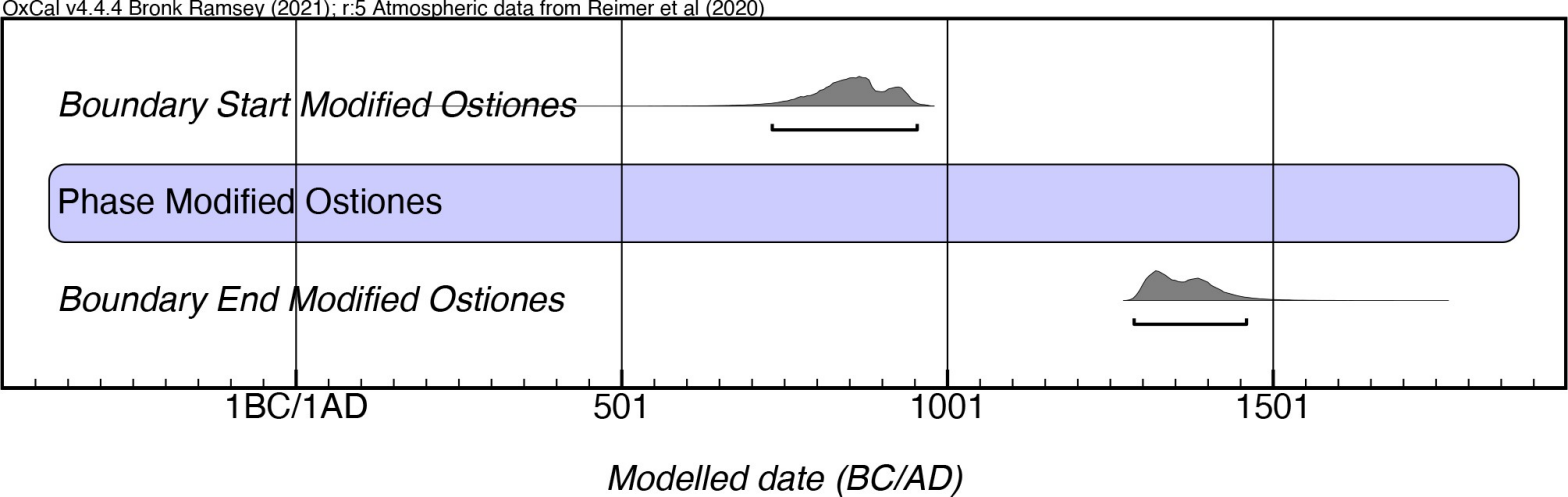
Modeled date ranges for the Modified Ostiones manifestation in Puerto Rico. Probability distributions indicate modeled 2σ (95.4%) confidence intervals for beginning and end of phase.

For the Modified Ostiones, the commonly assumed date range is between 900–1200 AD. Our model indicates a potentially earlier start date, with the early end of the modeled boundary start extending into the early 8th century mcalAD, and a later persistence, lasting as late as the 15th century mcalAD (Fig 12; S8 Table). We would note that some caution should be exercised in interpretation of these dates given the relatively small number of samples (n = 16) and sites (n = 4) involved. This caveat aside, it is noteworthy that while Modified Ostiones has a later onset than Pure Ostiones (from which it purportedly descended), it also would appear to terminate prior to the end of the period of manifestation of Pure Ostiones.

### Monserrate style

One of the most poorly defined styles in Rouse’s work is the Monserrate [see 18], which takes its name from the La Monserrate site in northeastern Puerto Rico. In general, this type of pottery is coarser than that of the Cuevas style, fired at lower temperatures, and having a more pronounced presence of tempering additives. Both painted and unpainted pots are documented, having distinct technological attributes; while painted wares are better fired, more polished, and thinner, the unpainted ones are coarser in their surface texture, have a weaker paste, and are thicker walled. Decoration consists of red slips and black smudging, modeled *adornos*, vestigial handles, and loop handles that rise above vessel rims.

**Fig 13:**
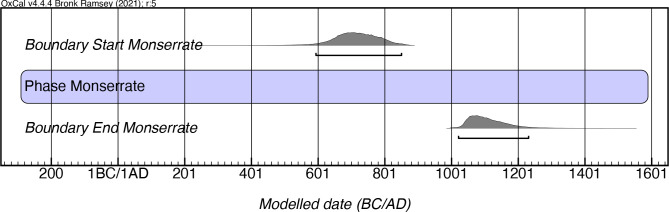
Modeled date ranges for the Monserrate style in Puerto Rico. Probability distributions indicate modeled 2σ (95.4%) confidence intervals for beginning and end of phase.

The Rousean chronology placed the Monserrate style between AD 600 and 900, although it is unclear from which site he derived this temporal approximation, as no dates associated with this style are reported in his works. Our modeling indicates a longer period of existence for the style, with early manifestations as early as mcalAD 590, and a latest date of appearance stretching until mcalAD 1240 (Fig 13; S9 Table). We would suggest employing a modicum of caution, however, given the relatively small number of samples (n = 17) included in this model. All of the dates available for this style are from the eastern part of the island, which fits with the spatial patterning for this style provided in Rouse’s model. This styleseems to be tightly linked to similar manifestations in the Virgin Islands, where it also extends to AD 1150, if not later [50].

### Santa Elena style

The Santa Elena style is predominantly comprised of boat-shaped and circular bowls that tend to have thick walls, and which feature a coarse and soft paste. The most common decorative attribute is the presence of linear incisions perpendicular to the rims, together with the use of vestigial handles. A minor proportion of pots also present the use of confined red painting. The incisions tend to be more pronounced than in other styles, and crude modeled effigies are also noted.

**Fig 14:**
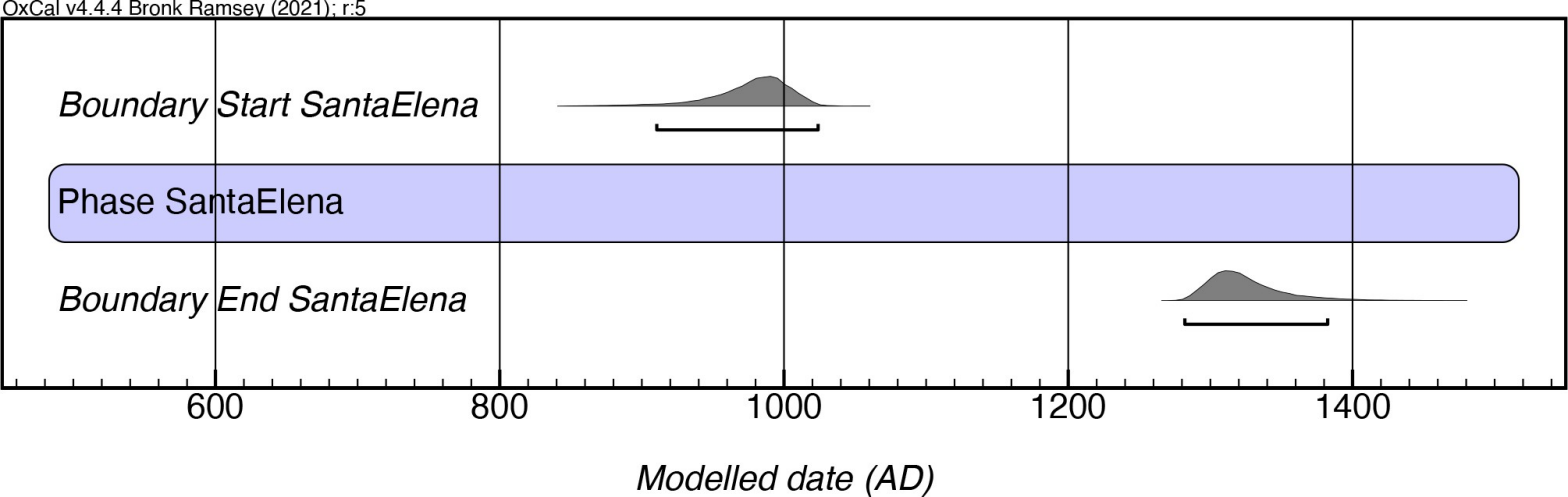
Modeled date ranges for the Santa Elena style in Puerto Rico. Probability distributions indicate modeled 2σ (95.4%) confidence intervals for beginning and end of phase.

Rouse established a chronology for this style based on two dates from the Santa Elena site. Based on the modeling of 25 dates derived from 10 sites, our results support Rouse’s assertion that the Santa Elena first appears around 900 AD (Fig 14, S10 Table). However, the results of our model indicate a longer duration (until as late as mcalAD 1390) than Rouse’s postulated end date of AD 1200. It should be noted that all the dates were obtained from contexts in the eastern half of the island, supporting the idea of the geographic emergence and distribution of this manifestation proposed by Rouse [4]. In the central northern and southern parts of the island, this style frequently appears in mixed contexts associated with Monserrate and Ostiones materials.

### Capá style

Rouse [1:350] describes Capá pottery as the “crudest in the Antilles.” It is commonly sand-tempered, with vessel surfaces that are coarse, soft, and commonly highly degraded. Vessels tend to be unrestricted, with globular forms predominating and featuring a minimal representation of boat-shaped pots. Rims tend to be rounded, tapered, and everted, and roughly shaped lugs are attached beneath the rims. Capá pottery is decorated with plastic designs, the most common of which is known as the “Capá eye”, which consists of a circular incision with a dot in its center, while also presenting appliques of zoomorphic figures.

**Fig 15:**
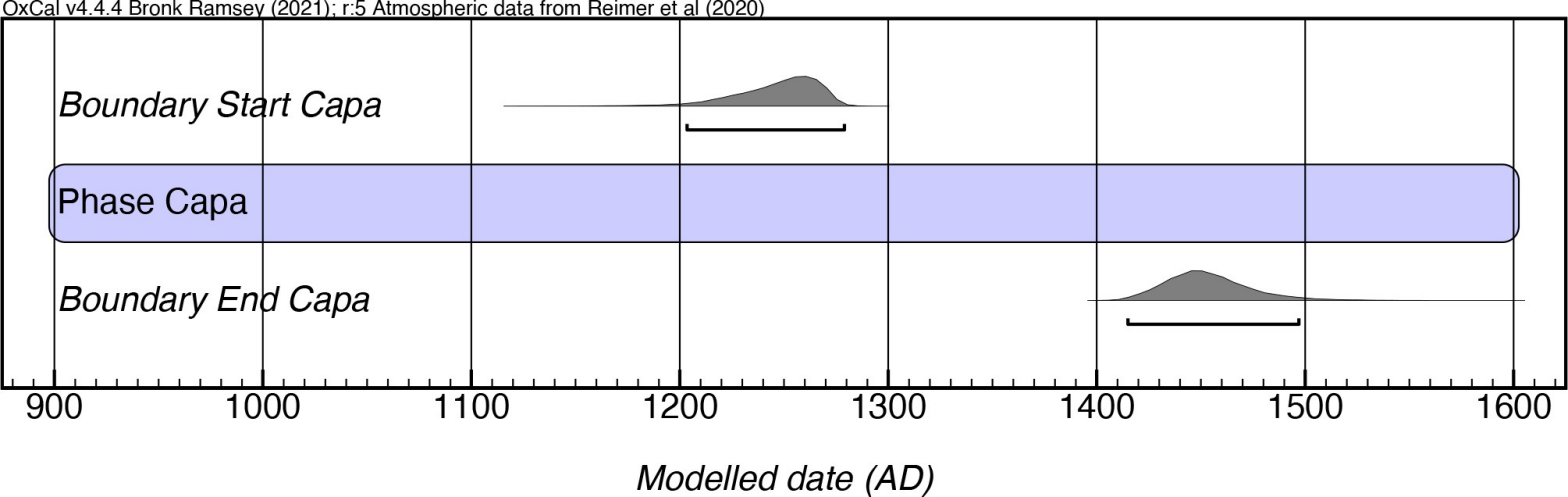
Modeled date ranges for the Capá style in Puerto Rico. Probability distributions indicate modeled 2σ (95.4%) confidence intervals for beginning and end of phase.

Rouse’s chronology of the Capá style, based on a single date from Caguana, postulated a 300-year-long phase extending from ca. 1200 to 1500 AD. Our modeling confirms this finding, with the earliest modeled boundary start date (mcalAD 1200) and latest modeled boundary end date (mcalAD 1500) aligning exactly with Rouse’s proposed temporal boundaries of the style (Fig 15; S11 Table). The earliest evidence for this style in single component contexts comes from the western (Batey del Delfín, Mayagüez) and northern (Santa Elena, Toa Baja) parts of the island, although it also appears mixed in an even earlier context in the south (Jácana).

### Esperanza style

The Esperanza style is characterized by the presence of pots that tend to be hemispherical in shape, some carinated, with a low proportion of boat-shaped vessels. Pots usually lack strap handles, with perforated lugs attached to vessel walls. Animalistic representations are noted in the appliques and adornos, with incisions being the major form of decoration, tending to consist of pairs or triads of semicircular or straight thick lines in patterns located mainly on the shoulders of the vessels and between the carinations and rims.

**Fig 16:**
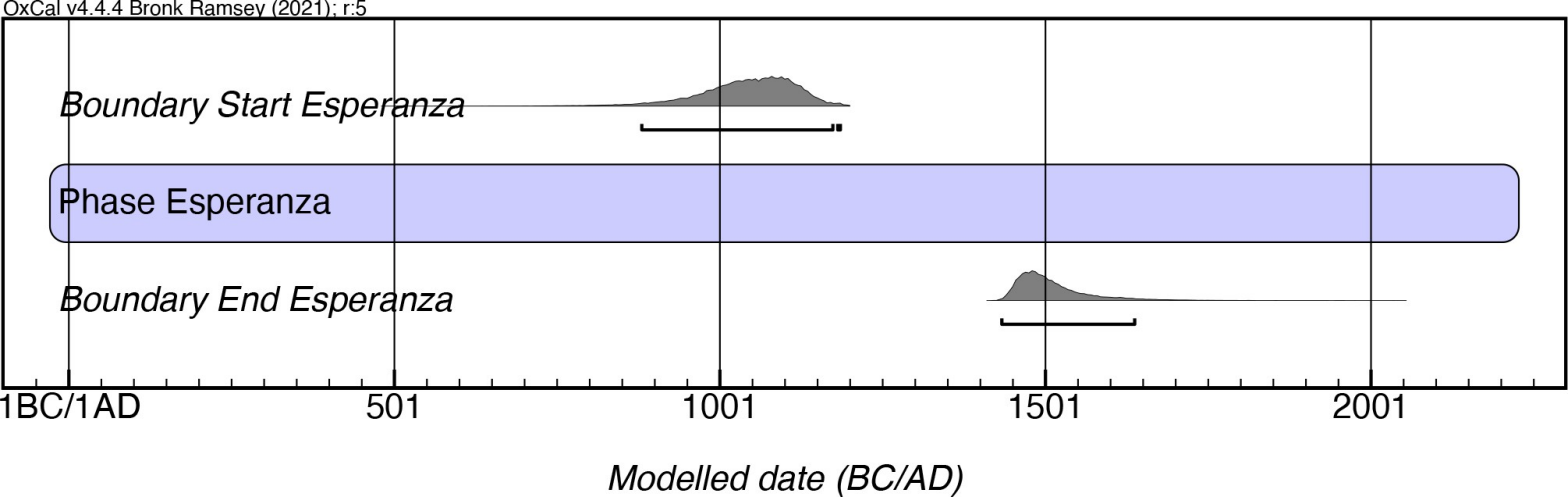
Modeled date ranges for the Esperanza style in Puerto Rico. Probability distributions indicate modeled 2σ (95.4%) confidence intervals for beginning and end of phase.

As with Capá, Esperanza was defined by Rouse as having lasted for some 300 years, from AD 1200–1500. Our model, which is hampered by the limited number of available dates (n = 11), suggests a far earlier onset (as early as mcalAD 880) and persistence into the 17^th^ century mcalAD, at the latest (Fig 16; S12 Table). However, it should be noted that the assays that predate AD 1200 all come a single site, Barrazas, in Carolina. Thus, the early range of this date requires further confirmation. This style often appears mixed with the Capá in the central part of the island, both in its north and south, in sites such as Río Cocal (Toa Baja) and Plan Bonito (Ponce) respectively.

### Bringing the models together

Moving beyond consideration of individual styles to the overall scope of Rouse’s cultural chronology, the modeling exercised detailed above permits definition of a more holistic and robust overall temporal sequence for the island of Puerto Rico.

**Fig 17:**
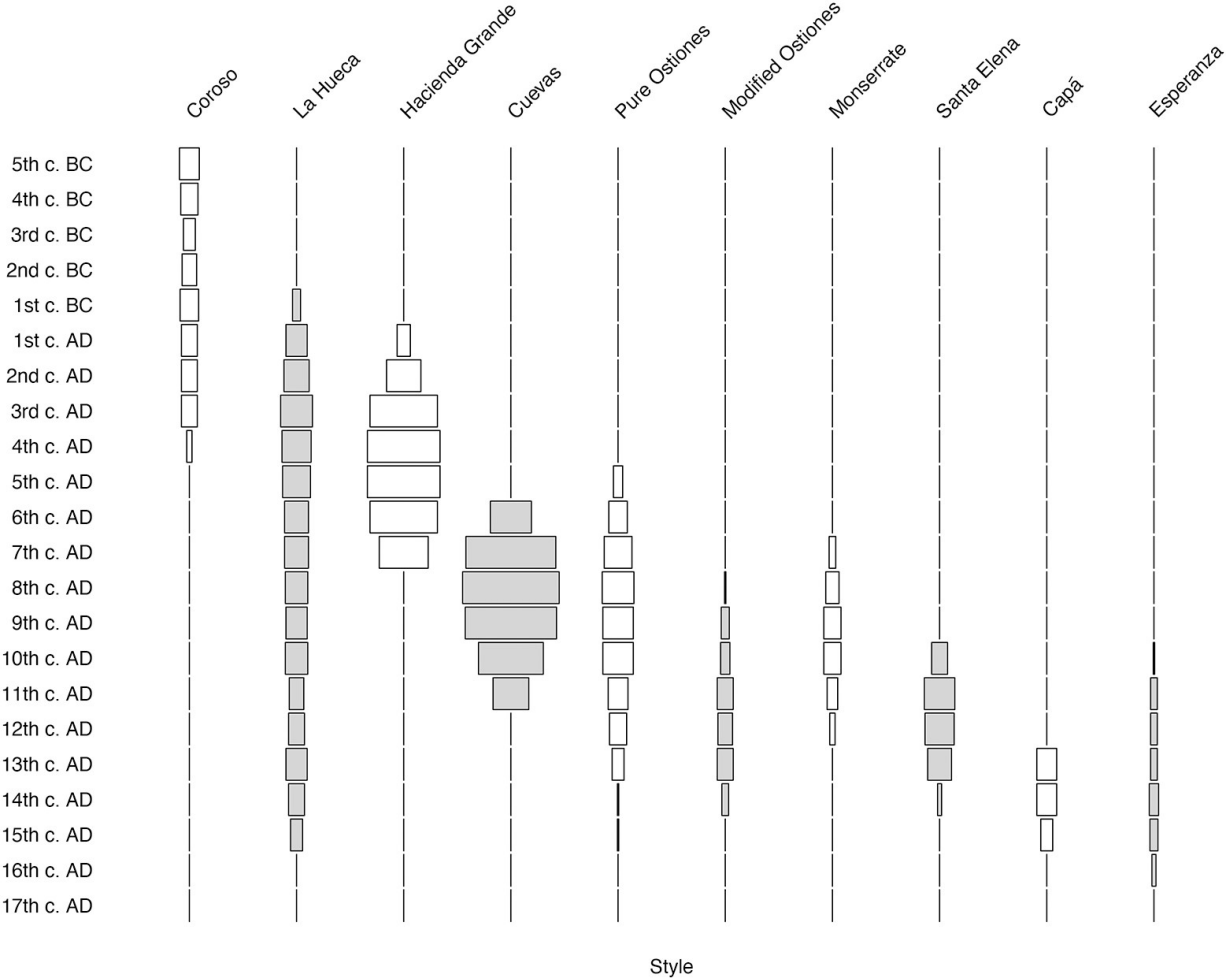
Battleship curves (count by century) of modeled and calibrated dates (mcal) for 10 Rousean styles. Note that Coroso style’s duration is artificially begun in the 5^th^ century B.C., as its prolonged duration (over 4000 years) otherwise distorts scale of figure to such a degree as to make it useless. Moreover, 100% of dates prior to 5^th^ century B.C. belong to Coroso style.

**Fig 18:**
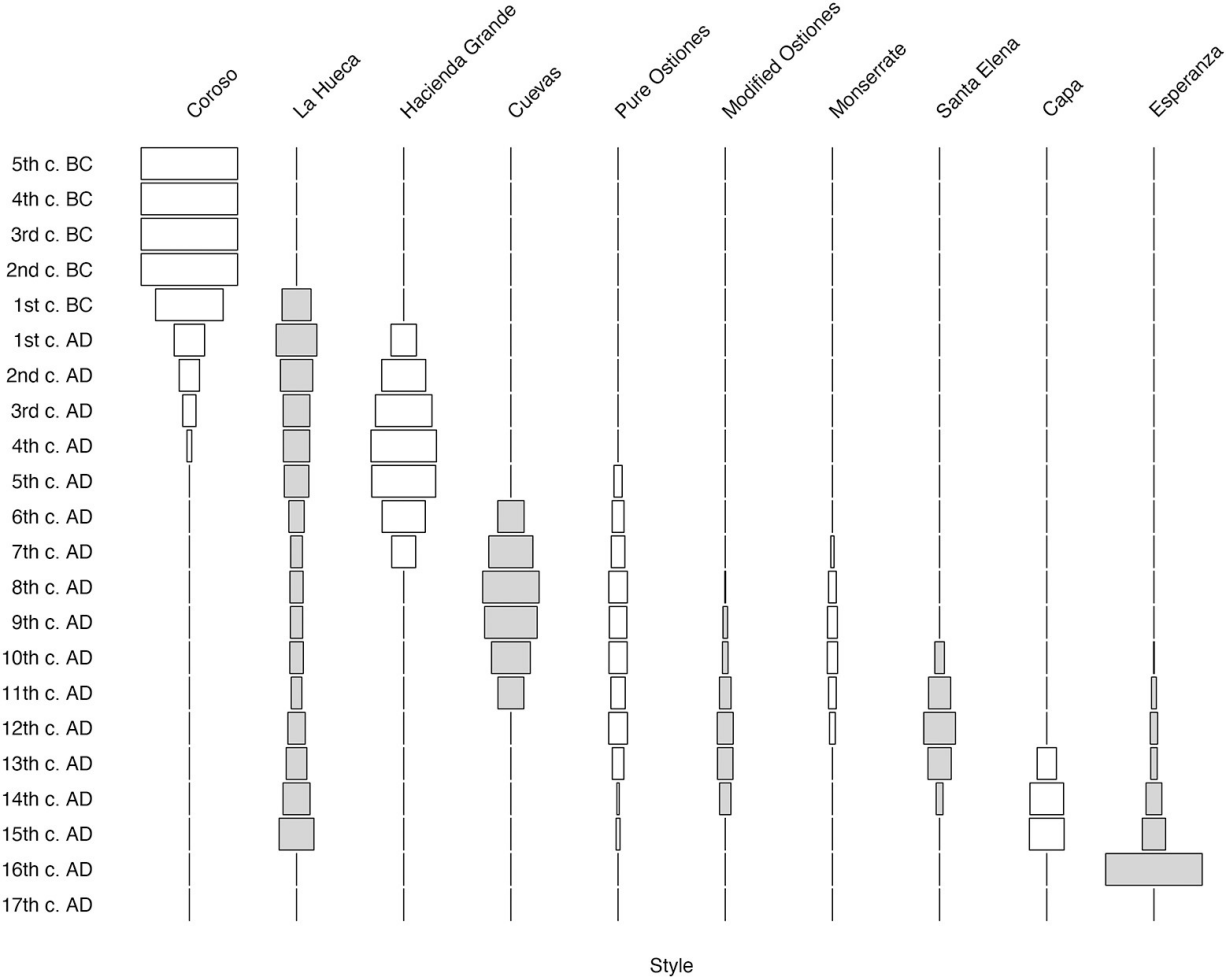
Battleship curves (percent of dates by style and century) of modeled and calibrated dates (mcal) for 10 Rousean styles. Note that Coroso style’s duration is artificially begun in the 5^th^ century B.C., as its prolonged duration (over 4000 years) otherwise distorts scale of figure to such a degree as to make it useless. Moreover, 100% of dates prior to 5^th^ century B.C. belong to Coroso style.

In Figs 17 and 18, we present two versions of the modeled chronology of the ten Rousean styles, in which we have summed the modeled and calibrated (mcal) ranges of each individual date belonging to the ten modeled styles to form battleship curves binned by century. In both instances, we begin the timeline in the 5^th^ century BC because all assays prior to that time belong to the Coroso style alone and including those nearly four millennia on the Y-axis distorts the figure horribly. In Fig 17, the count of dates whose ranges fall into each century determines the width of each century’s bar, whereas in Fig 18, it is the percentage of dates belonging to each century that determines the relative width of each style’s bar.

Irrespective of whether one considers dates by count or percentage, the juxtaposition of these various style models brings into stark relief shortcomings of the prior cultural chronology for the island. While numerous conclusions can be made from these data, we would draw particular attention to the following:

1. The prolonged (greater than five centuries) overlap of the Coroso style, on the one hand, and both early styles of the Cedrosan Saladoid (La Hueca and Hacienda Grande), on the other. Coexistence of groups of people making and using the material culture that typify these different styles directly refutes arguments of speedy replacement or assimilation of so-called “Archaic” groups at the time of arrival of the later migrants.
2. The emergence of Pure Ostionan ceramics at the height (chronologically) of the early Cedrosan Saladoid, a finding that not only renders untenable the idea that Pure Ostiones ceramics evolved from the Cuevas style, which it now is shown to predate, but greatly complicates understandings of the *in situ* cultural evolution of Ostionoid cultural traditions. Again, notions of one-for-one replacement of one style by another seems untenable in light of these findings.
3. The existence of profound cultural plurality for vast stretches of the island’s precolonial history, as for example in the 10^th^ century AD, when ceramics of as many as seven different styles (La Hueca, Cuevas, Pure Ostiones, Modified Ostiones, Monserrate, Santa Elena, and Esperanza) would appear to be contemporaneously manifested in the island. Beyond challenging fundamental Rousean assumptions, the simultaneous existence of so many distinct cultural traditions would also seem to undermine recently advanced arguments based on ancient DNA studies [51] about population bottlenecks on the island in the few centuries preceding European arrival. Populations with tiny effective sizes would not be able to maintain independent modes of material culture production for centuries-long periods of time.
4. The relatively rapid cessation of the production of identifiable aboriginal ceramics by or in the 16^th^ century AD, can be taken as an indication, perhaps, of the rapidity and intensity of the cultural consequences of colonization, which saw the extirpation and transformation of many archaeologically visible Indigenous modes of cultural production in a relatively short period of time. Unfortunately, at present it is difficult to present a more definitive temporal bounding of the latest phase of precolonial occupation of the island as its related contexts are indeed the ones over which the impacts of agriculture, construction, and other activities that have had adverse effects on the archaeological record have been registered. More research is definitely needed to address potential contexts of Indigenous survival during colonial times, as has been documented historically and anthropologically [e.g. 52–54], as well as the spaces of re-accommodation of these Indigenous societies after the colonization of the island.

As indicated above, however, these are but a few of the potential implications that can be drawn from the simultaneous consideration of the style models presented here.

**Fig 19:**
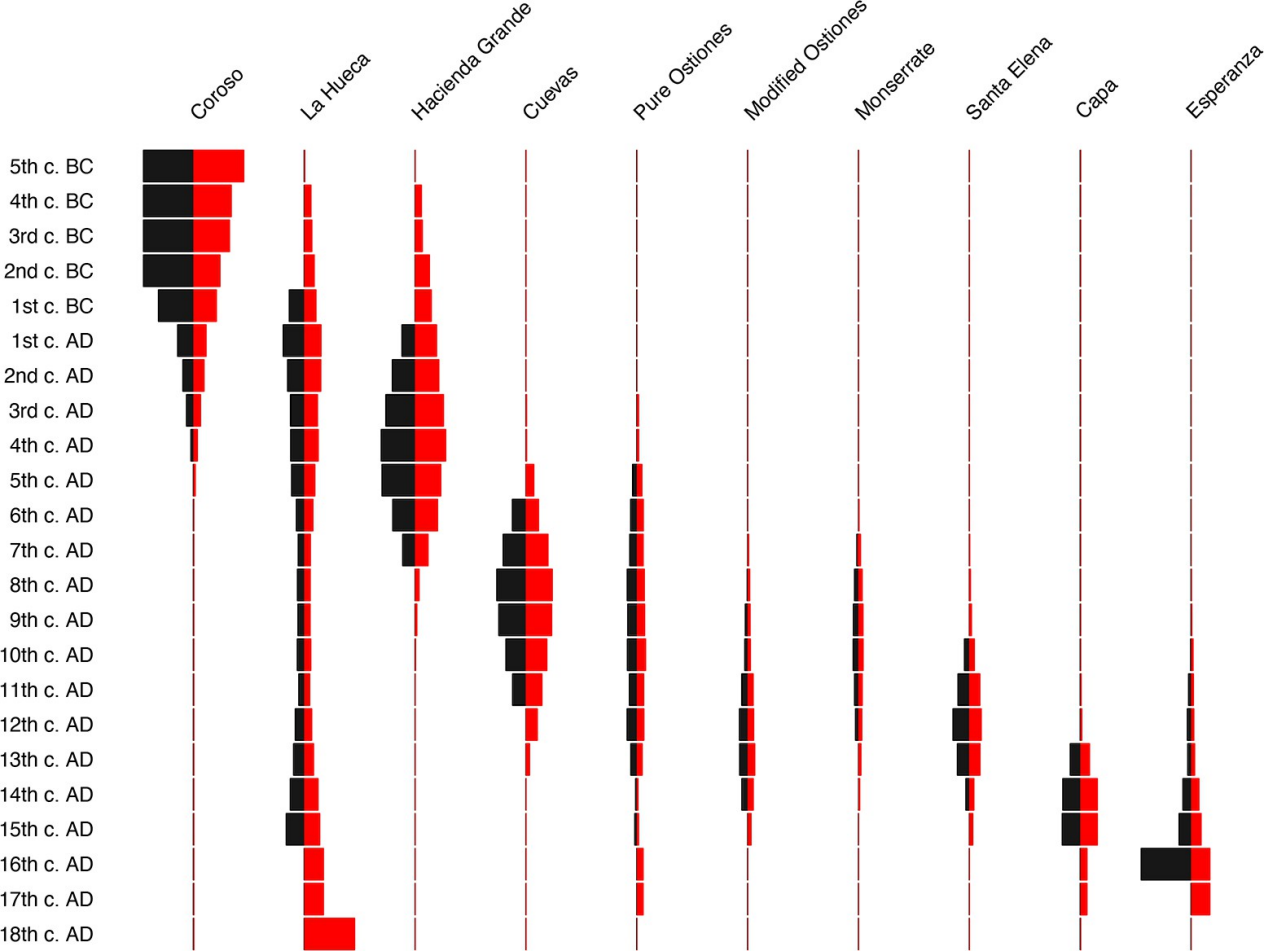
Battleship curves (percent by century) of modeled and calibrated dates for 10 Rousean styles. Modeled and calibrated (mcal) dates presented in black, calibrated but unmodeled dates in red. Note that Coroso style’s duration is artificially begun in the 5^th^ century B.C., as its prolonged duration (over 4000 years) otherwise distorts scale of figure to such a degree as to make it useless. Moreover, 100% of dates prior to 5^th^ century B.C. belong to Coroso style.

To make clear the effects that modeling of dates by style had on the overall temporal distribution of each style (and thus the relationships among the styles), we also present in Fig 19 a comparison of the modeled and calibrated dates. As in Fig 18, the width of the bars is determined by the percentage of each date by century, but whereas the modeled and calibrated (mcal) dates are presented in black, the red bars are the calibrated, but unmodeled, dates for each stylistic grouping.

## Discussion

As is made evident by the results presented above, a revision of the cultural chronology for the island of Puerto Rico is long overdue. Given that many of the modeled date ranges for the styles defined by Rouse do not match with previous estimations, these findings markedly alter some of the most fundamental notions held by many about the sequence of the cultures that migrated to and developed in the island, as well as some of the interpretations that have been, and continue to be, made using Rouse’s model as a basis.

Starting with the humanization of the island by its earliest settlers, the evidence points to a time of onset (between 4300–4050 mcal BC, or 6250–6000 mcal BP) at least three millennia earlier than suggested in Rouse’s model and more than fifteen hundred years prior to the latest published estimations thereof (4655–4305 mcal BP from [29]). These dates are older than the colonization estimates for the Greater and Lesser Antilles modelled by Napolitano et al. [29], for Cuba (5360–4675 mcal BP) and St. Martin (5275–4940 mcal BP) respectively and are exceeded only by the modeled colonization of Trinidad (8420–7285 mcal BP). This underlines the need to consider the possibility of direct trans-Caribbean crossings from the surrounding continents into Puerto Rico and the northern Lesser Antilles, as had been previously suggested based on the introduction of particular phytocultural traditions into these islands whose distributions are mostly confined between northwestern Venezuela and Panama while being absent in the rest of the Greater and Lesser Antilles [43, 55]. As previously noted, the modeled evidence for Coroso also indicates a much later persistence of these first groups (mcalAD 130–430), which contrasts starkly with their quick disappearance suggested in Rouse’s model. The types of interaction that should have taken place by these early settlers and the later newcomers are definitely a matter that should be further evaluated in future studies.

The results presented here also indicate a later arrival of groups associated with the Cedrosan Saladoid subseries, as represented by the Hacienda Grande style. This is one of the instances in which we think that the findings of the present study might be drastically altered when additional dates are generated, given the presence of earlier dates that were obtained from reliable archaeological contexts, but which were eliminated due to their low agreement values within our model. Until similar modeling is conducted on islands in the Lesser Antilles for each of their respective cultural complexes, it will be difficult to parse whether there were direct movements from northern South America into the northern Lesser Antilles and Puerto Rico, initially bypassing the southern Lesser Antilles (“The Southward Route Hypothesis”), as suggested by Fitzpatrick [56], or if the migration via the Lesser Antilles, as proposed by Rouse [4], still holds.

In the case of the La Hueca style, the results of this study still leave the main chronological questions about the style unresolved, particularly when trying to make sense of its markedly long duration. Although the dates seem to show a multimodal distribution, we have yet to arrive at a satisfactory explanation for the observed chronological pattern for this style. Hopefully, presentation of this finding and all data used to build it will prompt further research in sites associated with this style in order to foment better understandings of its temporality and its relationship with other cultural manifestations in the island.

Matters become even more complicated after calAD 500, when new styles emerge and there is a profound degree of overlap among and between the different stylistic manifestations of the island, giving rise to an impression of a heavily variegated cultural landscape. The emergent cultural plurality may well have been the result of the different types of interactions that took place between the first inhabitants of the island and those peoples associated with the production of the Hacienda Grande and La Hueca styles. Some issues become evident from the sequencing of the dates of different styles at this time, such as the proposed “genetic” derivation of the Ostiones style from the Cuevas style assumed in Rouse’s model, given that the former seems to be earlier than the latter. What seems clear is that different processes of ethnogenesis took place as a result of the interactions that occurred within the island, and likely also as a result of the ongoing contacts that were taking place with peoples from other islands and surrounding continental mainlands.

Another sequela of the modeling effort undertaken here is that, as expected, the duration of the styles seems to vary geographically. For instance, in the case of the Cuevas, the individual dates for the style extend at least three centuries later in eastern Puerto Rico than in the western part of the island. This is also the case for the Pure Ostiones, which extends much later in the mountainous interior than it does on the coast. Clearly, further spatial modeling of the dates from a microregional standpoint is needed in order to characterize and bound this spatial variation in the temporalities for each style across the island.

A final point that is made evident from this exercise in modeling is that the chronological origins of the various styles do not necessarily coincide with the onset of other social changes, as has been commonly understood [e.g. 57–61]. For instance, the broadly shared assumption that the marked social changes that were associated with the onset of the Ostiones and Monserrate styles (complex tribes) or the Capá and Esperanza styles (chiefdoms) should be clearly reconsidered. Based on the chronological evidence, it seems likely that that the noted stylistic changes in pottery production might have responded to other dynamics related to the negotiation of identity, belief systems, ecological factors, and other structural or superstructural phenomena. Because of this lack of a temporal concomitancy between styles and other social changes, together with the multicultural landscape that seems to have existed in the island throughout much of its history, other forms of periodization for its precolonial history that do not prioritize the style as the main indicator should be considered, as has been previously proposed [26, 62, 63].

### Concluding remarks

The results of the modeling of the inventory of dates presented here provide a basis for revisiting the culture-historical sequence of Puerto Rico and begin to offer a means for addressing some of the main chronological issues that we have yet to resolve in our quest to generate better insights into the diverse cultures that inhabited the island in precolonial times. Although this work perhaps raises more questions than it answers, we consider that it provides a baseline that might paint a more realistic and probabilistic picture of the complicated cultural and chronological landscape that existed in the island through time. Throughout the island’s ancient history, the coexistence of diverse cultural manifestations seems to have been the rule, rather than the exception, in sharp contrast to the assumption of the sequential cultural replacement of one style by the next ingrained in Rouse’s model. In tandem, the modeling of this inventory of dates makes evident that it is no longer tenable to continue relying on the temporal constructions established almost a half-century ago based on dates that were obtained without adherence to adequate standards of collection, submission, and reporting, were not properly calibrated, and would otherwise be inadmissible knowing what we know now about the process of radiocarbon dating.

Unfortunately, some of these problems that were noted in the original dates that were generated by Rouse and Alegría in the 1960s are still persistent in the archaeology of Puerto Rico and the rest of the Antilles in general, as noted by Fitzpatrick [28] and Napolitano et al. [29]. This underlines the need for scholars to follow the accepted protocols for obtaining radiocarbon dates and for providing the necessary information in their reporting. The results of our hygiene and chronological fitness protocols, as well as of the elimination process involved in the Bayesian analysis of the dates, obviously led us to omit around 40% of the assays obtained from the island. Thus, the modeled date ranges that were established for each style in this study should be considered as providing a conservative reflection of the potential temporalities of the styles analyzed. These chronologies shall be better refined as new dates become available, specially from some of the more than 30 municipalities that remain undated, dates which might drastically alter the absolute temporal placement of the diverse cultural manifestations that have been documented in the island.

The evidence also shows that there is marked variation in the temporal resolution obtained for the different styles that have been defined thus far (i.e., some last for only a few centuries, some for millennia). If nothing else, this finding makes clear the importance of dating sites and contexts using the available absolute means, rather than simply assuming temporality based on stylistic affiliations. Most crucially, perhaps, the prolonged duration of some of these styles limit the interpretations that can be made using them as a basis; what does it mean, in human/anthropological terms, to say that a given social change happened in a certain millennium? To be clear, the data and interpretations presented here are not proposed as a new dogma, but rather a reflection of the temporal dimensions of the amassed radiocarbon data on the Rousean derived system (which has the various methodological shortcoming noted above). Hopefully, this work shows the chronological challenges that we still face on the island and serves as an impetus for generating new data to better characterize the temporal dimension in the archaeology of Puerto Rico, and beyond.

## Supporting information

S1 Table Database of n = 1,008 radiocarbon dates from Puerto Rico, including for each sample (as available): Contextual data, original dating details and results, calibration parameters used, calibrated and modeled/calibrated date ranges, bibliographic information, and chronometric hygiene results. (XLSX)

S2 Table Full results of final OxCal modeling of Coroso style dates. (XLSX)

S3 Table Full results of final OxCal modeling of Hacienda Grande style dates. (XLSX)

S4 Table Full results of final OxCal modeling of La Hueca style dates. (XLSX)

S5 Table Full results of final OxCal modeling of Cuevas style dates. (XLSX)

S6 Table Full results of final OxCal modeling of All Ostiones style dates. (XLSX)

S7 Table Full results of final OxCal modeling of Pure Ostiones style dates. (XLSX)

S8 Table Full results of final OxCal modeling of Modified Ostiones style dates. (XLSX)

S9 Table Full results of final OxCal modeling of Monserrate style dates. (XLSX)

S10 Table Full results of final OxCal modeling of Santa Elena style dates. (XLSX)

S11 Table Full results of final OxCal modeling of Capá style dates. (XLSX)

S12 Table Full results of final OxCal modeling of Esperanza style dates. (XLSX)

S1 File Bibliographic data for all radiocarbon dates presented in S1 Table. (DOCX)

S2 File 1) Details of calculation of local ΔR values for north and south coasts of Puerto Rico, and 2) method employed for calculation of marine carbon contribution used in calibration of bone dates. (DOCX)

S1 Fig Pottery of the Hacienda Grande style (Collection of the Centro de Investigaciones Arqueológicas, Universidad de Puerto Rico, Recinto de Río Piedras). (TIF)

S2 Fig Hacienda Grande style adornos. (Collection of the Centro de Investigaciones Arqueológicas, Universidad de Puerto Rico, Recinto de Río Piedras).
(TIF)

S3 Fig Pottery of the La Hueca style. (Collection of the Centro de Investigaciones Arqueológicas, Universidad de Puerto Rico, Recinto de Río Piedras).
(TIF)

S4 Fig La Hueca style adornos (Collection of the Centro de Investigaciones Arqueológicas, Universidad de Puerto Rico, Recinto de Río Piedras). (TIF)

S5 Fig Pottery of the Cuevas style originally presented by Rouse [1:443] in his definition of the styles for Puerto Rico. Note that artifacts ANT.85033, ANT.32089, and ANT.32433, which were included in Rouse’s original image of pottery of the Cuevas style, were excluded from this figure as they are of the Hacienda Grande style, which he had not yet defined at the time of his publication (adapted from photo courtesy of Madeliz Gutierrez Ortiz) (a. ANT.63975; b. ANT.36513; c. ANT.36513; d. ANT.85218; e. ANT.63890; f. ANT.85584; g. ANT.32233; h. ANT.98061; i. ANT.31533; Collection of the Yale Peabody Museum, Division of Anthropology).
(TIF)

S6 Fig Pottery of the Cuevas style (a, b, d, e, f, g are from Collection of the Universidad Ana G. Méndez, Recinto de Gurabo; c is from the Centro de Investigaciones Arqueológicas, Universidad de Puerto Rico, Recinto de Río Piedras).
(TIF)

S7 Fig Pottery of the Ostiones style originally presented by Rouse [1:445] in his definition of the ceramic styles of Puerto Rico. (adapted from photo courtesy of Madeliz Gutierrez Ortiz) (a. ANT.98019; b. ANT.97268; c. ANT.77752; d. ANT.83152; e. ANT.81368; f. ANT.66136; g. ANT.66407; h. ANT.82213; i. ANT.81348; j. ANT.81074; k. ANT.80989; l. ANT.81132; m. ANT.34563; n. ANT.97722; o. ANT.80231; Collection of the Yale Peabody Museum, Division of Anthropology).
(TIF)

S8 Fig Pottery of the Early (i.e. Pure) Ostiones style (Collection of the Laboratorio de Arqueología, Departamento de Sociología y Antropología, Universidad de Puerto Rico, Recinto de Río Piedras).
(TIF)

S9 Fig Pottery of the Late (i.e. Modified) Ostiones style (a is from the Collection of the Centro de Investigaciones Arqueológicas, Universidad de Puerto Rico, Recinto de Río Piedras; b, c, d and f are from the Collection of the Laboratorio de Arqueología, Departamento de Sociología y Antropología, Universidad de Puerto Rico, Recinto de Río Piedras; e is from the Collection of the Laboratorio de Arqueología, Universidad de Puerto Rico, Recinto de Utuado).
(TIF)

S10 Fig Pottery of the Monserrate style. Note that artifact a (ANT.35452; Collection of the Yale Peabody Museum, Division of Anthropology), which Rouse [1:447] had originally included in his original plate within the Santa Elena style, is presented in this image as it is of the Monserrate style that he had not yet defined at the time of his publication (b-e, g. Collection of the Laboratorio de Arqueología, Universidad de Puerto Rico, Recinto de Río Piedras; f. Collection of the Universidad Ana G. Méndez, Recinto de Gurabo).
(TIF)

S11 Fig Pottery of the Monserrate style (Collection of the Universidad Ana G. Méndez, Recinto de Gurabo).
(TIF)

S12 Fig Pottery of the Santa Elena style originally presented by Rouse [1:447] in his definition of the styles for Puerto Rico. Note that artifacts ANT.35452 and ANT.96501 are excluded from this image as they are from the Monserrate style, which he had not yet defined at the time of his publication (adapted from photo courtesy of Madeliz Gutierrez Ortiz) (a. ANT.88358; b. ANT.88219; c. ANT.70050; d. ANT.88167; e. ANT.66328; f. ANT.35903; g. ANT.33896; Collection of the Yale Peabody Museum, Division of Anthropology).
(TIF)

S13 Fig Pottery of the Santa Elena style (Collection of the Universidad Ana G. Méndez, Recinto de Gurabo).
(TIF)

S14 Fig Pottery of the Capá style originally presented by Rouse [1:451] in his definition of the styles for Puerto Rico. (adapted from photo courtesy of Madeliz Gutierrez Ortiz) (a. ANT.89287; b. ANT.92883; c. ANT.74186; d. ANT.87779; e. ANT.93052; f. ANT.87870; g. ANT.66324; h. ANT.74914; i. ANT.96943; j. ANT.91903; k. ANT.87860; l. ANT.75631; m. ANT.87355; n. ANT.88455; o. ANT.66157; p. ANT.89234; q. ANT.87988; Collection of the Yale Peabody Museum, Division of Anthropology).
(TIF)

S15 Fig Pottery of the Capá style. (a-d, f. Laboratorio de Arqueología, Departamento de Sociología y Antropología, Universidad de Puerto Rico, Recinto de Río Piedras. Note that object e [ANT.91615; Yale Peabody Museum, Division of Anthropology], which was originally included by Rouse [1:449] as part of the Boca Chica style, is hereby considered to be of the Capá style.
(TIF)

S16 Fig Pottery of the Capá style (a-f. Laboratorio de Arqueología, Universidad de Puerto Rico, Recinto de Río Piedras; g, modified from Rodríguez López [64]).
(TIF)

S17 Fig Pottery of the Esperanza style originally presented by Rouse [1:453] in his definition of the styles for Puerto Rico. (adapted from photo courtesy of Madeliz Gutierrez Ortiz) (a. ANT.90898; b. ANT.90776; c. ANT.90955; d. ANT.90774; e. ANT.67625; f. ANT.93152; g. ANT.90713; h. ANT.66072; i. ANT.67148; j. ANT.90785; k. ANT.67949; l. ANT.67065; m. ANT.90680; n. ANT.90810; o. ANT.90809; p. ANT.90970; Collection of the Yale Peabody Museum, Division of Anthropology).
(TIF)

S18 Fig Pottery of the Boca Chica style originally presented by Rouse [1:453] in his definition of the styles for Puerto Rico. Note that we excluded object ANT.91615 from this figure as we consider it to be of the Capá style (adapted from photo courtesy of Madeliz Gutierrez Ortiz) (a. ANT.91615; b. ANT.69895; c. ANT.91340; d. ANT.91728; e. ANT.91762; f. ANT.91376; g. ANT.91746; h. ANT.91344; i. ANT.70013; j. ANT.699951; k. ANT.91482; l. ANT.91281; Collection of the Yale Peabody Museum, Division of Anthropology).
(TIF)
