# Supplementary material for: Revision of the cultural chronology of precolonial Puerto Rico: A Bayesian approach
Source: PLoS One. 2023 Feb 22;18(2):e0282052. doi: 10.1371/journal.pone.0282052 (PMC9946257; doi:10.1371/journal.pone.0282052)
Supplement: S1 File — (DOCX) [file pone.0282052.s013.docx]

1. González Colón J. Evaluación arqueológica (fase de mitigación), Sitio de Aguilita, Proyecto Urb. Santa Rita, Juana Díaz. Report on file at the Consejo de Arqueología Terrestre, San Juan: 1997.

2. Vega J. Resultado de laboratorio de radiocarbono, sitio arqueológico de Angostura, Barceloneta, Puerto Rico. Report on file at the Consejo de Arqueología Terrestre de Puerto Rico, San Juan: 2002.

3. Rivera-Collazo IC. Between Land and Sea in Puerto Rico: Coastal Landscapes and Human Occupations in the Mid-Holocene Caribbean: University College London; 2011.

4. Ayes Suárez CM. Evaluación arqueológica tipo Fase 2, Angostura, Florida Afuera, Barceloneta, Puerto Rico. Report on file at the Consejo para la Protección del Patrimonio Arqueológico Terrestre de Puerto Rico, 1988.

5. Meléndez MJ. Mitigación arqueológica del yacimiento de Barrazas, Carolina (Fase III-Etapa III), Proyecto Centro de Servicios Múltiples de Barrazas. Report on file at the Consejo de Arqueología Terrestre de Puerto Rico, San Juan: 1996.

6. Rivera Fontán V, Silva Pagán D. Informe técnico Proyecto Arqueológico del Barrio Quemado, Mayagüez Sitio Batey Delfín del Yagüez. Report on file at the Consejo de Arqueología Terrestre de Puerto Rico, San Juan: 2005.

7. Príncipe Jácome AM. Investigación Arqueológica de Fase II Proyecto Bella Vista Retirement Center, Mayagüez, Puerto Rico. Report on file at the Consejo de Arqueología Terrestre de Puerto Rico, San Juan: 1999.

8. Rouse I, Alegría RE. Radiocarbon Dates from the West Indies. Revista/Review Interamericana. 1979;8(3):495-9.

9. Rodríguez López M. Yacimiento Caguitas, Caguas, Puerto Rico (ICP/CAT-CS-02-26-04). Report on file at the Consejo de Arqueología Terrestre de Puerto Rico, San Juan: 2002.

10. Pérez C. Informe Preliminar, Caguitas Cs-2. Report on file at the Consejo para la Protección del Patrimonio Arqueológico Terrestre de Puerto Rico, San Juan: 1999.

11. Fernandes DM, Sirak KA, Ringbauer H, Sedig J, Rohland N, Cheronet O, et al. A genetic history of the pre-contact Caribbean. Nature. 2021;590(7844):103-10.

12. Figueredo A. The Vieques Archaeological Project. Journal of the Virgin Islands Archaeological Society. 1975;2:20-4.

13. Cooper J, Samson AVM, Nieves MA, Lace MJ, Caamaño-Dones J, Cartwright CR, et al. The Mona Chronicle: the archaeology of early religious encounter in the New World. Antiquity. 2016;90:1054-71.

14. Samson AV, Wrapson LJ, Cartwright CR, Sahy D, Stacey RJ, Cooper J. Artists before Columbus: A multi-method characterization of the materials and practices of Caribbean cave art. Journal of Archaeological Science. 2017;88:24-36.

15. Ostapkowicz J, Ramsey CB, Brock F, Higham T, Wiedenhoeft AC, Ribechini E, et al. Chronologies in wood and resin: AMS 14C dating of pre-Hispanic Caribbean wood sculpture. Journal of Archaeological Science. 2012;39(7):2238-51.

16. Stuiver M. Yale Natural Radiocarbon Measurements IX. Radiocarbon. 1969;2(2):545-68.

17. Veloz Maggiolo M, González Colón J, Maíz E, Questell E. Cayo Cofresí, un Sitio Precerámico de Puerto Rico. Santo Domingo, Dominican Republic: Ediciones de Taller; 1975.

18. Carlson LA, Torres JM. Phase III Data Recovery Investigations at Three Prehistoric Archaeological Sites (CE-11, CE-32, and CE-33), Municipality of Ceiba, Naval Activity Puerto Rico. Volume 1: Final Report. Report on file at the Puerto Rico State Historic Preservation Office, San Juan: Southeastern Archaeological Research, Inc., 2011.

19. Carlson LA, Altes C. Site CE-34 Data Recovery, Punta Medio Mundo, Naval Activity Puerto Rico. Report on file at the Puerto Rico State Historic Preservation Office, San Juan: 2015.

20. Rodríguez López M. Prehistoria de Collores. San Juan, Puerto Rico: Centro de Estudios Avanzados de Puerto Rico y el Caribe; 1983.

21. Alvarado Zayas PA. Estudios adicionales, PR-53, Guayama-Salinas. Report on file at the Consejo de Arqueología Terrestre, San Juan: 1992.

22. Rouse I, Alegría RE. Excavations at María de la Cruz Cave and Hacienda Grande Village Site, Loíza, Puerto Rico. New Haven 1990.

23. Pestle WJ, Laguer-Díaz C, Rodríguez Rámos R. Final Report on March 2018 Archaeological Fieldwork Conducted in Cabo Rojo National Wildlife Refuge. Work Performed under Terms of ARPA Permit CRJNWR121517 and Special Use Permit 2018-05. Report on file with U.S. Fish and Wildlife Service, Southeast Region: 2018.

24. Staudigel PT, Swart PK, Pourmand A, Laguer-Díaz CA, Pestle WJ. Boiled or roasted? Bivalve cooking methods of early Puerto Ricans elucidated using clumped isotopes. Science Advances. 2019;5(11):eaaw5447.

25. Dávila Dávila O. Arqueología de la Isla de Mona. San Juan, Puerto Rico: Editorial Instituto de Cultura Puertorriqueña; 2003.

26. Rodríguez Ramos R, Acosta Á, Pérez Reyes R. Una aproximación a la temporalidad absoluta del arte rupestre pictográfico de Puerto Rico. Ciencia y Sociedad. 2021;46(3):7-27.

27. Rodríguez Ramos R. La temporalidad absoluta del arte rupestre pictográfico en Puerto Rico. Report on file at the Puerto Rico State Historic Preservation Office, San Juan: 2017.

28. Turvey S, Oliver J, Narganes Storde Y, Rye P. Late Holocene extinction of Puerto Rican native land mammals. Biology Letters. 2007;3(2):193-6.

29. Oliver JR, Narganes Storde YM, editors. The zooarcheological remains from Juan Miguel Cave and Finca de Doña Rosa, Barrio Caguana, Puerto Rico. Ritual edibles or Quotidian Meals? Proceedings of the XXth International Congress for Caribbean Archaeology; 2005; Santo Domingo, Republica Dominicana: Museo del Hombre Dominicano and Fundación García-Arévalo.

30. Oliver JR, Rivera Fontán JA, Newsom LA. Proyecto Arqueológico Utuado-Caguana. Breve Resumen de las Actividades de Investigaciones y Análisis. Report on file at the Consejo para la Protección del Patrimonio Arqueológico Terrestre de Puerto Rico, San Juan: 2001.

31. Oliver JR, Rivera-Collazo IC, editors. A reassessment of María de la Cruz Cave site, Puerto Rico: The 2012 excavations. Proceedings of the XXV International Congress of the Association of Caribbean Archaeology 2015; San Juan, Puerto Rico: Instituto de Cultura Puertorriqueña, Centro de Estudios Avanzados de Puerto Rico y el Caribe and the University of Puerto Rico.

32. Frank EF. A Radiocarbon Date of 380±60 BP for a Taino Site, Cueva Negra, Isla de Mona. Journal of Cave and Karst Studies. 1998;60(2):101-2.

33. Sanders SL, Saint Onge E, Goodwin RC, Davis DD, Davenport C. Archaeological Survey and Evaluation of Selected Sites at NSGA Sabana Seca, Sabana Seca, Puerto Rico. Report on file at the Puerto Rico State Historic Preservation Office, San Juan: 2001.

34. Martínez Torres R. El Yacimiento Arcaico de "La Tembladera": Primer tratado de arqueología nativa boricua. Morovis, Puerto Rico. Camuy, Puerto Rico: Ediciones Much Ma' Ho'l; 2018.

35. Rodríguez Ramos R. La ocupación temprana del Interior montañoso de Puerto Rico: Los casos de Cueva Ventana y Salto Arriba. Report on file at the Puerto Rico State Historic Preservation Office, San Juan: 2014.

36. Rodríguez López M. Late Ceramic Age Diversity in Eastern Puerto Rico. Paper presented at the 57th Annual Meeting of the Society for American Archaeology; Pittsburgh, Pennsylvania 1992.

37. Maiz EJ. El sitio arqueológico Hernández Colón: Actividades subsistenciales de los antiguos habitantes del Valle del Río Cerrillos, Bucana, Ponce, Puerto Rico: Centro de Estudios Avanzados de Puerto Rico y el Caribe, San Juan.; 2002.

38. Robinson LS, Lundberg ER, Walker JB. Archaeological Data Recovery at El Bronce, Puerto Rico, Final Report, Phase 2. Report on file at the Puerto Rico State Historic Preservation Office, San Juan: 1985.

39. Rodríguez López M, Rivera V. Archaeological Data Recovery Program at Playa Blanca 5 Site, Roosevelt Roads, Ceiba, Puerto Rico. Report on file at the Puerto Rico State Historic Preservation Office, San Juan: 1989.

40. Newsom LA. Native West Indian Plant Use. Gainesville: University of Florida; 1993.

41. Weaver GG, Garrow PH, Oliver JR. Phase II Archaeological Data Recovery at PO-38, El Parking Site, Barrio Maraguez, Ponce, Puerto Rico. Report on file at the State Historic Preservation Office, San Juan, 1992.

42. Narganes Storde YM. Nueva cronología de varios sitios de Puerto Rico. Paper presented at XXI Congreso Internacional de Arqueología del Caribe; Trinidad.2005.

43. Solís Magaña C, Rodríguez López M. Phase II Archaeological Evaluation Site NCS-1 (Finca Valencia), Municipality of Arecibo, Puerto Rico. Report on file at the Puerto Rico State Historic Preservation Office, San Juan: 1999.

44. González Colón J, Walker J. Evaluación arqueológica (Fase de Mitigación), Sitio Florida 2, San Lorenzo. Report on file at the Consejo de Arqueología Terrestre, San Juan: 2004.

45. Morsink J. Río de La Plata Flood Damage Reduction Project, Phase II National Register of Historic Places (NRHP) Evaluation of the Guaraguao Site (DDO100042), Río de La Plata, Dorado, Puerto Rico. Report on file at the Puerto Rico State Historic Preservation Office, San Juan: 2021.

46. Roe PG, editor A Preliminary Report on the 1980 and 1982 Field Seasons at Hacienda Grande (12PSj 7-5): Overview of Site History, Mapping, and Excavations. Proceedings of the 10th International Congress for Caribbean Archaeology; 1985.

47. Vélez J. Proyecto Hacienda de la Baume, Evaluación Arqueológica Fase II. Report on file at the Consejo de Arqueología Terrestre, San Juan: 2005.

48. Krause RA. Coffee, Sugar and Baked Clay: From Prehistory to History in Puerto Rico’s Cerrillo’s River Valley. Submitted to the U.S. Army Corps of Engineers, Jacksonville District. Report on file at the Puerto Rico State Historic Preservation Office, San Juan, Puerto Rico: 1990.

49. Ayes Suárez CM, Dávila Dávila O. Angostura: Un campamento arcaico temprano del valle del Manatuabón Bo. Florida Afuera, Barceloneta, Puerto Rico. San Juan: Report on file at the Consejo para la Protección del Patrimonio Arqueológico Terrestre de Puerto Rico, 1993.

50. Siegel PE. Archaeological Data Recoveries at Sites Hu-6 and Hu-7, Rio Anton Ruiz Flood Control Project, Municipio De Humacao, Puerto Rico. Report on file at the Puerto Rico State Historic Preservation Office, San Juan: 2007.

51. Weaver GG, Garrow PH, McNutt Jr. CH, Oliver JR. La Iglesia de Maraguez (PO-39): Investigations of a Local Ceremonial Center in the Cerillos River Valley, Ponce, Puerto Rico. Submitted to the U.S. Army Corps of Engineers, Jacksonville District. Report on file at the Puerto Rico State Historic Preservation Office, San Juan, Puerto Rico: 1995.

52. Espenshade CT, Young SMM, Foss JE. The Cultural Landscape of Jácana: Archaeological Investigations of Site PO-29, Municipio de Ponce, Puerto Rico. Report on file with the Oficina Estatal de Conservación Histórica, San Juan: 2012.

53. García Goyco O, Freytes Rodríguez R. Informe final fase III, Proyecto King’s Helmet, Yabucoa, Vols. 1-3. Report on file at the Consejo de Arqueología Terrestre de Puerto Rico, San Juan: 2008.

54. Ortiz H. Evaluación Arqueológica Fase II, Proyecto Industrial Febus, Bo. Hato Tejas, Bayamón. Report on file at the Consejo para la Protección del Patrimonio Arqueológico Terrestre de Puerto Rico, San Juan: 2005.

55. Maiz EJ. La fauna vertebrada del orden Chiroptera-suborden microchiroptera recuperada del sitio arqueológico La Florida (Ärea 3) en Yauco, Puerto Rico. VII Encuentro de Investigadores de Arqueología y Etnohistoria. San Juan, Puerto Rico: Instituto de Cultura Puertorriqueña; 2010. p. 96-107.

56. Muñoz Guevara LV. Informe bioarqueológico final de campo, Sitio La Gallera, Ceiba Puerto Rico. Report on file at the Consejo de Arqueología Terrestre de Puerto Rico, San Juan: 2020.

57. González Colón J. Evaluación de recursos culturales Fase II, Sitio La Haya, Lajas, Puerto Rico. Report on file at the Consejo de Arqueología Terrestre, San Juan: 2007.

58. Chanlatte Baik LA, Narganes Storde YM. Vieques, Puerto Rico: Asiento de una nueva cultura aborigen antillana. Santo Domingo, Dominican Republic: Impresora Corporán; 1983.

59. Narganes Storde YM. Sorcé, una aldea de pescadores saladoides. La Fauna y la Dieta Saladoide en la isla de Vieques. San Juan, Puerto Rico: Centro de Estudios Avanzados de Puerto Rico y el Caribe; 2015.

60. López D, Molina D. Archaeological Data Recovery at La Planta (L-19), Loíza, Puerto Rico. Report on file at the Puerto Rico State Historic Preservation Office, San Juan: 1988.

61. Solís Magaña C, Rodríguez López M. Phase II Archaeological Evaluation Site NCS-4 (La Trocha), Municipality of Vega Baja, Puerto Rico. Report on file at the Consejo para la Protección del Patrimonio Arqueológico Terrestre, San Juan: 2000.

62. Meléndez S. Archaeological Survey and National Register of Historic Places Eligibility Determination at the Aibonito Flowers Inventory Property, Asomante, Aibonito, Puerto Rico, New South Associates Technical Report #2552. Report on file at the Puerto Rico State Historic Preservation Office, San Juan: 2016.

63. Meulengracht A, Mcgovern P, Lawn B. University of Pennsylvania Radiocarbon Dates XXI. Radiocarbon. 1981;23:227-40.

64. Ramos M. Informe de Investigación Arqueológica Fase II, Parcela Lilly-Caribe, Inc, Carolina, Puerto Rico. Report on file at the Consejo para la Protección del Patrimonio Arqueológico Terrestre de Puerto Rico, San Juan, 2002.

65. Hayward MH, Cinquino MA. Archaeological Data Recovery of Prehistoric Site LO-9 for the Shoreline Protection Project Highway 187, Punta Maldonado, Municipio of Loiza, Puerto Rico. Report on file at the Puerto Rico State Historic Preservation Office, San Juan: 1998.

66. Tronolone CA, Cinquino MA, Vandrei CE. Cultural Resource Reconnaissance Survey for the Vieques Naval Reservation. San Juan: Copies available at the Puerto Rico State Historic Preservation Office, 1984.

67. Pestle WJ. Diet and Society in Prehistoric Puerto Rico, An Isotopic Approach [Unpublished Ph.D. Dissertation]: University of Illinois at Chicago; 2010.

68. Nägele K, Posth C, Iraeta Orbegozo M, Chinique de Armas Y, Hernández Godoy ST, González Herrera UM, et al. Genomic insights into the early peopling of the Caribbean. Science. 2020:eaba8697. doi: 10.1126/science.aba8697.

69. Oliver JR. Results of the Archaeological Testing and Data Recovery Investigations at the Lower Camp Site, Culebra Island National Wildlife Refuge, Puerto Rico. Report on file at the Puerto Rico State Historic Preservation Office, San Juan, Puerto Rico: 1992.

70. Rivera V, Pérez SA, editors. Estudio preliminar de la distribución espacial en la comunidad aborígen de Luján I. Proceedings of the Seventeenth Congress of the International Association for Caribbean Archaeology; 1999: Molloy College.

71. Martínez Cruzado JC. Machuca: Yacimiento arqueológico de mayor envergadura localizado en el oeste de Puerto Rico. Report in possession of the author: nd.

72. Siegel PE. Ideology, power, and social complexity in prehistoric Puerto Rico. Binghamton, NY: SUNY-Binghamton; 1992.

73. Siegel PE. Ideology and Culture Change in Prehistoric Puerto Rico: A View from the Community. Journal of Field Archaeology. 1996;23(3):313-33.

74. Siegel PE. Site Structure, Demography and Social Complexity in the Early Ceramic Age of the Caribbean In: Siegel PE, editor. Early Ceramic Population, Lifeways, and Adaptive Strategies in the Caribbean. Oxford: British Archaeological Reports; 1989. p. 193-245.

75. Siegel PE. Contested Places and Places of Contest: The Evolution of Social Power and Ceremonial Space in Prehistoric Puerto Rico. Latin American Antiquity. 1999;10(3):209-38.

76. Siegel PE. Occupational History of the Maisabel Site. Florida Journal of Anthropology. 1991;Special Publication No. 7(16):65-80.

77. Ramos M, Anderson-Córdova KF. Investigación arqueológica Fase III, Mejoras al Sistema Sanitario de la Comunidad Esperanza, Sitios Martineau y La Mina, Vieques, Puerto Rico. Report on file at the Puerto Rico State Historic Preservation Office, San Juan: 2005.

78. Rodríguez López M. Excavaciones en el yacimiento Arcaico de Maruca, Ponce, Puerto Rico: Informe Final. Copies available at the Consejo para la Protección del Patrimonio Arqueológico Terrestre de Puerto Rico, San Juan: 2004.

79. Pantel Tekakis AG. Evaluación de Recursos Culturales de Fase II, Yacimiento Precolombino de Maruca, Barrio Canas, Municipio de Ponce, Puerto Rico. Report on file at the Consejo para la Protección del Patrimonio Arqueológico Terrestre de Puerto Rico, San Juan: 1994.

80. Grossman and Associates I. Excavation and Analysis Results of Archaeological Investigations at Medianía Alta (L-23) and Vieques (L-22), Loíza, Puerto Rico. Report on file at the Puerto Rico State Historic Preservation Office, San Juan: 1990.

81. Goodwin RC, Sanders SL, Simmons MA, Davis DD, Vento F. Patterns & Transformations in the Prehistory and History of Vieques. Technical Series: Archeological Survey and Evaluation of the Vieques Naval Reservation, Municipality of Vieques, Puerto Rico. Prepared for Naval Facilities Engineering Command, Atlantic Division, Norfolk. Report on file at the Puerto Rico State Historic Preservation Office, San Juan: 2001.

82. Pestle WJ, Laguer-Díaz C, Schneider MJ, Carden M, Sherman CE, Koski-Karell D. Shellfish Collection Practices of the First Inhabitants of Southwestern Puerto Rico: The Effects of Site Type and Paleoenvironment on Habitat Choice. Latin American Antiquity. 2021;32(4):850-7.

83. Ramos M. Investigación arqueológica Fase II, Proyecto Planta de Energía Total, AES, Puerto Rico, Bo. Jobos, Guayama. Report on file at the Consejo de Arqueología Terrestre de Puerto Rico, San Juan: 1996.

84. Siegel PE, Joseph JW. Archaeological Data Recovery at El Palmar de las Animas (Site VB-27) and the Concrete Well Site (Site VB-32), Río Cibuco Flood Control Project, Municipio de Vega Baja, Puerto Rico. Report on file at the Puerto Rico State Historic Preservation Office, San Juan: 1993.

85. Meléndez MJ. Data Recovery Plan for the Palmas Altas Site Palmas Altas Ward, Barceloneta, Puerto Rico. Report on file at the Puerto Rico State Historic Preservation Office, San Juan: 2002.

86. Walker JB. The Paso del Indio Site, Vega Baja, Puerto Rico: A Progress Report. In: Siegel PE, editor. Ancient Borinquen: Archaeology and Ethnohistory of Native Puerto Rico. Tuscaloosa: The University of Alabama Press; 2005. p. 55-87.

87. García Goyco O, Solís Magaña C. Informe de fin de obras, proyecto arqueológico Paso del Indio, Vega Baja, Puerto Rico. Prepared for the Department of Transportation and Public Works, Highway and Transportation Authority, Commonwealth of Puerto Rico, San Juan. Report on file at the Consejo de Arqueología Terrestre de Puerto Rico, San Juan: 1999.

88. Pagán-Jiménez JR, Carlson LA. Recent Archaeobotanical Findings of the Hallucinogenic Snuff Cojoba (Anadenanthera Peregrin A (L.) Speg.) in Precolonial Puerto Rico. Latin American Antiquity. 2014;25(1):101-16.

89. Pérez C. Salvamento Arqueológico en Playa Jayuya, Fajardo. Report on file at the Consejo para la Protección del Patrimonio Arqueológico Terrestre de Puerto Rico, San Juan: 2014.

90. Maurás Casillas A. Mitigación arqueológica Fase III, Centro de Convenciones A.E.E.L.A, Guánica. Report on file at the Consejo de Arqueología Terrestre de Puerto Rico, San Juan: 2005.

91. Espenshade CT, Foss JE, Joseph JW. Data Recovery Excavations at PO-21, Cerrillos River Valley, Puerto Rico. Report on file at the Puerto Rico State Historic Preservation Office, San Juan: 1987.

92. Torres JM. The Social Construction of Community, Polity, and Place in Ancient Puerto Rico (AD 600-AD 1200) [Unpublished Ph.D. Dissertation]. Gainesville, FL: University of Florida; 2012.

93. González Colón J. Evaluación de Recursos Culturales Fase II, Sitio Praderas, Gurabo, Puerto Rico. Report on file at the Consejo para la Protección del Patrimonio Arqueológico Terrestre de Puerto Rico, San Juan, 2006.

94. Chanlatte Baik LA. El hombre de Puerto Ferro. Recinto de Río Piedras. Puerto Rico: Catálogo, Publicación de la Exhibición. Centro de Investigaciones Arqueológicas y Museo de la Universidad de Puerto Rico; 1991.

95. Rodríguez López M. Arqueología de Punta Candelero, Puerto Rico. In: Ayubi EN, Haviser JB, editors. Proceedings of the 13th International Congress for Caribbean Archaeology. Curaçao: Reports of the Archaeological-Anthropological Institute of the Netherlands Antilles,; 1991. p. 605-27.

96. Rodríguez López M. The Zoned Incised Crosshatch (ZIC) Ware of Early Precolumbian Ceramic Age Sites in Puerto Rico and Vieques Island. In: Siegel PE, editor. Early Ceramic Population Lifeways and Adaptive Strategies in the Caribbean. BAR International Series. 506. Oxford: British Archaeological Reports; 1989. p. 249-66.

97. Ramos M, Ortiz Aguilú JJ. Informe de investigación arqueológica Fase III, Parcela RC-1A, Punta Candelero, Humacao, Puerto Rico. Report on file at the Consejo de Arqueología Terrestre de Puerto Rico, San Juan: 2007.

98. Ramos M, Ortiz Aguilú JJ. Informe de investigación arqueológica Fase III, Punta Guayanés Fase I, Bo. Playa, Yabucoa, Puerto Rico. Report on file at the Consejo de Arqueología Terrestre de Puerto Rico, San Juan: 2005.

99. Muñoz Guevara LV. Estudio bioarqueológico del sitio prehispánico Punta Mameyes (Do-42), Dorado, Puerto Rico: Centro de Estudios Avanzados de Puerto Rico y el Caribe, San Juan; 2014.

100. Vega J. Archaeological Mitigation Stage III, Improvements to the Sanitary System of Punta Santiago, Humacao, Puerto Rico. Report on file at the Consejo de Arqueología Terrestre de Puerto Rico, San Juan: 2006.

101. Goodwin RC, Oliver JR, Davis DD, Brown J, Sanders Z, Simmons MA. Evaluation of Prehistoric Site Rio Cocal 1. Report on file at the Puerto Rico State Historic Preservation Office, San Juan: 2003.

102. Carlson LA, Antón S, Rodríguez Ramos R, Foss JE, Windingstad J, Cordell AS, et al. A Multidisciplinary Approach to Site Testing and Data Recovery at Two Village Sites (AR-38 and AR-39) on the Lower Río Tanamá, Municipality of Arecibo, Puerto Rico. Report on file at the Puerto Rico State Historic Preservation Office, San Juan: Southeastern Archaeological Research, Inc., 2007.

103. Sara TR, McClintock S. Archaeological Survey and Site Evaluations, Naval Station Roosevelt Roads, Puerto Rico. Miscellaneous Report of Investigations Number 323. Submitted to the Department of the Navy, Naval Facilities Engineering Command, Atlantic Division, Norfolk, Virginia, by Geo‐Marine, Inc.: 2005.

104. Narganes Storde YM. Secuencia cronológica de Dos Sitios Arqueológicos de Puerto Rico (Sorcé, Vieques y Tecla, Guayanilla). Actas del 13 Congreso de Arqueología del Caribe; Curazao1991.

105. García Goyco O, Freytes Rodríguez R, Questell E. Informe final editado, evaluación arqueológica fase II, Proyecto El Tesoro de Dorado, Barrio Lajas, Dorado, Puerto Rico. Report on file at the Consejo de Arqueología Terrestre de Puerto Rico: 2007.

106. Curet LA. The Archaeological Project of the Ceremonial Center of Tibes, 1998-2001. In: Curet LA, Stringer LM, editors. Tibes: People, Power, and Ritual at the Center of the Cosmos. Tuscaloosa, Alabama: University of Alabama Press; 2010. p. 38-59.

107. González Colón J. Tibes: Un Centro Ceremonial Indígena. San Juan, Puerto Rico: Centro de Estudios Avanzados de Puerto Rico y el Caribe; 1984.

108. Rouse I, Allaire L. Caribbean. In: Meighan C, editor. Chronologies in New World Archaeology. New York: Academic Press 1978. p. 431-81.

109. Goodwin RC, Walker J. Villa Taina de Boqueron: The Excavation of an Early Taino Site in Puerto Rico. San Juan, Puerto Rico: Interamerican University Press; 1975.
