# Supplementary material for: Revision of the cultural chronology of precolonial Puerto Rico: A Bayesian approach
Source: PLoS One. 2023 Feb 22;18(2):e0282052. doi: 10.1371/journal.pone.0282052 (PMC9946257; doi:10.1371/journal.pone.0282052)
Supplement: S2 File — (DOCX) [file pone.0282052.s014.docx]

DeltaR calculation

Regionally appropriate ΔR values were calculated for the north (Atlantic) and south (Caribbean) coasts of the island using the protocols provided in DiNapoli, et al. (2020).

For the south/Caribbean coast, a relatively large suite of dates (n=44) were available for calculation of a local ΔR. The overwhelming majority (41/44, 93%) were from *Montastraea faviolata* at Turrumote Reef offshore from La Parguera, Puerto Rico and published by Kilbourne and colleagues (2007). The remaining three date pairs (shell/charcoal) were generated by L. Antonio Curet and William J. Pestle from archaeological contexts at the site of Tibes, Ponce, Puerto Rico. All forty-four individual ΔR assays used are presented below, and the final summary value determined was -138±23.

For the north/Atlantic coast, far fewer samples (n=4) were available for determination of an appropriate local ΔR. Indeed, of the four dates, three were from the island of Hispaniola and only one from Puerto Rico. Details for the four dates are presented below, with a final summary ΔR of -241±81.

Marine carbon contribution estimation

All human bone samples (and AA-72879, a canine bone sample from Punta Candelero) were calibrated using a mixed terrestrial/marine curve with an a priori stipulation of 30±15% marine carbon. The marine contribution estimate was based on the model-based isotopic reconstruction of the paleodiet of some 229 Ceramic Age and 5 pre-Arawak individuals (Pestle, et al. In Press a, b). While average modeled marine contribution to the diets of these individuals ranged from 11–28%, we decided to stipulate a slightly higher average marine contribution to diet (30%), and a large error ranges (±15%) to account for the complexities of modeling marine contributions to diet in an environment with an incredibly complex marine isoscape, which might serve to lead to underestimation of marine contribution to diet from such model-based approaches (Pestle 2013, Pestle and Laffoon 2018).
