# Supplementary figures and images for: Revision of the cultural chronology of precolonial Puerto Rico: A Bayesian approach
Source: PLoS One. 2023 Feb 22;18(2):e0282052. doi: 10.1371/journal.pone.0282052 (PMC9946257; doi:10.1371/journal.pone.0282052)

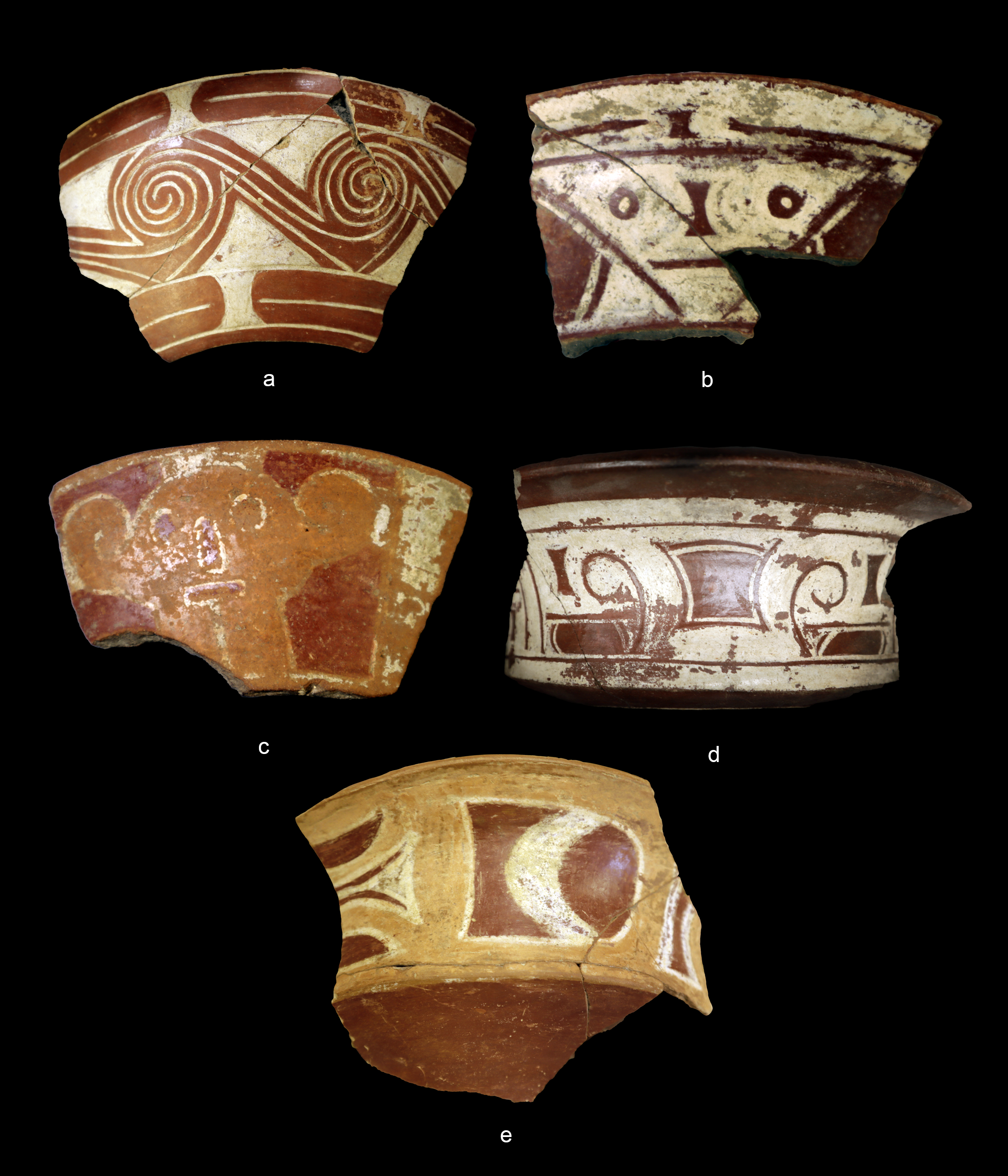

Supplement: S1 Fig — (TIF) [file pone.0282052.s015.tif]

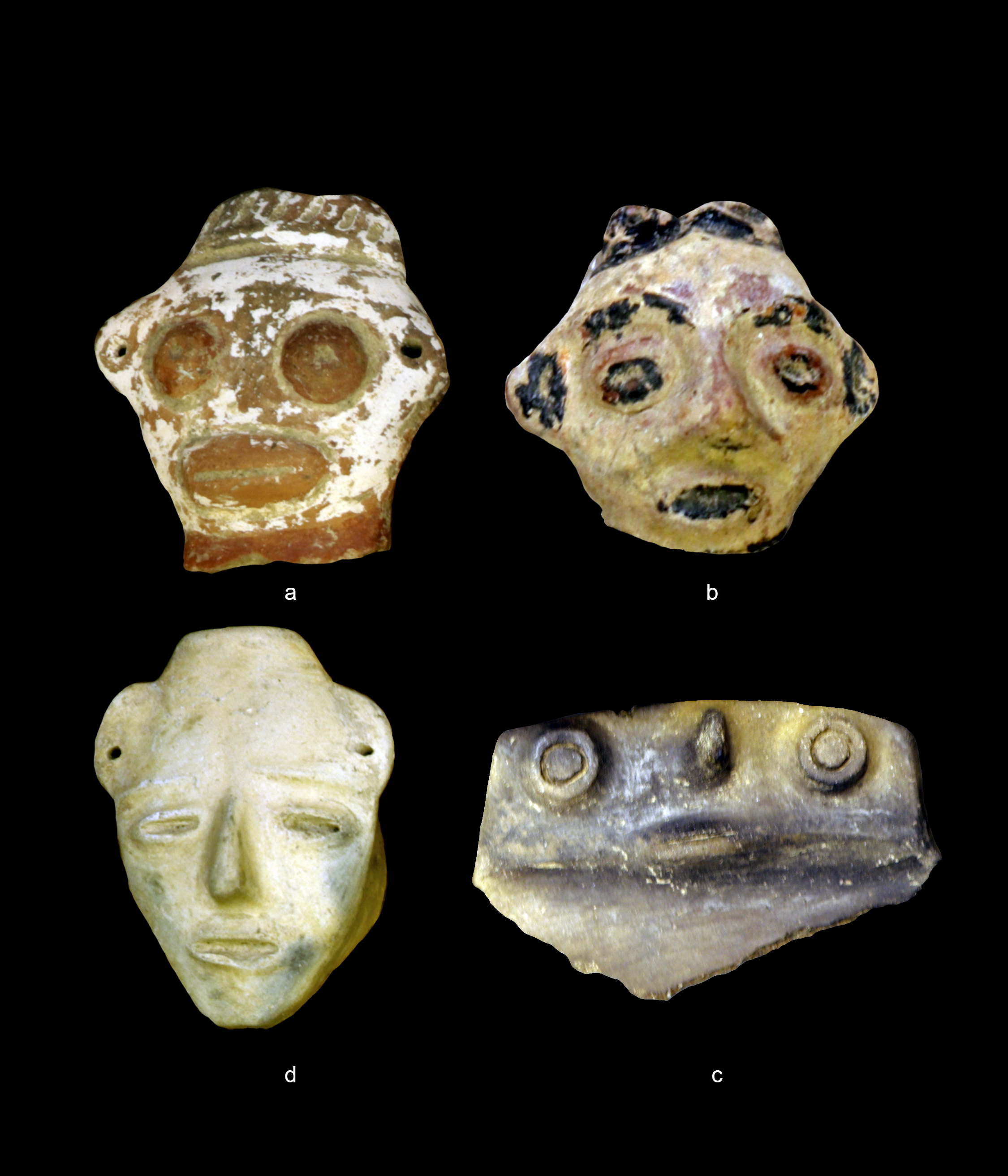

Supplement: S2 Fig — (Collection of the Centro de Investigaciones Arqueológicas, Universidad de Puerto Rico, Recinto de Río Piedras). (TIF) [file pone.0282052.s016.tif]

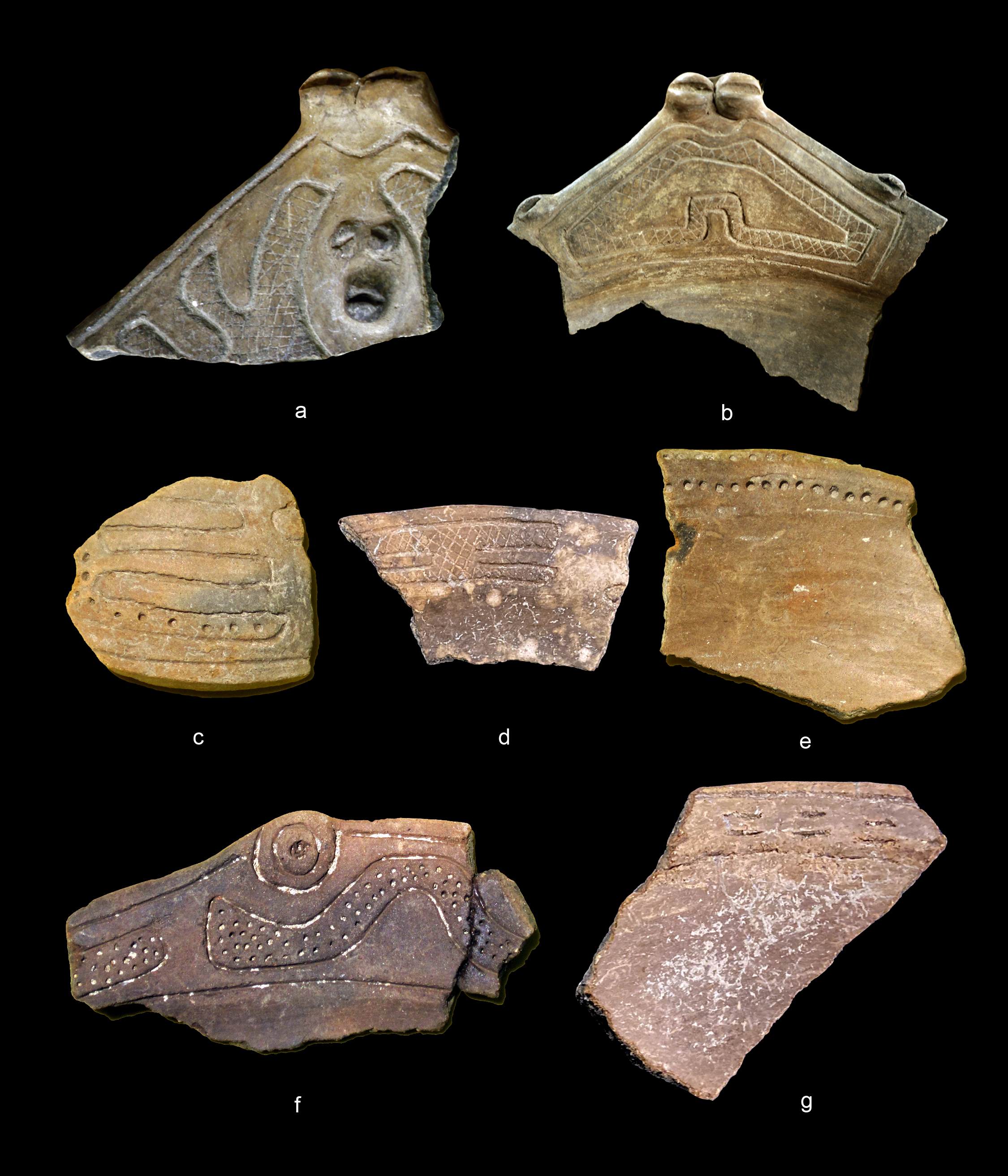

Supplement: S3 Fig — (Collection of the Centro de Investigaciones Arqueológicas, Universidad de Puerto Rico, Recinto de Río Piedras). (TIF) [file pone.0282052.s017.tif]

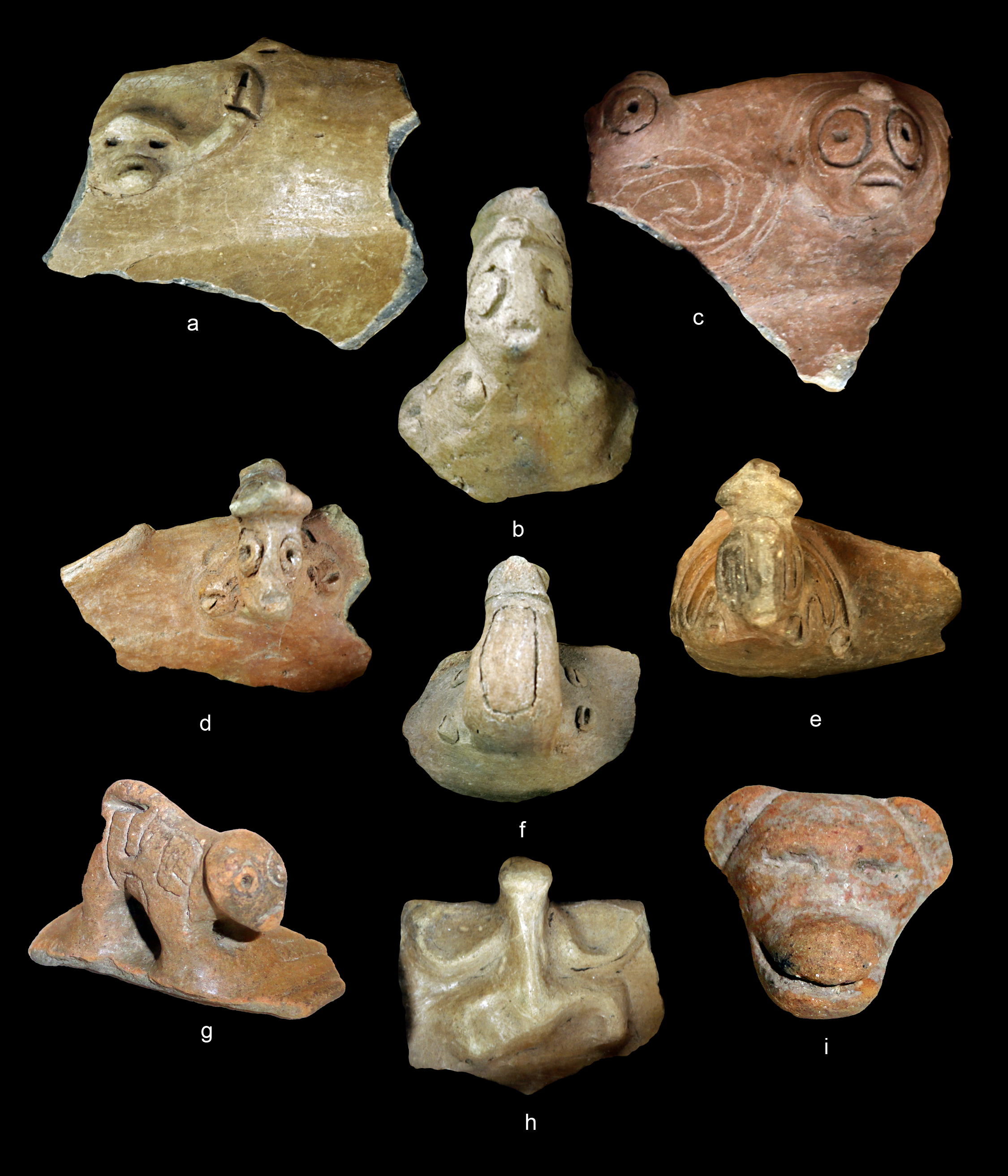

Supplement: S4 Fig — (TIF) [file pone.0282052.s018.tif]

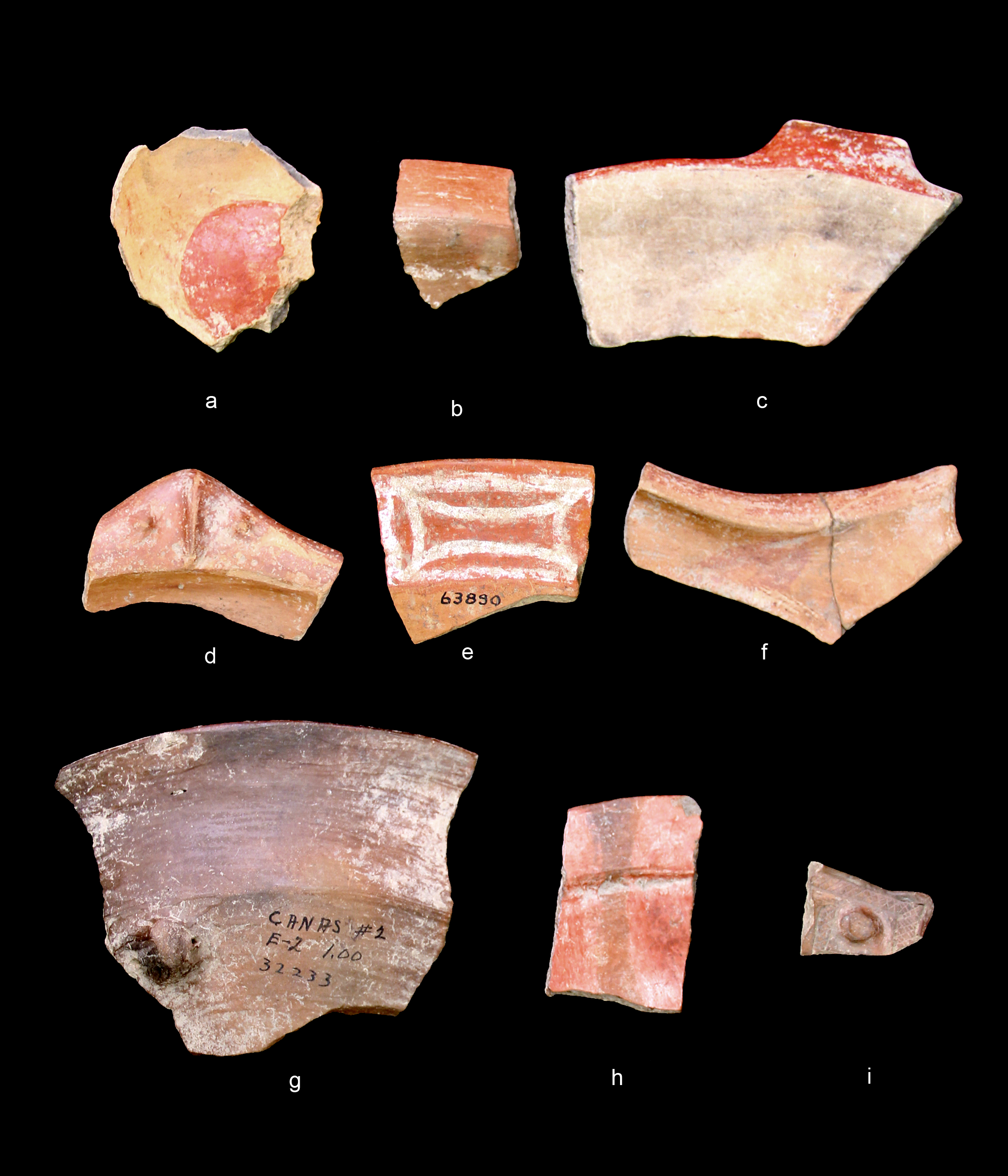

Supplement: S5 Fig — Note that artifacts ANT.85033, ANT.32089, and ANT.32433, which were included in Rouse’s original image of pottery of the Cuevas style, were excluded from this figure as they are of the Hacienda Grande style, which he had not yet defined at the time of his publication (adapted from photo courtesy of Madeliz Gutierrez Ortiz) (a. ANT.63975; b. ANT.36513; c. ANT.36513; d. ANT.85218; e. ANT.63890; f. ANT.85584; g. ANT.32233; h. ANT.98061; i. ANT.31533; Collection of the Yale Peabody Museum, Division of Anthropology). (TIF) [file pone.0282052.s019.tif]

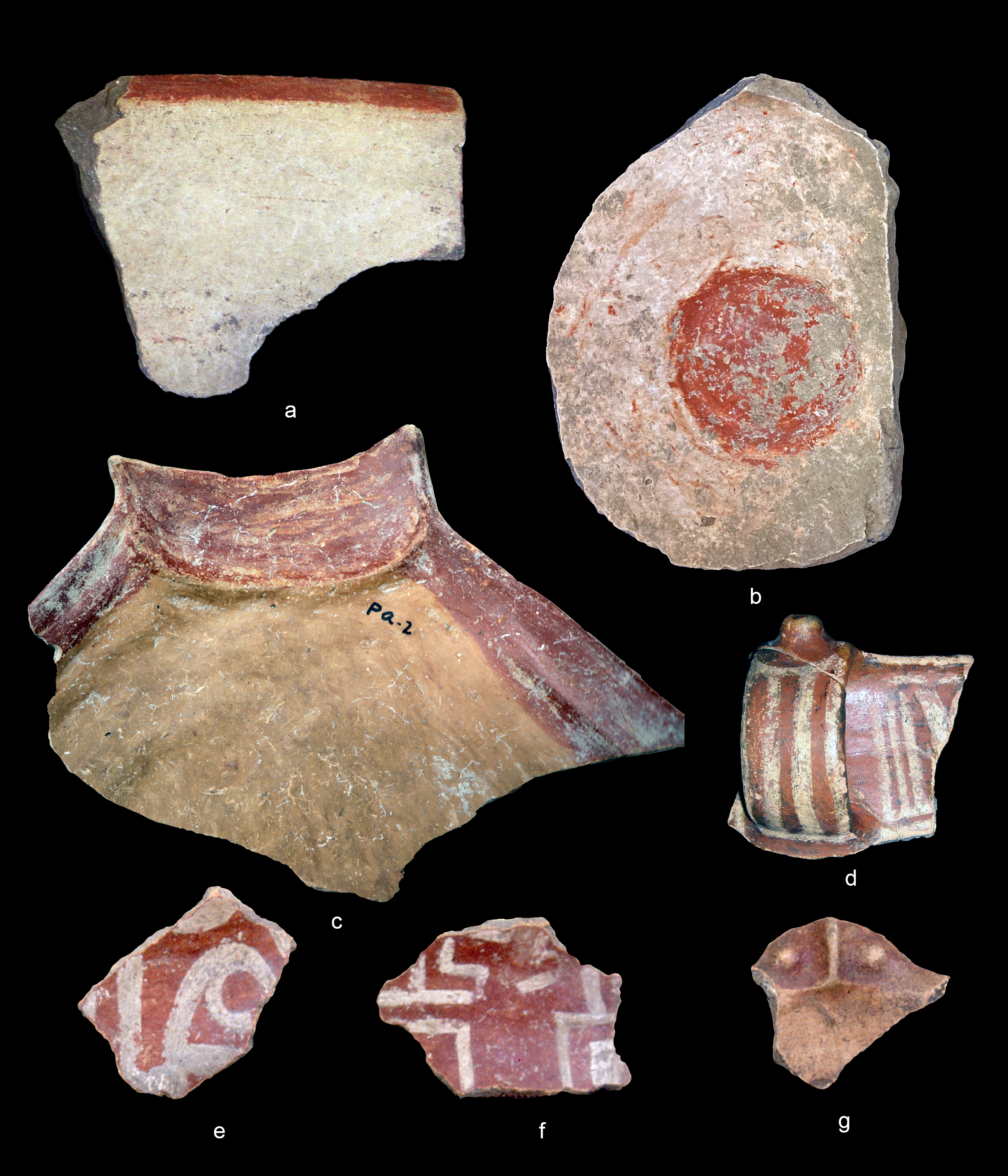

Supplement: S6 Fig — Pottery of the Cuevas style (a, b, d, e, f, g are from Collection of the Universidad Ana G. Méndez, Recinto de Gurabo; c is from the Centro de Investigaciones Arqueológicas, Universidad de Puerto Rico, Recinto de Río Piedras). (TIF) [file pone.0282052.s020.tif]

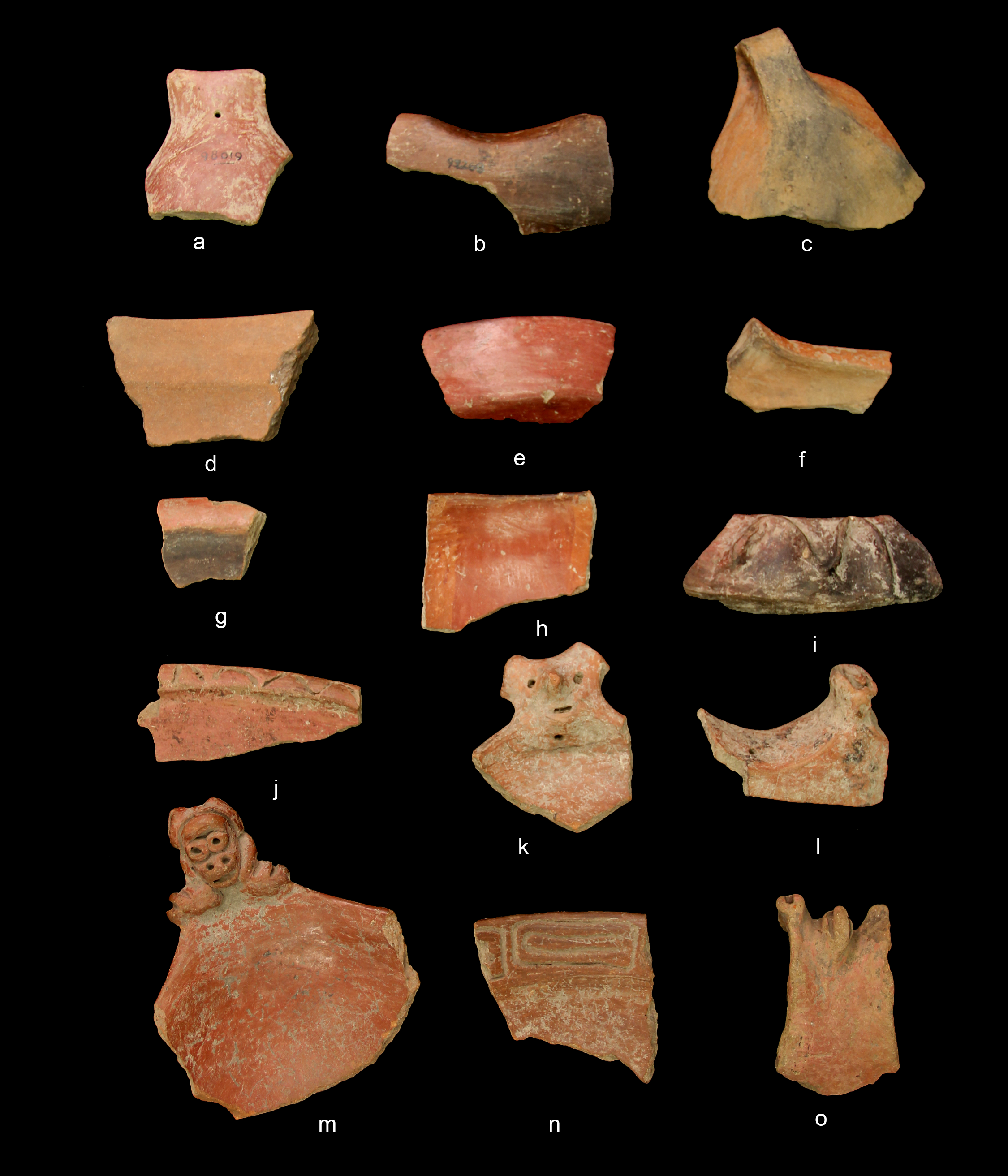

Supplement: S7 Fig — (adapted from photo courtesy of Madeliz Gutierrez Ortiz) (a. ANT.98019; b. ANT.97268; c. ANT.77752; d. ANT.83152; e. ANT.81368; f. ANT.66136; g. ANT.66407; h. ANT.82213; i. ANT.81348; j. ANT.81074; k. ANT.80989; l. ANT.81132; m. ANT.34563; n. ANT.97722; o. ANT.80231; Collection of the Yale Peabody Museum, Division of Anthropology). (TIF) [file pone.0282052.s021.tif]

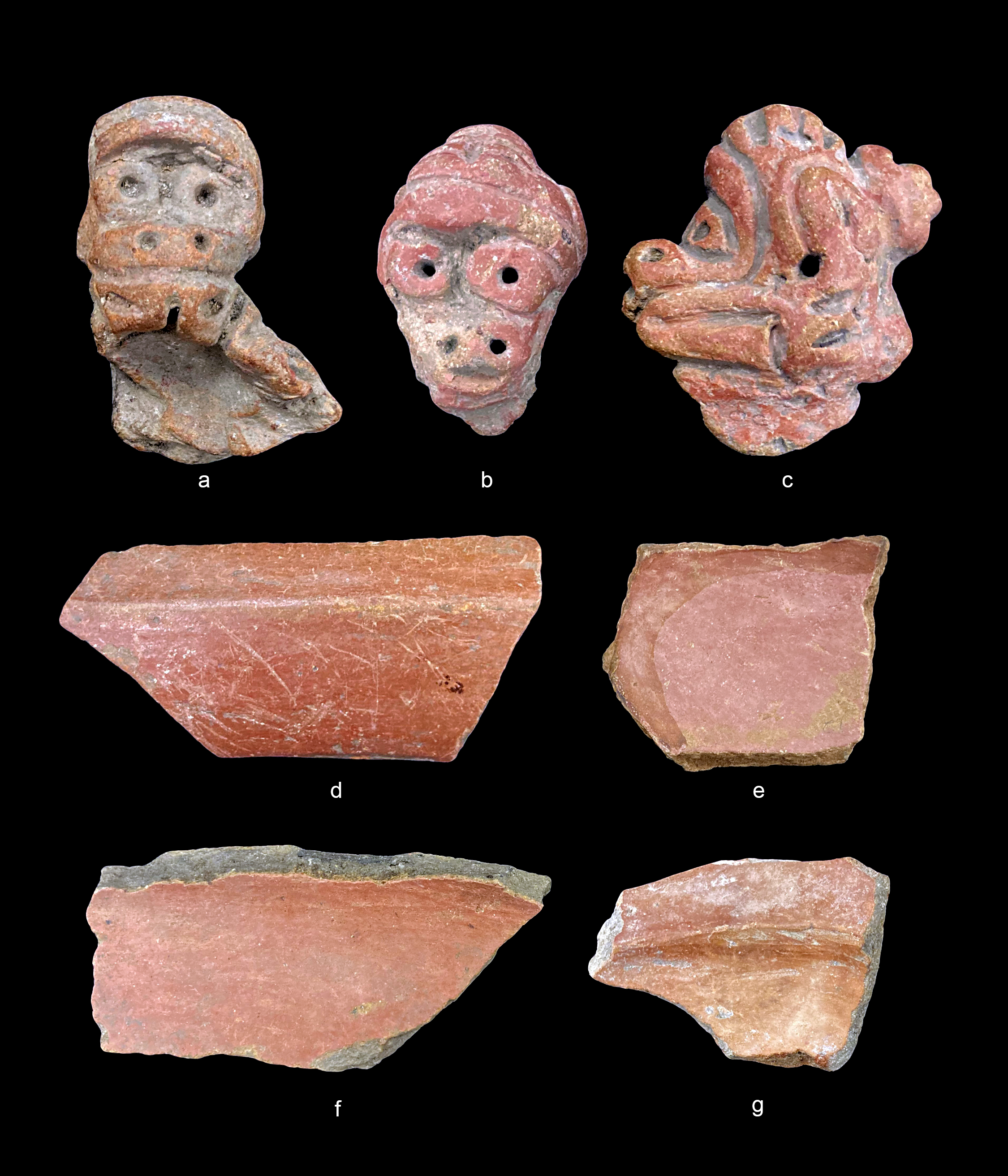

Supplement: S8 Fig — Pure) Ostiones style (Collection of the Laboratorio de Arqueología, Departamento de Sociología y Antropología, Universidad de Puerto Rico, Recinto de Río Piedras). (TIF) [file pone.0282052.s022.tif]

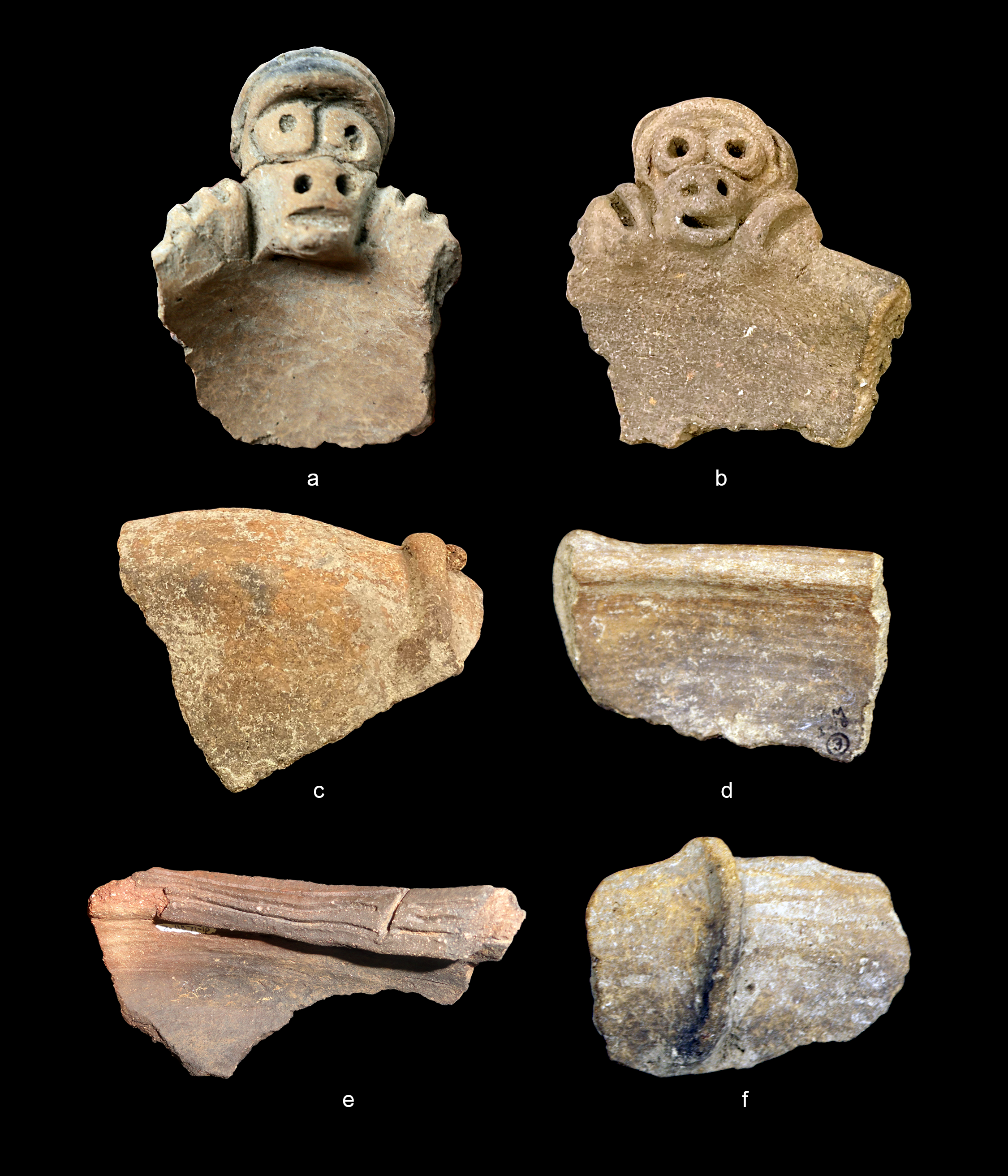

Supplement: S9 Fig — Modified) Ostiones style (a is from the Collection of the Centro de Investigaciones Arqueológicas, Universidad de Puerto Rico, Recinto de Río Piedras; b, c, d and f are from the Collection of the Laboratorio de Arqueología, Departamento de Sociología y Antropología, Universidad de Puerto Rico, Recinto de Río Piedras; e is from the Collection of the Laboratorio de Arqueología, Universidad de Puerto Rico, Recinto de Utuado). (TIF) [file pone.0282052.s023.tif]

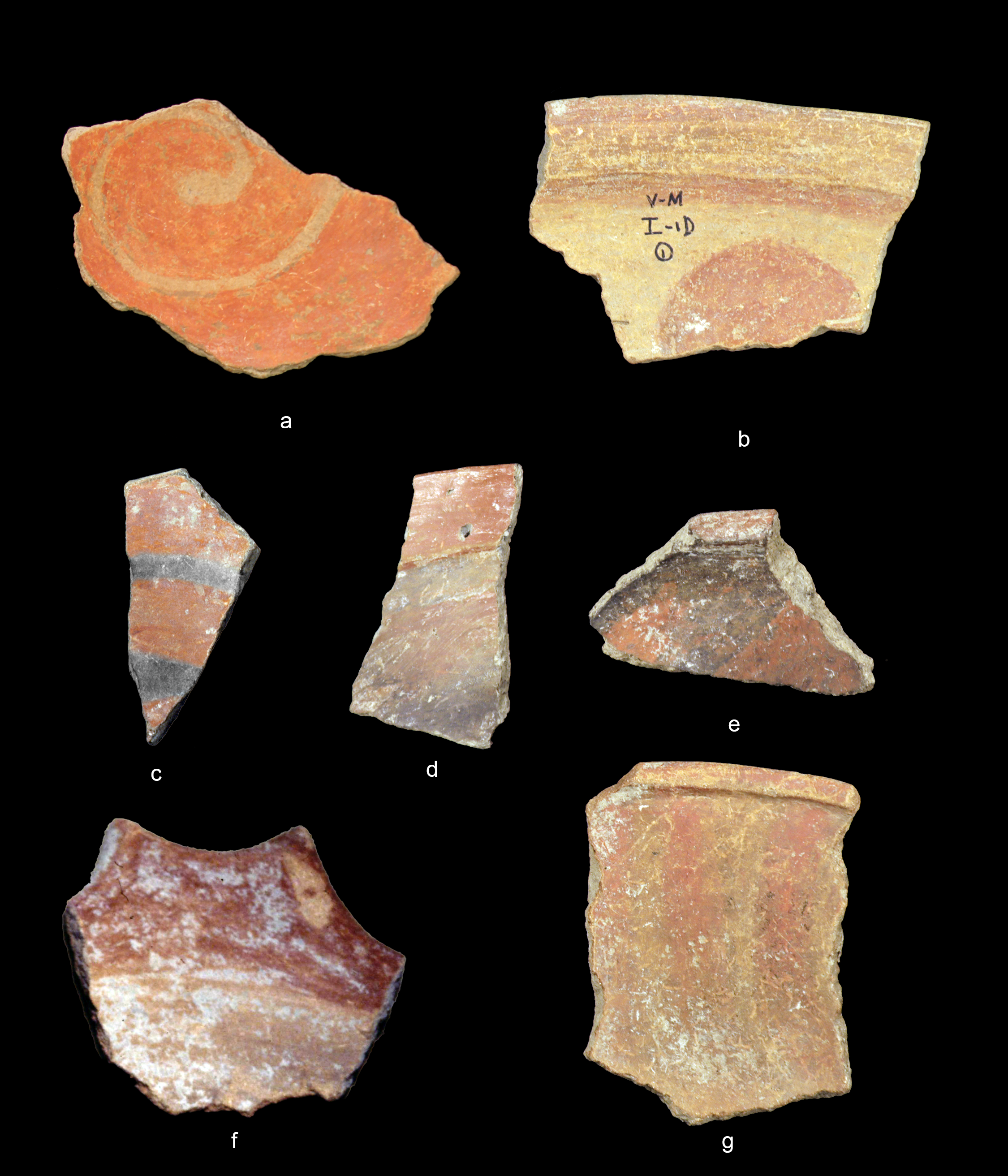

Supplement: S10 Fig — Note that artifact a (ANT.35452; Collection of the Yale Peabody Museum, Division of Anthropology), which Rouse [1:447] had originally included in his original plate within the Santa Elena style, is presented in this image as it is of the Monserrate style that he had not yet defined at the time of his publication (b-e, g. Collection of the Laboratorio de Arqueología, Universidad de Puerto Rico, Recinto de Río Piedras; f. Collection of the Universidad Ana G. Méndez, Recinto de Gurabo). (TIF) [file pone.0282052.s024.tif]

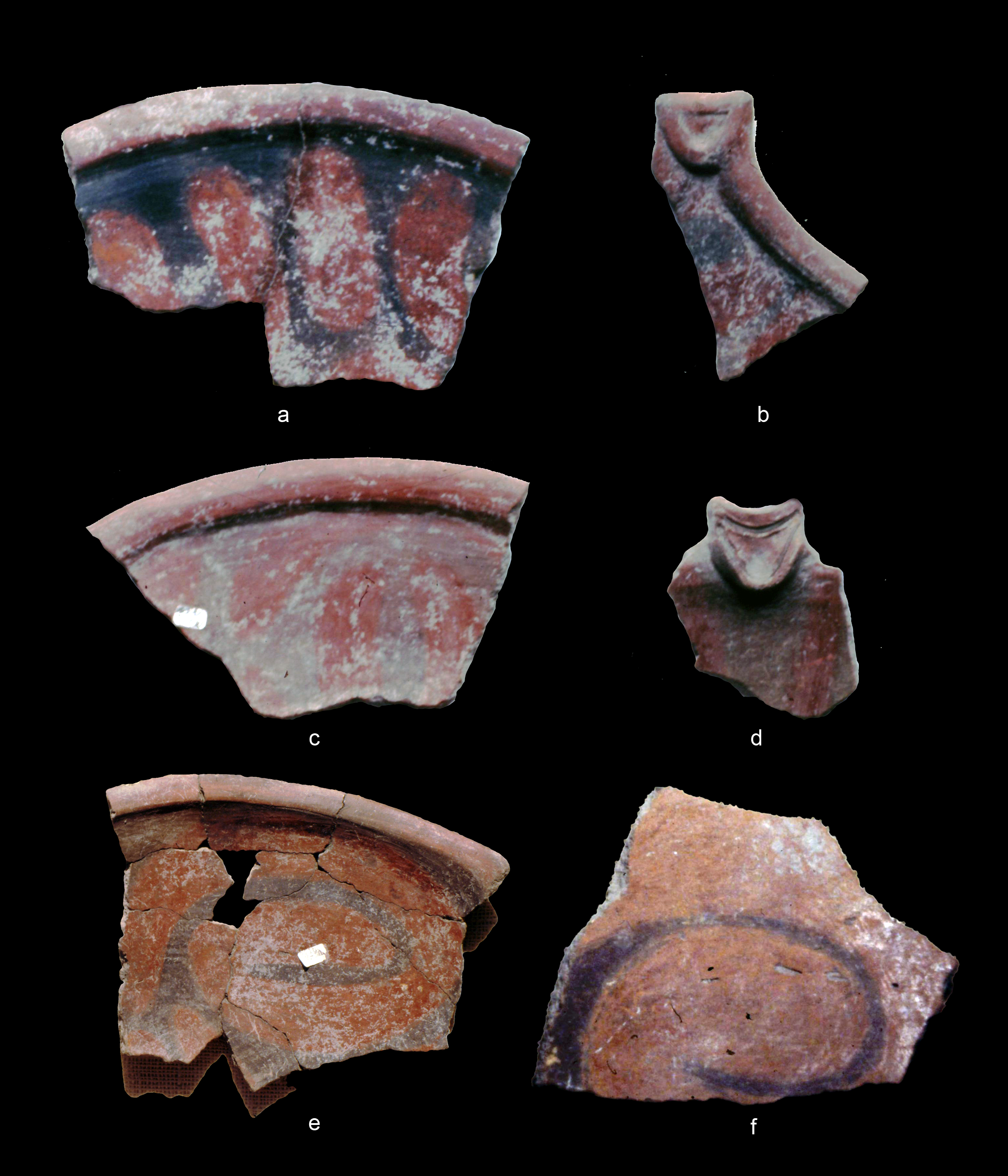

Supplement: S11 Fig — Méndez, Recinto de Gurabo). (TIF) [file pone.0282052.s025.tif]

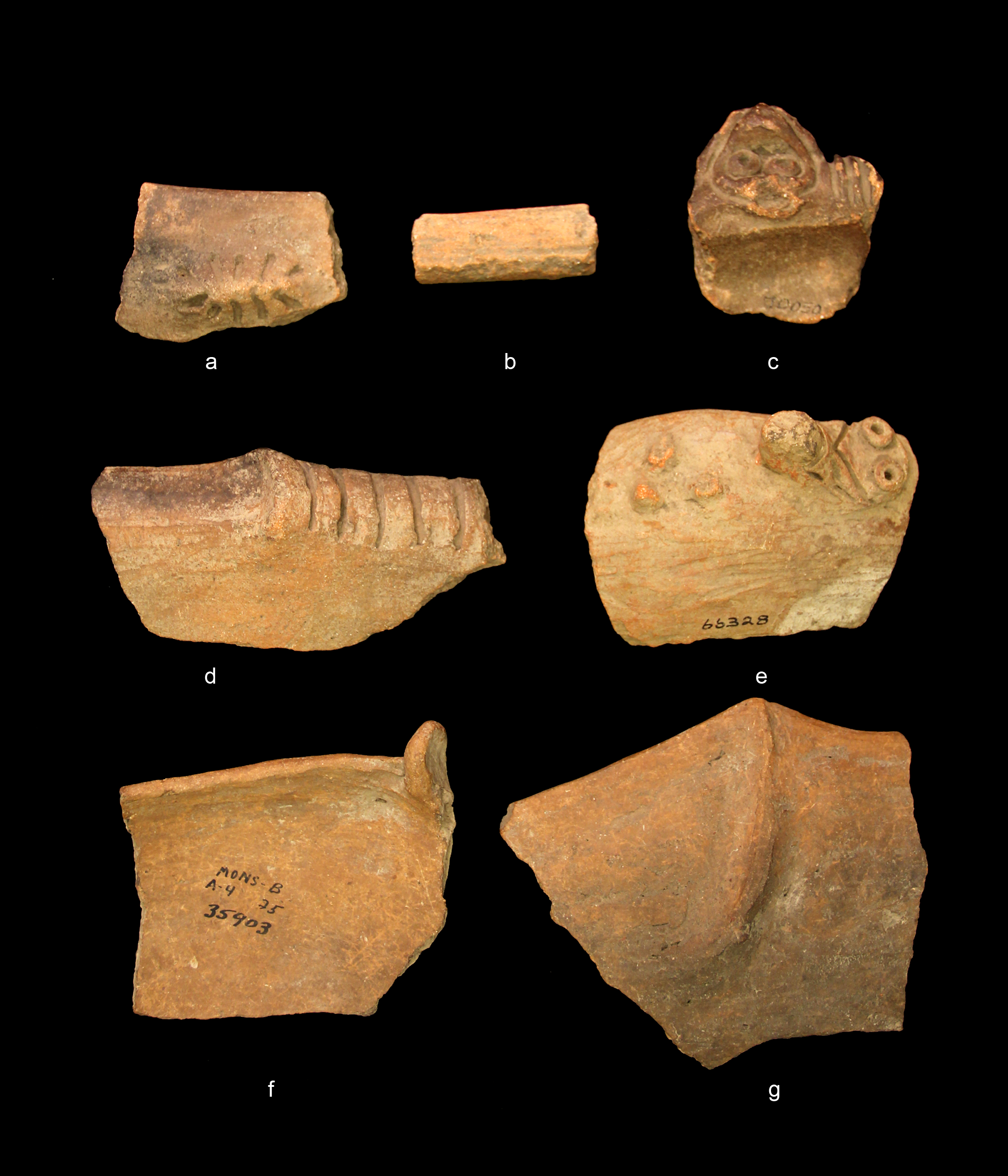

Supplement: S12 Fig — Note that artifacts ANT.35452 and ANT.96501 are excluded from this image as they are from the Monserrate style, which he had not yet defined at the time of his publication (adapted from photo courtesy of Madeliz Gutierrez Ortiz) (a. ANT.88358; b. ANT.88219; c. ANT.70050; d. ANT.88167; e. ANT.66328; f. ANT.35903; g. ANT.33896; Collection of the Yale Peabody Museum, Division of Anthropology). (TIF) [file pone.0282052.s026.tif]

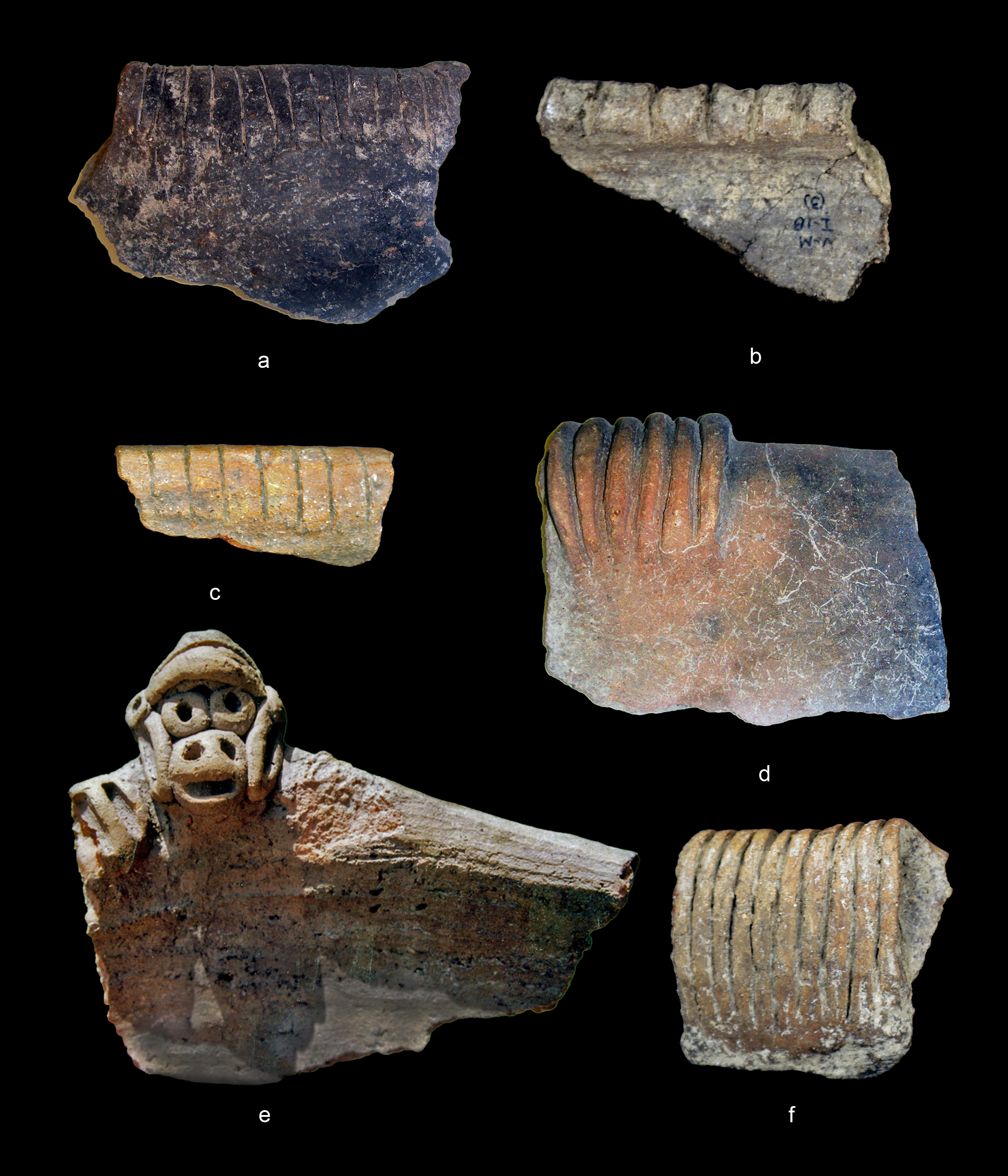

Supplement: S13 Fig — Méndez, Recinto de Gurabo). (TIF) [file pone.0282052.s027.tif]

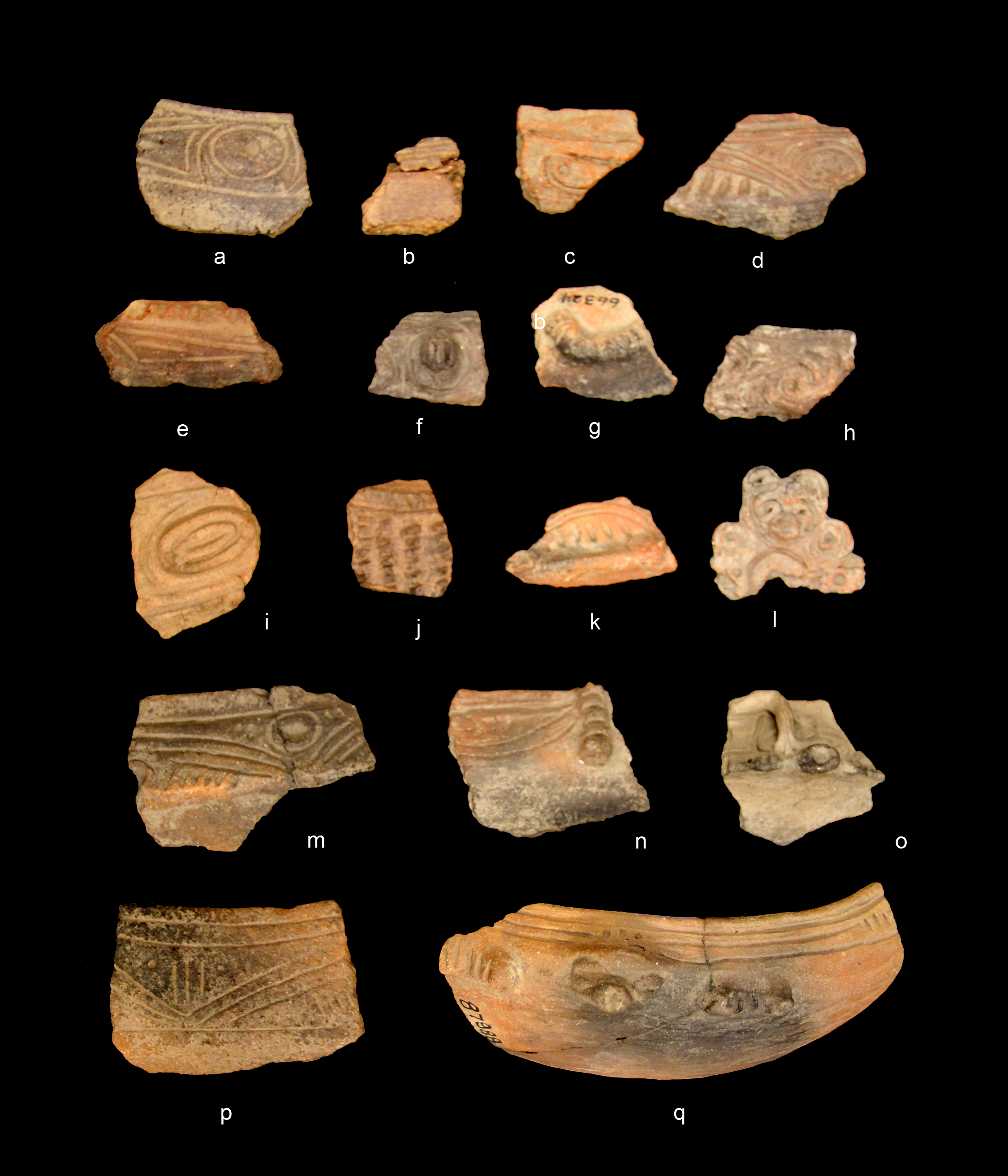

Supplement: S14 Fig — (adapted from photo courtesy of Madeliz Gutierrez Ortiz) (a. ANT.89287; b. ANT.92883; c. ANT.74186; d. ANT.87779; e. ANT.93052; f. ANT.87870; g. ANT.66324; h. ANT.74914; i. ANT.96943; j. ANT.91903; k. ANT.87860; l. ANT.75631; m. ANT.87355; n. ANT.88455; o. ANT.66157; p. ANT.89234; q. ANT.87988; Collection of the Yale Peabody Museum, Division of Anthropology). (TIF) [file pone.0282052.s028.tif]

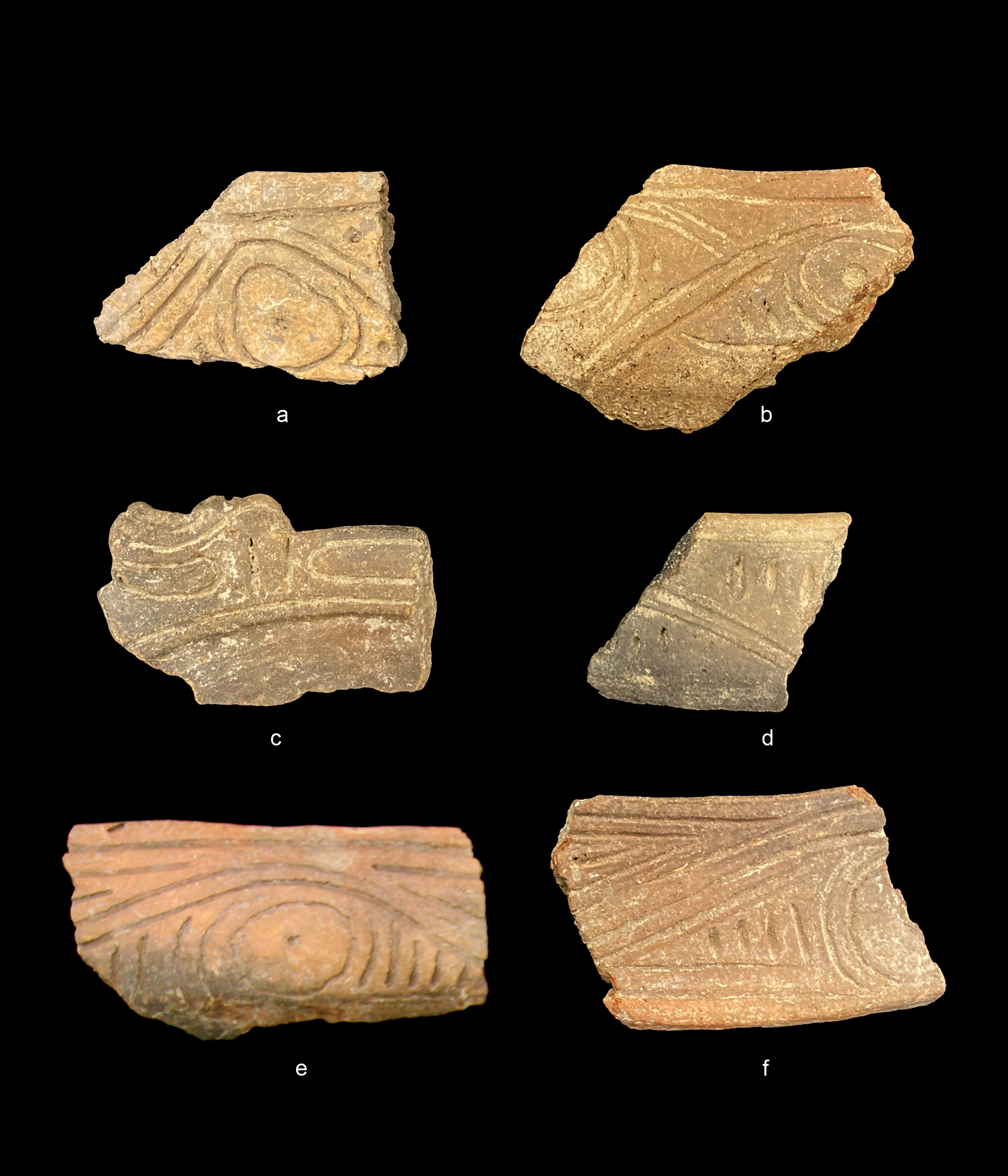

Supplement: S15 Fig — (a-d, f. Laboratorio de Arqueología, Departamento de Sociología y Antropología, Universidad de Puerto Rico, Recinto de Río Piedras. Note that object e [ANT.91615; Yale Peabody Museum, Division of Anthropology], which was originally included by Rouse [1:449] as part of the Boca Chica style, is hereby considered to be of the Capá style. (TIF) [file pone.0282052.s029.tif]

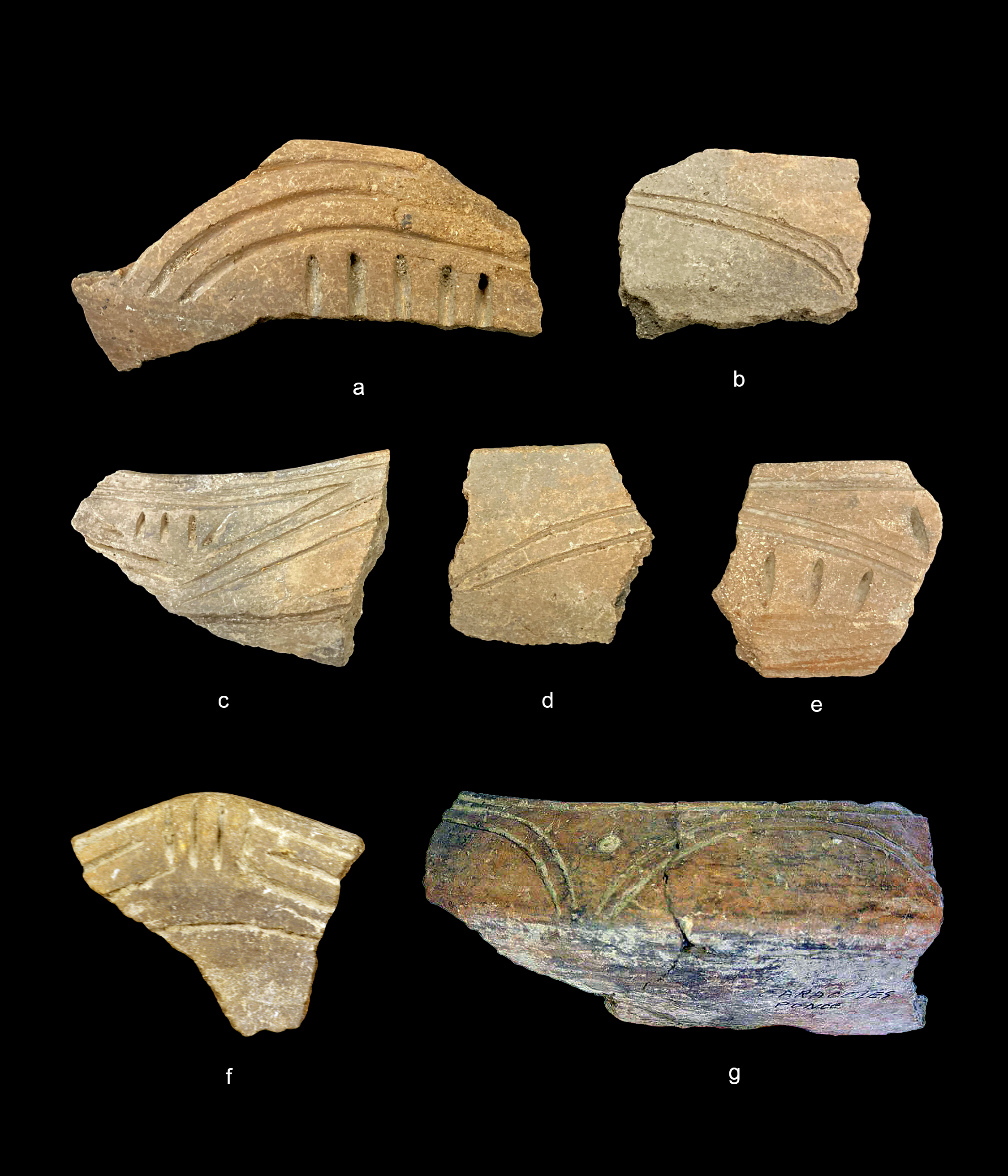

Supplement: S16 Fig — Pottery of the Capá style (a-f. Laboratorio de Arqueología, Universidad de Puerto Rico, Recinto de Río Piedras; g, modified from Rodríguez López [64]). (TIF) [file pone.0282052.s030.tif]

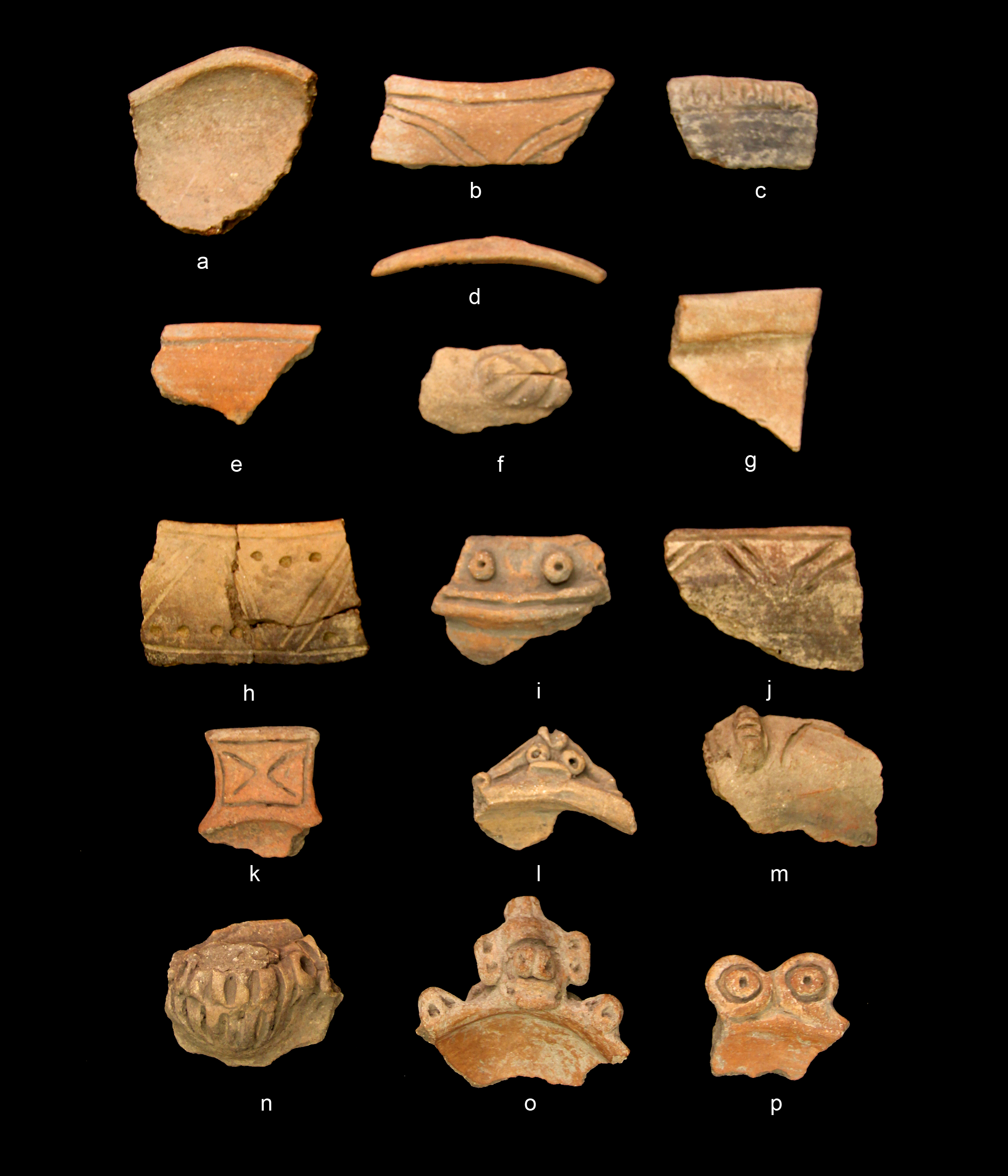

Supplement: S17 Fig — (adapted from photo courtesy of Madeliz Gutierrez Ortiz) (a. ANT.90898; b. ANT.90776; c. ANT.90955; d. ANT.90774; e. ANT.67625; f. ANT.93152; g. ANT.90713; h. ANT.66072; i. ANT.67148; j. ANT.90785; k. ANT.67949; l. ANT.67065; m. ANT.90680; n. ANT.90810; o. ANT.90809; p. ANT.90970; Collection of the Yale Peabody Museum, Division of Anthropology). (TIF) [file pone.0282052.s031.tif]

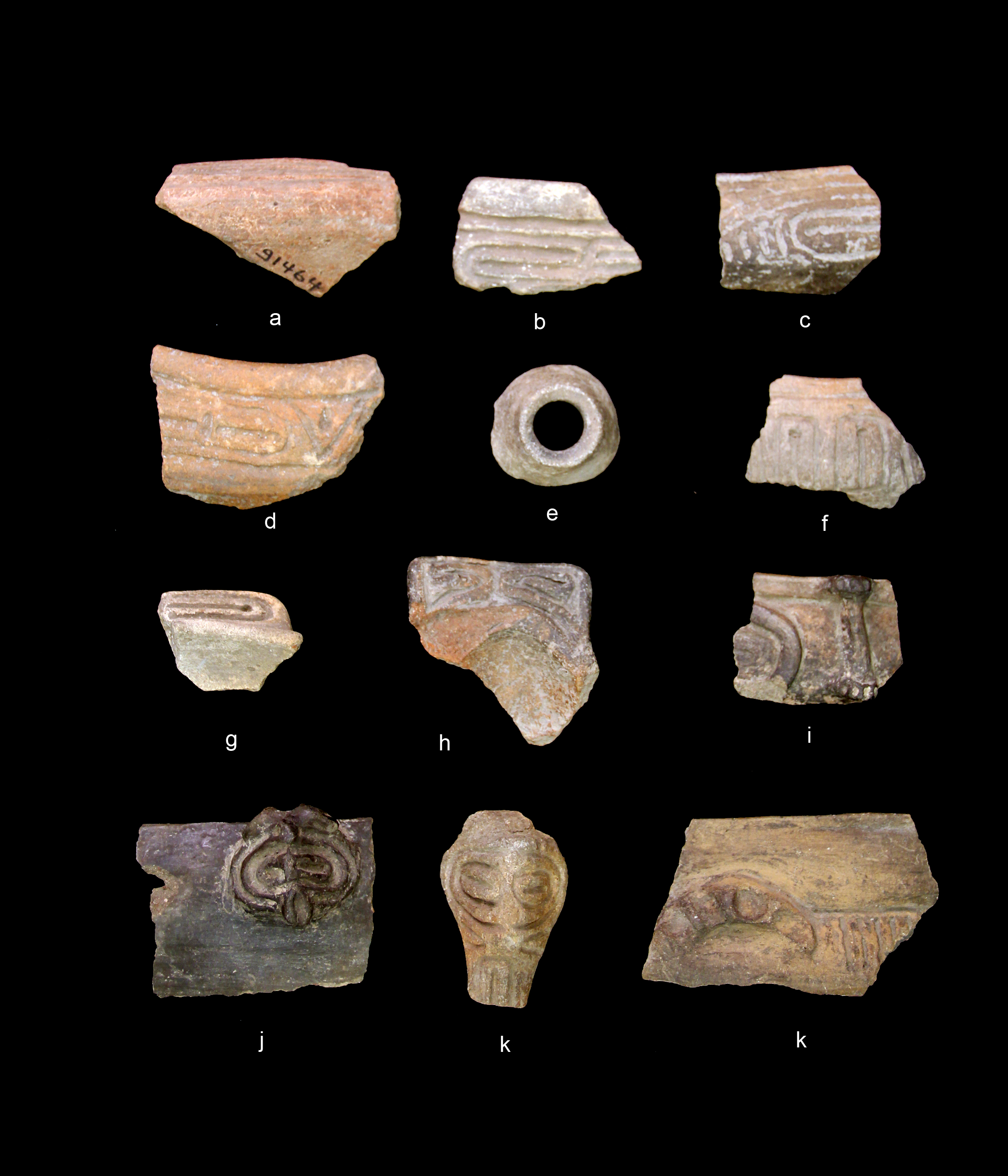

Supplement: S18 Fig — Note that we excluded object ANT.91615 from this figure as we consider it to be of the Capá style (adapted from photo courtesy of Madeliz Gutierrez Ortiz) (a. ANT.91615; b. ANT.69895; c. ANT.91340; d. ANT.91728; e. ANT.91762; f. ANT.91376; g. ANT.91746; h. ANT.91344; i. ANT.70013; j. ANT.699951; k. ANT.91482; l. ANT.91281; Collection of the Yale Peabody Museum, Division of Anthropology). (TIF) [file pone.0282052.s032.tif]
